# Supplementary figures and images for: The effect of adherence to spectacle wear on early developing literacy: a longitudinal study based in a large multiethnic city, Bradford, UK
Source: BMJ Open. 2018 Jun 12;8(6):e021277. doi: 10.1136/bmjopen-2017-021277 (PMC6009541; doi:10.1136/bmjopen-2017-021277)

## Supplementary Information 1

### Keeler Crowded LogMAR Test

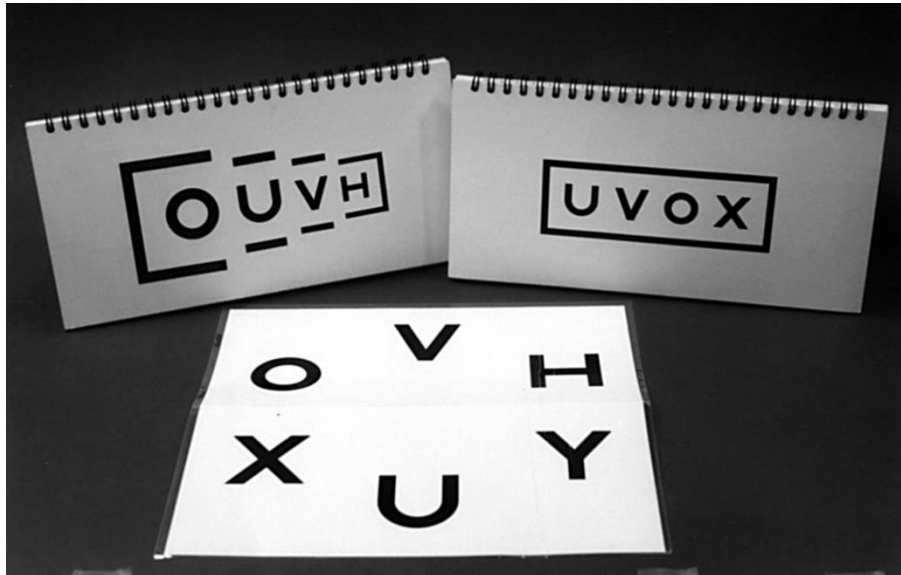

### LogMAR Near Vision Test

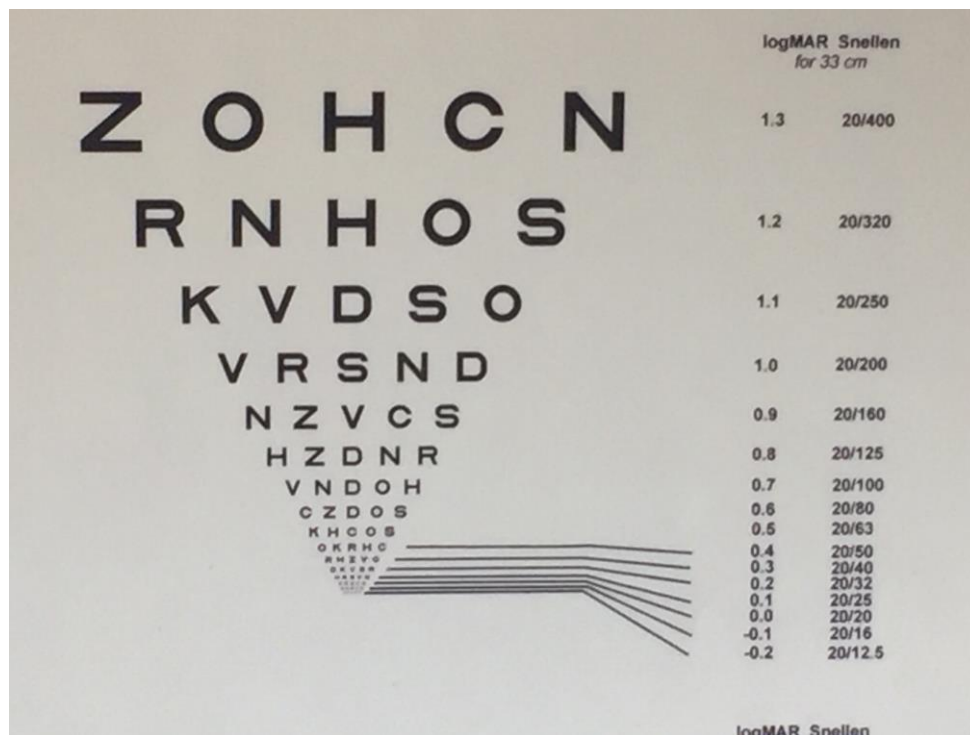

Supplement: Supplementary file 1 [file bmjopen-2017-021277supp001.pdf]
