# Supplementary material for: The effect of adherence to spectacle wear on early developing literacy: a longitudinal study based in a large multiethnic city, Bradford, UK
Source: BMJ Open. 2018 Jun 12;8(6):e021277. doi: 10.1136/bmjopen-2017-021277 (PMC6009541; doi:10.1136/bmjopen-2017-021277)
Supplement: Supplementary file 2 [file bmjopen-2017-021277supp002.pdf]

Supplementary Information 2

Associations between Letter-ID score and refractive error types.

FACTOR	FULLY ADJUSTED MODEL (95% CI)	p value
Constant	-21.4 (-29.0 to -13.8)	<0.001
Age	1.32 (1.23 to 1.41)	<0.001
Age squared	-0.021 (-0.023 to -0.018)	<0.001
**Astigmatism**	**-0.329 (-0.933 to 0.275)**	**0.286**
**Hypermetropia**	**-1.071 (-2.586 to 0.444)**	**0.166**
**Myopia**	**1.386 (-2.953 to 5.275)**	**0.531**
**Low hypermetropia**	**0.255 (-0.835 to 1.344)**	**0.647**
Letter ID baseline (Year 1)	0.346 (0.323 to 0.369)	<0.001
BPVS	0.024 (0.004 to 0.044)	0.019
Ethnicity		
Pakistani heritage	0.569 (-0.128 to 1.267)	0.11
Other	1.057 (0.037 to 2.078)	0.042
Gender		
Female	0.667 (0.102 to 1.232)	0.021
Birth weight (per 100g)	0.074 (0.007 to 0.14)	0.029
Gestational age (weeks)	-0.04 (-0.244 to 0.163)	0.698
Receiving Benefits	-0.011 (-0.588 to 0.565)	0.969
Mothers Level of Education		
(higher than A-level)	0.717 (0.11 to 1.325)	0.021
Mothers age at birth (years)	-0.054 (-0.107 to -0.002)	0.042
