# Supplementary material for: The effect of adherence to spectacle wear on early developing literacy: a longitudinal study based in a large multiethnic city, Bradford, UK
Source: BMJ Open. 2018 Jun 12;8(6):e021277. doi: 10.1136/bmjopen-2017-021277 (PMC6009541; doi:10.1136/bmjopen-2017-021277)

Supplementary Information 3

Correlation between near and distance visual acuity (Right Eye) at Time Point Three.

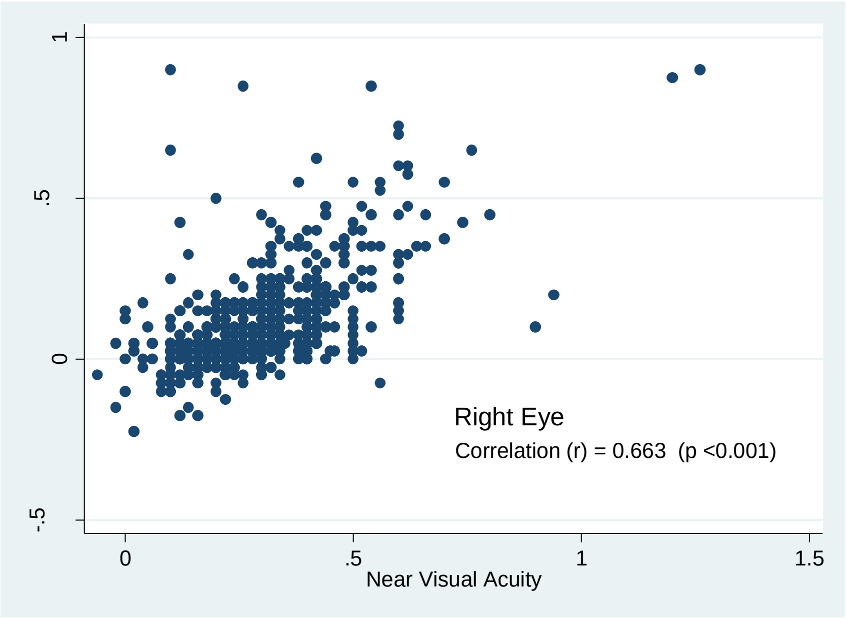

Correlation between near and distance visual acuity (Left Eye) at Time Point Three.

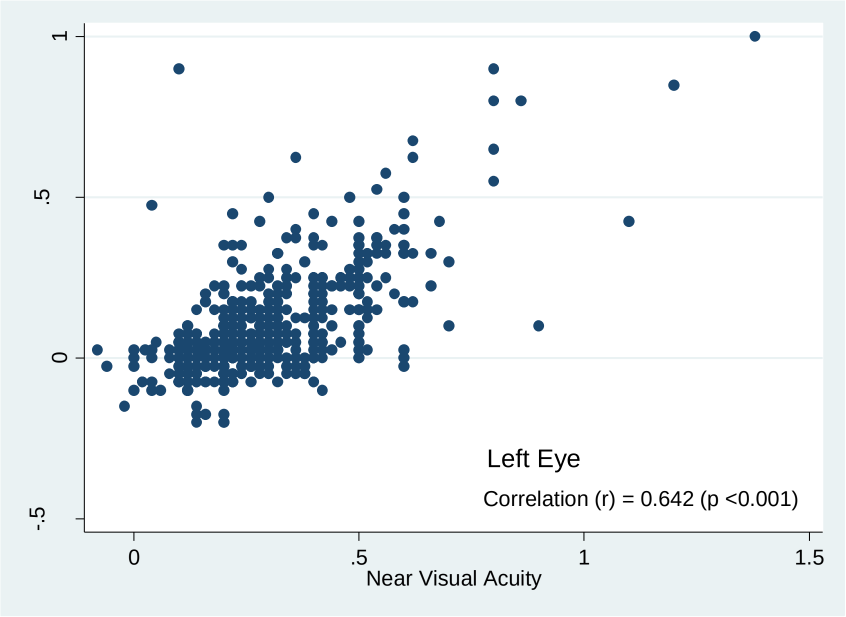

Supplement: Supplementary file 3 [file bmjopen-2017-021277supp003.pdf]
