# Supplementary material for: The effect of adherence to spectacle wear on early developing literacy: a longitudinal study based in a large multiethnic city, Bradford, UK
Source: BMJ Open. 2018 Jun 12;8(6):e021277. doi: 10.1136/bmjopen-2017-021277 (PMC6009541; doi:10.1136/bmjopen-2017-021277)
Supplement: Supplementary file 4 [file bmjopen-2017-021277supp004.pdf]

Supplementary Information 4.

Association between visual acuity (distance) and literacy, and between visual acuity (near) and literacy.

	Correlation with Letter ID standardised score at T3	
	**r**	**p-value**
Visual Acuity (far) - Best eye	-0.145	< 0.001
Visual Acuity (far) - Worst eye	-0.183	< 0.001
Visual Acuity (near) – Best eye	-0.115	0.006
Visual Acuity (near) - Worst eye	-0.140	< 0.001
