# Supplementary material for: The effect of adherence to spectacle wear on early developing literacy: a longitudinal study based in a large multiethnic city, Bradford, UK
Source: BMJ Open. 2018 Jun 12;8(6):e021277. doi: 10.1136/bmjopen-2017-021277 (PMC6009541; doi:10.1136/bmjopen-2017-021277)
Supplement: Author's manuscript [file bmjopen-2017-021277.draft_revisions.pdf]

BMJ Open

BMJ Open is committed to open peer review. As part of this commitment we make the peer review history of every article we publish publicly available.

When an article is published we post the peer reviewers' comments and the authors' responses online. We also post the versions of the paper that were used during peer review. These are the versions that the peer review comments apply to.

The versions of the paper that follow are the versions that were submitted during the peer review process. They are not the versions of record or the final published versions. They should not be cited or distributed as the published version of this manuscript.

BMJ Open is an open access journal and the full, final, typeset and author-corrected version of record of the manuscript is available on our site with no access controls, subscription charges or pay-per-view fees (http://bmjopen.bmj.com).

If you have any questions on BMJ Open's open peer review process please email info.bmjopen@bmj.com

BMJ Open

Effect of adherence to spectacle wear on early developing literacy: A longitudinal study.

Journal:	*BMJ Open*
Manuscript ID	bmjopen-2017-021277
Article Type:	Research
Date Submitted by the Author:	19-Dec-2017

Complete List of Authors:	Bruce, Alison; Bradford Institute for Health Research<br>Kelly, Brian; Bradford Institute for Health Research<br>Chambers, Bette; University of York, Institute for Effective Education<br>Barrett, Brendan; University of Bradford, School of Optometry and Vision Science<br>Bloj, Marina; University of Bradford, School of Optometry and Vision Science<br>Bradbury, John; Bradford Teaching Hospitals NHS Foundation Trust<br>Sheldon, Trevor; University of York, health Sciences

Keywords:	visual acuity, vision screening, literacy, spectacles, adherence

SCHOLARONE™
Manuscripts

For peer review only

Effect of adherence to spectacle wear on early developing literacy: A longitudinal study.

Alison Bruce (corresponding author), Post-doctoral Research Fellow and Head Orthoptist
Bradford Institute for Health Research, Bradford Teaching Hospitals NHS Trust, Duckworth Lane, Bradford, UK BD9 6RJ
01274 383414
alison.bruce@bthft.nhs.uk

Brian Kelly, Statistician
Bradford Institute for Health Research, Bradford Teaching Hospitals NHS Trust, Duckworth Lane, Bradford, UK BD9 6RJ
01274 383466
brian.kelly@bthft.nhs.uk

Bette Chambers, Professor and Director of Institute for Effective Education
University of York, Heslington, York, UK YO10 5DD
bette.chambers@york.ac.uk

Brendan T Barrett, Professor of Visual Development
School of Optometry and Vision Science, University of Bradford, Richmond Rd, Bradford, UK BD7 1DP
01274 235589
B.T.Barrett@bradford.ac.uk

Marina Bloj, Professor of Visual Perception
School of Optometry and Vision Science, University of Bradford, Richmond Rd, Bradford, UK BD7 1DP
01274 236258
M.Bloj@bradford.ac.uk

John Bradbury, Consultant Paediatric Ophthalmologist
Bradford Teaching Hospitals Foundation Trust, Duckworth Lane, Bradford, UK BD9 6RJ
john.bradbury@bthft.nhs.uk

Trevor A Sheldon, Professor of Health Services Research and Policy
Department of Health Sciences, University of York, Heslington, York, UK YO10 5DD
01904 321521
Trevor.Sheldon@york.ac.uk

Word count: 4178

For peer review only - http://bmjopen.bmj.com/site/about/guidelines.xhtml

ABSTRACT

Objectives: To determine the impact of adherence to spectacle wear on visual acuity and developing literacy following vision screening at age 4-5 years.

Design: Longitudinal study nested within the Born in Bradford birth cohort.

Setting and participants: Observation of 944 children; 432 had failed vision screening and were referred (treatment group) and 512 randomly selected (comparison group) who had passed (<0.20 logMAR in both eyes). Spectacle wear was observed in school for two years following screening and classified as adherent, (wearing spectacles at each assessment), or non-adherent.

Main outcome measures: Annual measures of visual acuity (VA) using a crowded logMAR Test. Literacy was measured by Woodcock Reading Mastery Tests-Revised subtest: letter identification.

Results: The VA of all groups improved over time. The VA of the adherent group (worse eye) improved significantly more than the comparison group, -0.008 log units per month (95% CI: -0.009 to -0.007), and by an additional -0.004 log units per month (95% CI: -0.005 to -0.003) in the better eye. The non-adherent group (worse eye) improved more than the comparison group by -0.003 log units per month (95% CI: -0.004 to -0.001) with no additional improvement in the better eye.

Literacy was associated with the VA, letter-ID reduced by -0.9 (95% CI:-1.15 to -0.64) for every one line (0.10 logMAR) fall in VA (better eye). This association remained after adjustment for socioeconomic and demographic factors (-0.360, 95% CI:-0.57 to -0.149). The adherent group consistently demonstrated higher letter-ID scores compared to the non-adherent group, with the greatest effect size (0.11) in Year 3.

For peer review only - http://bmjopen.bmj.com/site/about/guidelines.xhtml

Conclusions: Early literacy is associated with the level of VA; children who adhere to spectacle wear improve their VA and also have the potential to improve literacy. Our results suggest failure to adhere to spectacle wear has implications for the child's vision and education.

For peer review only

For peer review only - http://bmjopen.bmj.com/site/about/guidelines.xhtml

Strengths and limitations of this study

- This is the first longitudinal study to compare the effects of adherence and non-adherence to spectacles in children following vision screening at age 4-5 years on both visual acuity and developing literacy.
- Nesting the study within the Born in Bradford birth cohort allows adjustment for confounding factors.
- The study is observational in nature reflecting real life adherence to spectacle wear.
- The study is not a randomised controlled trial therefore allocation to the adherent or non-adherent groups is not exact and may underestimate the effect of non-adherence.

For peer review only

For peer review only - http://bmjopen.bmj.com/site/about/guidelines.xhtml

INTRODUCTION

Visual development in humans occurs in early-life[1] with the presence of reduced visual acuity (VA) in young children potentially indicating an associated condition such as significant refractive error, strabismus and/or amblyopia.[2] Visual impairment from amblyopia can potentially be lifelong, and profound and with a prevalence of 4% is an important public health issue.[3] The UK National Screening Committee (UK NSC) recommends visual screening for all children at age 4-5 years,[4] that is in the first year of school, in order to detect and treat early. For those who fail their test (>0.20 logMAR in one or both eyes)[4] the follow-up clinical pathway includes referral for a cycloplegic refraction and fundus examination to determine the presence and magnitude of any refractive error and to rule out eye disease.[3] In those with reduced VA, treatment generally consists of the wearing of spectacles[5] and may be combined with occlusion therapy[6] (wearing an eye patch or atropine drops). However, adherence to treatment, both spectacle wear[7,8] and occlusion therapy is known to be variable.[9]

Decreased VA is associated with reduced literacy levels in young children[10] and there is evidence that the presence of amblyopia affects reading ability.[11] However, there is a paucity of evidence on the impact of non-adherence to spectacle wear on VA and early developing literacy in children. Early literacy skills such as letter recognition,[12] word reading and decoding[13] taught in the first years of school are indicators of future reading performance and educational attainment, which in turn affect long-term health and social outcomes.[14,15] The initial school years are a crucial time for the development of these key literacy skills[16] and it is important to understand the impact of non-adherence to spectacle wear on visual outcome and educational attainment.

Low educational attainment is associated with socioeconomic deprivation,[15] which makes the investigation of the relationship between visual acuity and literacy difficult, as in order to account for potential confounding factors, comprehensive epidemiological data are required. Born in Bradford (BiB) is a large birth cohort, which collected maternal and early-life measures from mothers and their children in Bradford and details of recruitment have been

For peer review only - http://bmjopen.bmj.com/site/about/guidelines.xhtml

previously reported.[17] By linking separately-collected vision and literacy data in children in the BiB cohort, we had the opportunity to explore the association between VA, spectacle wear and literacy development whilst taking into account the effects of potential confounders. The aim of this study is to examine the impact of adherence to spectacle wear on early developing VA and literacy skills in children during their first three years of school.

METHODS

This is a prospective, longitudinal study nested within the BiB cohort following children from the point of their initial vision screening at age 4 -5 years. The study took place between 2012 and 2015. Baseline epidemiological data collected from mothers and children of the BiB cohort, literacy measures, vision screening results and repeat measures of vision and literacy were linked in order to investigate the longitudinal impact of adherence to spectacle wear on VA and early literacy.

Population

All children invited to join the study were participating in the BIB,[17] a longitudinal, multi-ethnic birth cohort study aiming to examine the impact of environmental, psychological and genetic factors on maternal and child health and wellbeing. Bradford is an ethnically diverse city (approximately half of the births are to mothers of South Asian origin) with high levels of socio-economic deprivation. The cohort is broadly representative of the city's maternal population.

Recruitment

As part of the separate BiB "Starting Schools Programme", children's literacy levels on school entry (termed 'Reception Class' in England, UK and defined as Year 1 of this study) were measured between September 2012 and July 2014. Seventy-four of the one hundred and twenty-three Bradford primary schools (60%) participated in "Starting Schools Programme" and these schools were also invited to join the vision and literacy study. Of the

For peer review only - http://bmjopen.bmj.com/site/about/guidelines.xhtml

2930 BiB children (74 schools), 432 (14.7%) had failed vision screening (Figure 1). These children were referred for follow-up cycloplegic investigation and are defined as the treatment group. A further 512 children from the same schools (randomly selected using Excel's random number generator) who had passed vision screening were also invited to participate and were defined as the comparison group, giving a total of 944 participants in the study. Consent was opt-out and parents received a letter via the schools requesting continued participation prior to each annual assessment. 893 of the 944 (94.6%) consented to participate in Year 2 and 650/944 (68.9%) participated in Year 3 (Figure 1).

Baseline Vision Assessments – Year 1

The vision screening programme for 4-5 year old children in Bradford is conducted in the first year of school by orthoptists with 97% of eligible children being screened.[18] The screening includes standard protocols for measurement of monocular distance VA.[19 ,20] VA was measured at a distance of three metres using the LogMAR Crowded Test (Keeler, Windsor, UK) which has four letters per line, with each letter having a score of 0.025; the total score for each line thus represents 0.10 log unit. A matching card was used and knowledge of letters was not therefore necessary to perform the test. VA was measured to threshold (i.e. best achievable VA with no defined endpoint). In addition cover test, ocular motility and non-cycloplegic auto refraction (Welch-Allyn Inc. Skaneateles. NY) were performed. The data formed the baseline vision data (Year 1). No child in the study was wearing spectacles at the baseline assessment.

Children failing to achieve the VA pass criterion set by the UK NSC[4] or who had a strabismus detected on cover testing were referred for follow-up, either to a community optometrist or the hospital eye service where a cycloplegic refraction (1% cyclopentolate hydrochloride) and fundus examination were undertaken, either by a paediatric ophthalmologist or an optometrist. Spectacles were prescribed based on the result of the cycloplegic refraction and clinical judgement. A follow-up appointment was then arranged with the orthoptist approximately 8 weeks after the cycloplegic examination to repeat the VA

For peer review only - http://bmjopen.bmj.com/site/about/guidelines.xhtml

measurement, with the child wearing spectacles if they had been prescribed. Children assessed by a community optometrist of their choice had the results of their examination returned to the hospital eye service and also had a follow-up appointment arranged with an orthoptist.

All VA testing, both at the point of vision screening and at follow-up, was performed using the same method of measurement. The results of the follow-up assessment including cycloplegic refraction, VA with the prescribed glasses, cover testing and fundus and media examination were extracted from the medical notes. The ophthalmic staff did not have knowledge of the baseline literacy assessment.

Baseline Literacy Assessments – Year 1

As part of the BiB “Starting Schools Programme”, literacy was measured on school entry (Year 1) by trained research assistants within the same academic term as the vision screening. The research assistants were unaware of the VA results. An age-appropriate literacy measure, the Woodcock Reading Mastery Tests-Revised (WRMT-R) subtest: Letter Identification (ID), a validated reading skill test, was used to assess early literacy.[21] Letter identification measures the child’s ability to identify single letters, an essential skill mastered prior to reading and one of the best predictors of future reading achievement.[14] The letter-ID test is a test of knowledge of letters (the complete alphabet is used) and the child must verbally identify the name of each letter. This literacy measure specifically uses varied font type; the size of the letters approximate to 1.1 log unit (20/250) at 33cm, therefore the performance on this test is not affected by the level of VA. Letter-ID was collected in both raw and age standardised format. In addition receptive vocabulary, an indicator of language ability, was measured using the British Picture Vocabulary Scale (BPVS),[22] providing a representation of early language ability and cognition.

For peer review only - http://bmjopen.bmj.com/site/about/guidelines.xhtml

Follow up Assessments - Years 2 and 3

Vision and literacy measures were repeated within the same school term approximately 12 months (Year 2) and 24 months (Year 3) after the baseline measurements. Both the vision and the literacy assessments were administered on the same day by the same personnel who were unaware of the previous year's vision or literacy results. VA and literacy was measured as detailed above. VA found to be ≥0.10 logMAR was repeated with a pinhole and near VA was measured using the Bailey-Lovie near-vision chart.[23] Cover test, ocular motility, non-cycloplegic auto refraction (Welch-Allyn Inc. Skaneateles. NY) and whether the child was wearing spectacles were recorded. In order to present the real-life impact of adherence to spectacle wear, all VA measures reported are presenting visual acuities i.e. measured with spectacles if worn at the time of the assessment in school. Parents and children were not given prior warning of these assessments. This study was approved by National Research Ethics Committee Yorkshire & the Humber- South Yorkshire (Ref 13/YH/0379).

Statistical Analysis

Children with baseline data for both vision and literacy in Year 1 and who had at least one follow-up measure in either Year 2 or Year 3 were included in the final analysis (Figure 1). The characteristics of children participating in the study were compared initially using Chi-squared or two sided t-tests as appropriate. Children in the treatment group were retrospectively divided into two sub-groups, adherent and non-adherent. Adherence was defined as wearing prescribed spectacles at the time of assessment; otherwise children were defined as non-adherent. Children who were assessed twice but only wore the spectacles on one occasion were classed as non-adherent.

Analysis of Visual Acuity

To investigate the effect of spectacle wear over time on VA, multilevel longitudinal models[24] were firstly constructed with VA as the outcome measure for the child's better and worse eye. The models measure change within the individual and change between individuals over

For peer review only - http://bmjopen.bmj.com/site/about/guidelines.xhtml

time and allow for individual differences in the rate of change over time.[24] A quadratic term was included to model the non-linear trajectory of change. The model also includes an interaction term to compare the relationship between age and group, to test whether differences by group are the same at different ages. Unadjusted analysis was initially undertaken with subsequent adjustment for demographic and socioeconomic factors reported in the literature to be associated with reduced VA: early-life factors[25] (gender, gestational age, birth weight, route of birth) and maternal factors[26] (ethnicity, mother's age at delivery, mother's level of educational attainment and being in receipt of means-tested benefits). Predicted outcomes were plotted to visualise group differences and change in the outcomes for each group over time.

Analysis of Literacy

In order to estimate the association between the letter-ID and VA the same multilevel and longitudinal modelling approach was adopted, but with the final letter-ID score as the outcome measure. The raw letter-ID scores were used in the analysis in order to explore change over time. After estimating differences between the groups and accounting for the initial letter-ID at baseline (Year 1), further adjustment was undertaken for the factors reported in the literature to be associated with educational attainment, the early-life factors[27] and maternal factors as previously stated.[28] Spherical equivalent refraction (SER) (sphere plus half cylinder) of the better eye was included as was BPVS score in order to account for language ability. The results of these models are presented along with predicted outcomes for each of the groups. Effect sizes are generally reported when appraising educational interventions. To demonstrate group differences at each time point effect sizes were calculated for the letter-ID scores using Cohen's *d*.[29] All analyses were carried out using Stata V.13 (StataCorp, College Station, Texas, USA).

For peer review only - http://bmjopen.bmj.com/site/about/guidelines.xhtml

RESULTS

Twelve children in the treatment group were excluded from the analysis as they had ocular conditions other than refractive error (e.g. nystagmus) confirmed in their medical notes, leaving 368 children in the treatment group and 433 in the comparison group. Data from 801 (85%) children from 67 schools were included in the final analysis (Figure 1). 230/368 (62.5%) of children in the treatment group had attended for the initial cycloplegic examination and been prescribed spectacles, 3/368 (0.8%) attended but no cycloplegic refraction information was available, 23/368 (6.3%) had been prescribed spectacles but had not returned for follow-up VA assessment, and 112/368 (30.4%) had failed to attend any appointment following vision screening. Of the 253 children in the treatment group with cycloplegic refraction results, 157/253 (62.1%) had astigmatism (>1.00DC) either alone or in combination with hypermetropia or myopia. 35/253 (13.8%) had hypermetropia (>+3.0DS) alone, 11 (4.3%) had myopia (≤-0.50DS) alone and 50 (19.8%) children had low hypermetropia (>+1.0DS to +3.0DS). 55 of 253 (21.7%) additionally had anisometropia (≥1.0D difference). For those children with a cycloplegic refraction result (Table 1) the SER ranged from -7.875 to +7.50D in the better eye and -8.25 to +7.50D in the worse eye. Fourteen of the 368 (3.8%) children had a constant or intermittent strabismus, seven of whom had been prescribed occlusion therapy. Those children were not excluded from the analysis as they met the initial VA referral criteria and had been prescribed spectacles. Baseline (Year 1) characteristics of the children in the comparison and treatment groups are shown in Table 1. A small mean difference (-0.021 logMAR, 95% CI -0.022 to -0.020) in VA between the eyes of the comparison group was found, equating to one letter difference. This is not clinically significant but is statistically significant therefore VA's are presented for the better and worse eye separately. Higher levels of VA were found in both eyes of the comparison group compared to the treatment group (Chi-squared p<0.001) (Table 1). The only demographic factor found to differ between the comparison and the treatment group was the average mother's age which was around 10 months more in the treatment group (Chi-squared p<0.001).

For peer review only - http://bmjopen.bmj.com/site/about/guidelines.xhtml

Table 1. Characteristics of Born in Bradford children and mothers included in the analyses. Values are numbers (%) or mean (SD).

	Comparison group n=433	Treatment group n=368	P value†
**Children**			
Age (months) Year 1	60 (4.2)	60 (4.5)	0.119
Gender			
Male	229 (51.1)	183 (49.7)	
Female	219 (48.9)	185 (50.3)	0.693
Ethnicity			
White	125 (28.0)	91 (24.9)	
Pakistani	262 (58.7)	232 (63.4)	
Other	59 (13.3)	43 (11.7)	0.403
Route of birth			
Vaginal	342 (77.0)	291 (79.7)	
Caesarean	102 (23.0)	74 (20.3)	0.355
Gestational age at birth (weeks)	277 (12.0)	276 (13.0)	0.158
Birth weight (g)	3184 (550.0)	3128 (573.0)	0.155
VA better eye	0.113 (0.049)	0.271 (0.138)	<0.001
VA worse eye	0.135 (0.046)	0.428 (0.189)	<0.001
SER better eye ‡	-	1.19 (0.95)	-
SER worse eye ‡	-	1.98 (1.27)	-
**Mother**			
Age (years)	27.3 (5.4)	28.1 (5.7)	<0.001
Mother's education			
<A-level	227 (64.5)	190 (69.3)	
A-level or above	125 (35.5)	84 (31.7)	0.201
In receipt of means tested benefits (yes)	163 (45.0)	144 (50.1)	0.139

†Difference between Comparison and treatment groups (chi-squared or t-test as appropriate).
VA = visual acuity. VA's are measured in logMAR; therefore higher values represent poorer VA.
SER= spherical equivalent refraction. ‡Cycloplegic results were available for the treatment group only.

Table 2 presents the baseline (Year 1) characteristics of those children in the treatment group retrospectively categorised as adherent (173/368, 47.0%) and non-adherent (195/368, 53.0%) (Figure1). In the non-adherent group, no child wore spectacles at their Year 2 assessment and 39/195 (20%) wore them in Year 3 only. At baseline, the group subsequently classed as adherent had a lower level of VA compared to the non-adherent group in both the better and worse eye (Table 2). The only other factor that differed between the adherent and the non-adherent groups was the mother's level of education with 50/173 (39.1%) of adherent children having mothers educated to A-level or above compared to only 34/195 (23.3%) of the non-adherent group (Chi-squared p=0.005). Language ability (BPVS) did not differ between the adherent and non-adherent children (p=0.553), suggesting that there were no differences in cognitive ability.

For peer review only - http://bmjopen.bmj.com/site/about/guidelines.xhtml

Table 2. Baseline characteristics of participants in the treatment group retrospectively classed as adherent and non-adherent. Values are numbers (%) or mean (SD).

	Adherent n=173 (47.0%)	Non-adherent n=195 (53.0%)	P value†
**Children**			
Age (months) Year 1	59.4 (4.5)	59.6 (4.5)	0.850
Gender			
Male	81 (46.8)	102 (52.3)	
Female	92 (53.2)	93 (47.7)	0.293
Ethnicity			
White	48 (27.9)	43 (22.2)	
Pakistani	103 (59.9)	129 (66.5)	
Other	21 (12.2)	22 (11.3)	0.387
Route of birth			
Vaginal	137 (79.6)	154 (79.8)	
Caesarean	35 (20.4)	39 (20.2)	0.973
Gestational age at birth (weeks)	276 (13.0)	275 (14.0)	0.383
Birth weight (g)	3121 (569.0)	3134 (579.0)	0.833
VA better eye‡	0.292 (0.150)	0.256 (0.129)	0.008
VA worse eye‡	0.465 (0.197)	0.399 (0.175)	0.001
SER better eye	1.18 (0.86)	1.20 (1.02)	0.960
SER worse eye	2.02 (1.20)	1.96 (1.33)	0.657
Language ability scores§	97.8 (15.6)	96.8 (16.4)	0.553
**Mother**			
Age (years)	28.1 (5.8)	28.0 (5.7)	0.845
Mother's education			
<A-level	78 (60.9)	112 (76.7)	
A-level or above	50 (39.1)	34 (23.3)	0.005
In receipt of means tested benefits (yes)	61 (45.5)	83 (55.7)	0.087

†Difference between Adherent and non-adherent treatment groups (chi-squared or t-test as appropriate).
VA = visual acuity. VA's are measured in logMAR; therefore higher values represent poorer VA.
SER= spherical equivalent refraction. §Age-adjusted language ability measure for British Picture Vocabulary Score (BPVS).
‡No child was wearing spectacles at the baseline assessment.

Visual Acuity

At baseline, both the adherent (mean diff: 0.337 logMAR; 95% CI: 0.304 to 0.370) and non-adherent groups (mean diff: 0.273 logMAR; 95% CI: 0.241 to 0.305) had lower levels of VA in the worse eye compared to the comparison group. Table 3 and Figure 2 present the VA trajectories over the course of the study. These show that after adjusting for previously described early-life and maternal variables, the VA of both eyes for all three groups; the comparison, the adherent and the non-adherent groups improve over time. The VA of the worse eye in the comparison group demonstrates improvement over time of -0.009 (95% CI -0.011 to -0.007) log units per month (approximately one letter every 3 months). The VA of the worse eye in the adherent group improved over and above the comparison group by -

For peer review only - http://bmjopen.bmj.com/site/about/guidelines.xhtml

0.008 log units per month (95% CI: -0.009 to-0.007) (approximately two letters every 3 months) and also demonstrated a small amount of improvement (-0.004 log units per month; 95%CI: -0.005 to -0.003) over and above the comparison group in the better eye (Table 3). The non-adherent group showed a small improvement over and above the comparison group (-0.003 log units per month; 95%CI: -0.004 to -0.001) in the worse eye but no additional improvement in the better eye (Table 3).

For peer review only

For peer review only - http://bmjopen.bmj.com/site/about/guidelines.xhtml

Table 3. Change in visual acuity for the better and worse eye over time by group; comparison, adherent and non-adherent.

	**Unadjusted (worse eye) (95% CI)**	**Adjusted‡ (worse eye) (95% CI)**	**Unadjusted (better eye) (95% CI)**	**Adjusted‡ (better eye) (95% CI)**
Constant	0.177 (0.159 to 0.194)*	0.386 (0.124 to 0.648)*	0.240 (0.026 to 0.454)*	0.240 (0.026 to 0.454)*
Age (months)	-0.009 (-0.011 to -0.008)***	-0.009 (-0.011 to -0.007)***	-0.006 (-0.008 to -0.005)***	-0.006 (-0.008 to -0.005)***
Age (months) squared	0.00016 (0.00012 to 0.00020)***	0.00016 (0.00012 to 0.00021)***	0.00010 (0.00007 to 0.00013)***	0.00010 (0.00006 to 0.00014)***
Group (reference: comparison)				
Adherent	0.337 (0.309 to 0.366)***	0.337 (0.304 to 0.370)***	0.184 (0.162 to 0.106)***	0.170 (0.144 to 0.196)***
Non-adherent	0.277 (0.250 to 0.305)***	0.273 (0.241 to 0.305)***	0.150 (0.128 to 0.172)***	0.148 (0.123 to 0.174)***
Age [X] Group interaction				
Age [X] adherent	-0.008 (-0.009 to -0.007)***	-0.008 (-0.009 to -0.007)***	-0.004 (-0.005 to -0.004)***	-0.004 (-0.005 to -0.003)***
Age [X] non-adherent	-0.003 (-0.003 to -0.001)***	-0.003 (-0.004 to -0.001)***	-0.001 (-0.002 to 0.000)	-0.002 (-0.004 to 0.000)

‡Model adjusted for gender, ethnicity, gestation period, birthweight, birth route, maternal education status, maternal age and means-tested benefit status.
[X] Interaction between group and age to determine if the effect of being in a particular group changes with age. The total effect for any one group is the coefficient for age **plus** the additional effect of age for that group.
*p < 0.05, **p < 0.01, ***p< 0.001

For peer review only

For peer review only - http://bmjopen.bmj.com/site/about/guidelines.xhtml

Table 4. Associations between Letter-ID score, visual acuity (better eye), maternal and early-life factors.

FACTOR	UNADJUSTED MODEL (95% CI)	p value	FULLY ADJUSTED MODEL (95% CI)	p value
Constant	18.82 (17.91 to 19.73)	<0.001	-20.6 (-28.2 to -13.0)	<0.001
Age	1.30 (1.21 to 1.38)	<0.001	1.28 (1.19 to 1.37)	<0.001
Age squared	-0.02 (-0.02 to -0.02)	<0.001	-0.020 (-0.022 to -0.017)	<0.001
Visual Acuity: change in Letter-ID per 0.1log unit (one line)	-0.90 (-1.15 to -0.64)	<0.001	-0.327 (-0.540 to -0.115)	0.003
Letter ID baseline (Year 1)			0.348 (0.326 to 0.371)	<0.001
BPVS			0.019 (-0.001 to 0.039)	0.064
Ethnicity				
Pakistani heritage			0.668 (-0.016 to 1.353)	0.056
Other			1.174 (1.159 to 2.189)	0.023
Gender				
Female			0.471 (-0.093 to 1.035)	0.102
Birth weight (per 100g)			0.074 (0.008 to 0.141)	0.029
Gestational age (weeks)			-0.053 (-0.257 to 0.151)	0.611
Receiving Benefits			-0.086 (-0.661 to 0.4990)	0.770
Mothers Level of Education	(higher than A-level)		0.765 (0.156 to 1.374)	0.014
Mothers age at birth (years)			-0.048 (-0.100 to 0.005)	0.075

BPVS = British Picture Vocabulary Scale (baseline standardised score).

For peer review only

Literacy

The unadjusted model shows the final letter-ID score reduces by -0.9 units (95% CI:-1.15 to -0.64) for every one line (0.10 logMAR) fall in VA of the better eye (Table 4). This association persists but is weaker after fully adjusting for the socioeconomic and demographic factors, with letter-ID score reducing by -0.327 units (95% CI:-0.540 to -0.115) for every one line fall in VA. Separate adjusted analysis of the VA level of the worse eye shows similar results but with weaker association, letter-ID score reduces by -0.260 units (95% CI:-0.414 to -0.105) for every one line fall in VA.

Children of mothers educated to A-level or above had increased letter-ID scores (0.765 units; 95% CI: 0.156 to 1.374) compared to those with lower qualifications. Ethnicity other than white British or Pakistani heritage was associated with better letter-ID score, which might reflect the higher number of mothers educated to above A-level in this group. Greater birth weight was also associated with increased letter-ID score (Table 4). Adjustment for SER made no difference and was not associated with letter-ID (p=0.306). It was therefore not included in the models.

For peer review only - http://bmjopen.bmj.com/site/about/guidelines.xhtml

A predictive model of the letter-ID score over time for children in each group (Figure 3) was constructed using both the unadjusted and adjusted data from the VA trajectories (Table 3) and incorporated into the model reporting letter-ID (Table 4). The unadjusted trajectory shows both adherent and non-adherent groups at baseline have lower letter-ID scores than the comparison group. The predicted trajectory of improvement in the adherent group is greater than the non-adherent group with the later letter-ID scores of the adherent group converging on those of the comparison group by Year 3. The non-adherent group although improving over time does not catch up with the adherent or the comparison groups. After adjusting for socio-economic and demographic variables the trend is similar but with a smaller difference between the groups.

Table 5 presents the effect size of wearing spectacles on the letter-ID scores between the groups annually over the three years of the study. Comparing the letter-ID scores between the adherent and the non-adherent group a gradual increase in the effect size over time is demonstrated with the greatest effect size (0.11) between the adherent and non-adherent groups shown in Year 3.

For peer review only

For peer review only - http://bmjopen.bmj.com/site/about/guidelines.xhtml

Table 5. Annual Literacy Scores by Group.

Year	Group	Letter-ID (raw score)	Comparison Groups	Effect Size (Cohen's *d*)*
1	Comparison	25	Comparison v Adherent	0.06
	Adherent	24.3	Comparison v Non-adherent	0.06
	Non-adherent	24.3	Adherent v Non-Adherent	0.00†
2	Comparison	34.7	Comparison v Adherent	0.05
	Adherent	34.4	Comparison v Non-adherent	0.13
	Non-adherent	34.0	Adherent v Non-Adherent	0.07
3	Comparison	39.1	Comparison v Adherent	0.08
	Adherent	38.8	Comparison v Non-adherent	0.18
	Non-adherent	38.4	Adherent v Non-Adherent	0.11

*Based on group difference divided by the pooled standard deviation (SD) of Letter-ID score: SD 10.9 at Year 1, 5.6 at Year 2 and 3.8 at Year 3.
† In Year 1 there is no difference as spectacle wear has not commenced.

DISCUSSION

This study is the first to assess VA and literacy in children following vision screening. Our results indicate that early developing literacy is affected by the level of VA even after adjusting for socio-economic and demographic factors associated with educational attainment. The letter-ID score reduces by approximately 1.5% for every one line of reduction in VA. In this and similar populations [13 ,30,] where children have been reported to have reduced VA levels (>0.30logMAR in better eye), there is likely to be an impact on developing literacy skills. The effect size (0.11) of being adherent to spectacle wear compared to non-adherence in Year 3 of our study is the same as that reported in a Chinese study providing free spectacles to children[31] and is comparable with reported educational interventions.[32] Thus children who fail vision screening and adhere to spectacle wear have the potential to improve their VA, further influencing early literacy development.

Adherence to spectacle wear is highly influenced by socio-economic and demographic factors, particularly maternal education, a factor that is also known to be associated with educational attainment.[33] Children with reduced VA and who are in less educated families are less likely to adhere to treatment, which will further impact on their educational

For peer review only - http://bmjopen.bmj.com/site/about/guidelines.xhtml

attainment and future life chances. We were however, able to adjust for the many associated maternal and early-years factors, the value of embedding this study within a birth cohort. A small number of cross-sectional studies[34,35] have examined the relationship between failing vision screening and academic performance, using a variety of visual performance measures. A study examining academic performance in US schools reports that failing vision screening was predictive of being in the lowest quartile of academic performance.[35] Reduced reading performance in kindergarten has been reported in children failing a modified vision assessment test using the addition of +/- 2.00D lenses compared to those passing.[34] Conversely, a longitudinal study of children aged 9 – 10 years in Singapore, Dirani et al[36] found VA did not play a significant role in predicting academic performance. The children were however older, mainly myopic and only a small number of participants had decreased VA which may account for the difference in their findings relative to ours.

The VA of children in all groups (adherent, non-adherent and comparison group) continued to improve throughout this study. The improvement in VA found in the comparison group is similar to that reported for normal visual development, with optimum VA achieved around 6 years of age.[37,38] The improvement in VA of the worse eye found in adherent children over the time of the study was significantly greater than that expected solely from visual development[39] or indeed from retest variability[40] and was almost double that of the comparison group. Little additional improvement above that expected from visual development was demonstrated in the worse eye of the non-adherent children, an indication that the improvement in the adherent children is not due to regression to the mean. The longitudinal observation of the children demonstrates improvement not only in VA but also in literacy, with the non-adherent group demonstrating persistently lower literacy scores throughout the study, although the effect is attenuated after adjusting for other factors.

Annual improvement in academic achievement is well recognised and is particularly notable in the early years of schooling with the initial improvement thought to be associated with the effect of entering school, combined with rapid early child development followed by a plateau in academic growth as children progress through school grades.[19]

For peer review only - http://bmjopen.bmj.com/site/about/guidelines.xhtml

Early literacy development is complex and associated with socio-economic and demographic factors, in particular maternal education. However, even after taking these factors into account VA continues to be associated with literacy; the poorer the level of VA, the greater the reduction in the literacy score. In a Singaporean study,[39] a strong association between paternal level of education and academic school performance was reported and a Chinese study reporting improved mathematics performance with the provision of free glasses also indicated that parental education was highly associated with educational outcome.[31] As one might expect, higher levels of maternal education have a positive impact on literacy. [41,42] In addition, mothers with higher educational attainment are more likely to effectively access health services, and are more likely to adhere to prescribed treatment.[43]

We found no association between SER and literacy score. This differs from previous studies reporting an association between refractive error and literacy.[44,45] Hypermetropia has been reported to be associated with poor literacy although one study was a pilot and had a very small sample size.[44] Another study reports an association between hypermetropia and literacy levels in those children failing vision screening, but not those who passed, potentially biasing the results.[45] A large American study of pre-school children aged 4-5 years found that children with hypermetropia and reduced near VA have poorer print knowledge than those with hypermetropia and a good level of near visual acuity.[46] Bilateral uncorrected astigmatism has also been reported to reduce reading fluency, and children with moderate astigmatism are reported to have lower VA and fluency than those with no or low astigmatism.[47] In our study a wide range of refractive errors is included ranging from moderate myopia to moderate hypermetropia, with the majority of children having astigmatism (>1.00DC) and this may account for the association found between VA and literacy but not between SER and literacy.

The longitudinal design of this study provides an insight into development of VA and literacy in the early years of schooling, and the use of linked data from the mothers and children participating in the BiB cohort study permitted the many potential confounding factors associated with educational attainment to be accounted for. The study does however have

For peer review only - http://bmjopen.bmj.com/site/about/guidelines.xhtml

some weaknesses. It is not a randomised controlled trial and non-adherence was defined retrospectively by the failure of the child to wear their prescribed glasses at one assessment; it is possible that this was a unique event and is not representative of the child's true adherence to spectacle wear over the course of the study. If this is indeed the case, then the random misclassification is likely to under-estimate the difference found between the adherent and non-adherent groups. Distance visual acuity is the sole measure of visual function included in the study and it is possible other measures of visual function are also associated with academic performance; further research would be required to explore these associations.

During visual maturation, the presence of neurodevelopmental disorders such as refractive error, and strabismus may contribute to a reduction in VA and early intervention is required. This study demonstrates that wearing spectacles is an effective intervention to improve VA, and that this will impact positively on developing literacy. The children who do not adhere to spectacle wear are likely to be those in families who are poorer and less educated. Further research is required to better understand the reasons for non-adherence and evaluate interventions to promote adherence to spectacle wear. This has the potential not only to improve vision but also support future life chances in children who may already face educational disadvantage.

For peer review only

For peer review only - http://bmjopen.bmj.com/site/about/guidelines.xhtml

REFERENCES

1. Daw NW. Critical periods and amblyopia. *Arch Ophthalmol* 1998;116(4):502-5.

2. Robaei D, Rose K, Ojaimi E*, et al.* Visual Acuity and the Causes of Visual Loss in a Population-Based Sample of 6-Year-Old Australian Children. *Ophthalmology* 2005;112(7):1275-82.

3. Public Health England. Child vision screening resources consultation. Available: https://www.gov.uk/government/consultations/child-vision-screening-resources (Accessed 04/08/2017). 2017.

4. National Screening Committee. The UK NSC policy on Vision defects screening in children 2013. Available: http://www.screening.nhs.uk/vision-child (Accessed 19/12/2017).

5. Stewart CE, Moseley MJ, Fielder AR*, et al.* Refractive adaptation in amblyopia: quantification of effect and implications for practice. *Br J Ophthalmol* 2004;88(12):1552-6.

6. Pediatric Eye Disease Investigator G, Repka MX, Kraker RT*, et al.* A randomized trial of atropine vs patching for treatment of moderate amblyopia: follow-up at age 10 years. *Arch Ophthalmol* 2008;126(8):1039-44.

7. Aldebasi YH. A descriptive study on compliance of spectacle-wear in children of primary schools at Qassim Province, Saudi Arabia. *Int J Health Sci (Qassim)* 2013;7(3):291-99.

8. Sharma A, Congdon N, Patel M*, et al.* School-based approaches to the correction of refractive error in children. *Surv Ophthalmol* 2012;57(3):272-83.

9. Maconachie GDE, Gottlob I. The challenges of amblyopia treatment. *Biomed J* 2015;38(6):510-16.

10. Bruce A, Fairley L, Chambers B*, et al.* Impact of visual acuity on developing literacy at age 4-5 years: a cohort-nested cross-sectional study. *BMJ Open* 2016;6(2):e010434.

11. Stifter E, Burggasser G, Hirmann E*, et al.* Monocular and binocular reading performance in children with microstrabismic amblyopia. *Br J Ophthalmol,* 2005;89(10):1324-9.

12. Foulin JN. Why is letter-name knowledge such a good predictor of learning to read? *Read Writ* 2005;18(2):129-55.

For peer review only - http://bmjopen.bmj.com/site/about/guidelines.xhtml

13. Marchman VA, Fernald A. Speed of word recognition and vocabulary knowledge in infancy predict cognitive and language outcomes in later childhood. *Dev Sci* 2008;11(3):9-16.

14. Dearden L, Sibieta L, Sylva K. The socio-economic gradient in early child outcomes: evidence from the Millennium Cohort Study. *Longit Life Course Stud* 2011;2(1):19 - 40.

15. Marmot M. Fair Society, Healthy Lives.The Marmot review Executive Summary. London: The Marmot Review 2010.

16. Bloom HS, Hill CJ, Black AR*, et al.* Performance Trajectories and Performance Gaps as Achievement Effect-Size Benchmarks for Educational Interventions. *J Res on Educ Eff* 2008;1(4):289-328.

17. Wright J, Small N, Raynor P*, et al.* Cohort Profile: The Born in Bradford multi-ethnic family cohort study. *Int J Epidemiol* 2013;42(4):978-91.

18. Bruce A, Outhwaite L. Uptake, referral and attendance: results from an inner city school based vision screening programme. *Br Ir Orthopt J* 2013;10:41 - 45.

19. McGraw PV, Winn B, Gray LS*, et al.* Improving the reliability of visual acuity measures in young children. *Ophthalmic Physiol Opt* 2000;20(3):173-84.

20. Simmers AJ, Gray LS, Spowart K. Screening for amblyopia: a comparison of paediatric letter tests. *Br J Ophthalmol* 1997;81(6):465-69.

21. Woodcock RW. Woodcock Reading Mastery Tests (Revised) Circle Pines, MN: American Guidance Service, 1987.

22. Dunn LM, Dunn LM, Whetton C*, et al.* British Picture Vocabulary Scale. 2nd edition Windsor, Berks: NFER-Nelson, 1997.

23. Bailey IL, Lovie JE. The design and use of a new near-vision chart. *Am J Optom Physiol Opt* 1980;57(6):378-87.

24. Goldstein H. Models for Repeated Measures Data. Multilevel Statistical Models: John Wiley & Sons, Ltd, 2010:147-60.

For peer review only - http://bmjopen.bmj.com/site/about/guidelines.xhtml

25. Williams C, Northstone K, Howard M, *et al.* Prevalence and risk factors for common vision problems in children: data from the ALSPAC study. *Br J Ophthalmol* 2008;92(7):959-64.

26. Tarczy-Hornoch K, Varma R, Cotter SA, *et al.* Joint Writing Committee for the Multi-Ethnic Pediatric Eye Disease Study the Baltimore Pediatric Eye Disease Study Groups. Risk Factors for Decreased Visual Acuity in Preschool Children: The Multi-Ethnic Pediatric Eye Disease and Baltimore Pediatric Eye Disease Studies. *Ophthalmology* 2011;118(11):2262-73.

27. Burroughs-Lange SG, Douetil J. Literacy progress of young children from poor urban settings: A Reading Recovery comparison study. *Literacy Teaching and Learning* 2007;12(1):19-46.

28. Schoon I, Parsons S, Sacker A, *et al.* Socioeconomic adversity, educational resilience and subsequent levels of adult adaptation. *J Adolesc Res* 2004;19:383-404.

29. Cohen J. *Statistical power analysis for the behavioral sciences (2nd ed). Mahwah, NJ*, 1988.

30. Murthy GV, Gupta SK, Ellwein LB, *et al.* Refractive error in children in an urban population in New Delhi. *Invest Ophthalmol Vis Sci* 2002;43.

31. Ma X, Zhou Z, Yi H, *et al.* Effect of providing free glasses on children's educational outcomes in China: cluster randomized controlled trial. *BMJ* 2014;349.

32. McEwan PJ. Improving Learning in Primary Schools of Developing Countries:A Meta-Analysis of Randomized Experiments. *Rev Educ Res* 2015;85(3):353-94.

33. Neuman S. Lessons from my mother: Reflections on the National Early Literacy Report. *Educ Res* 2010;39(4):301 -04.

34. Kulp MT, Schmidt PP. Visual predictors of reading performance in kindergarten and first grade children. *Optom Vis Sci* 1996;73(4):255-62.

35. Krumholtz I. Results from a pediatric vision screening and its ability to predict academic performance. *Optometry (St Louis, Mo)* 2000;71(7):426-30.

For peer review only - http://bmjopen.bmj.com/site/about/guidelines.xhtml

36. Dirani M, Zhang X, Goh LK*, et al.* The Role of Vision in Academic School Performance. *Ophthalmic Epidemiol* 2010;17(1):18-24.

37. Leone JF, Mitchell P, Kifley A*, et al.* Normative visual acuity in infants and preschool-aged children in Sydney. *Acta Ophthalmol* 2014;92(7):e521-e29.

38. Pan Y, Tarczy-Hornoch K, Susan A C*, et al.* Visual Acuity Norms in Preschool Children: The Multi-Ethnic Pediatric Eye Disease Study. *Optom Vis Sci* 2009;86(6):607-12.

39. Sonksen PM, Wade AM, Proffitt R*, et al.* The Sonksen logMAR test of visual acuity: II. Age norms from 2 years 9 months to 8 years. *J AAPOS* 2008;12(1):18-22.

40. Stewart C. Comparison of Snellen and log-based acuity scores for school-aged children. *Br Orthopt J* 2000;57:32-38.

41. Magnuson K. Maternal education and children's academic achievement during middle childhood. *Dev Psychol* 2007;43(6):1497-512.

42. Taylor J, Ennis CR, Hart SA*, et al.* Home environmental and behavioral risk indices for reading achievement. *Learn Individ Differ* 2017;57:9-21.

43. Leurer MD. Perceived Barriers to Program Participation Experienced by Disadvantaged Families. *Int J Health Promot Educ* 2011;49(2):53-59.

44. Shankar S, Evans MA, Bobier WR. Hyperopia and emergent literacy of young children: pilot study. *Optom Vis Sci* 2007;84(11):1031-8.

45. Williams WR, Latif AH, Hannington L*, et al.* Hyperopia and educational attainment in a primary school cohort. *Arch Dis Child* 2005;90(2):150-3.

46. VIP-HIP Study Group, Kulp MT, Ciner E*, et al.* Uncorrected Hyperopia and Preschool Early Literacy: Results of the Vision in Preschoolers-Hyperopia in Preschoolers (VIP-HIP) Study. *Ophthalmology* 2016;123(4):681-9.

47. Harvey EM, Miller JM, Twelker JD*, et al.* Reading Fluency in School-Aged Children with Bilateral Astigmatism. *Optom Vis Sci* 2016;93(2):118-25.

For peer review only

Funding: AB is funded by a National Institute for Health Research Post-Doctoral Fellowship Award (PDF-2013-06-050). The Born in Bradford study presents independent research commissioned by the National Institute for Health Research Collaboration for Applied Health Research and Care (NIHR CLAHRC) and the Programme Grants for Applied Research funding scheme (RP-PG-0407-10044). The views expressed are those of the author(s) and not necessarily those of the NHS, the NIHR or the Department of Health.

Competing interest statement: "All authors have completed the Unified Competing Interest form and declare: no support from any organisation for the submitted work; no financial relationships with any organisations that might have an interest in the submitted work in the previous three years, no other relationships or activities that could appear to have influenced the submitted work.

Acknowledgments: We thank all the families and schools who took part in this study, the orthoptists from Bradford Teaching Hospitals Foundation Trust who conducted the vision screening programme, the researchers from the Starting Schools programme who collected the literacy measures, Patrick Friis, Alexandra Morris and Hannah Farrugia who collected follow-up measures and the Data Support Team from Bradford Institute for Health Research who created and maintain the data linkage system.

Contributors: AB initiated the project, designed data collection, monitored data collection for the whole study, wrote the statistical analysis plan, cleaned and analysed the data, and drafted and revised the paper. She is guarantor. BK wrote the statistical analysis plan, cleaned the data and revised the draft paper. BC initiated the project and revised the draft paper. BTB contributed to the design of the study and revised the draft paper. MB contributed to the design of the study and revised the draft paper. JB contributed to the design of the study and revised the draft paper. TAS initiated the project, wrote the statistical analysis plan and revised the draft paper.

For peer review only - http://bmjopen.bmj.com/site/about/guidelines.xhtml

“The Corresponding Author has the right to grant on behalf of all authors and does grant on behalf of all authors, a worldwide licence to the Publishers and its licensees in perpetuity, in all forms, formats and media (whether known now or created in the future), to i) publish, reproduce, distribute, display and store the Contribution, ii) translate the Contribution into other languages, create adaptations, reprints, include within collections and create summaries, extracts and/or, abstracts of the Contribution, iii) create any other derivative work(s) based on the Contribution, iv) to exploit all subsidiary rights in the Contribution, v) the inclusion of electronic links from the Contribution to third party material where-ever it may be located; and, vi) licence any third party to do any or all of the above.”

"The Corresponding Author has the right to grant on behalf of all authors and does grant on behalf of all authors, an exclusive licence on a worldwide basis to the BMJ Publishing Group Ltd to permit this article (if accepted) to be published in BMJ editions and any other BMJPGL products and sublicences such use and exploit all subsidiary rights, as set out in our licence."

Transparency declaration: Dr Alison Bruce, lead author (the manuscript’s guarantor) affirms that the manuscript is an honest, accurate, and transparent account of the study being reported.

No additional data is available.

For peer review only

Figure Legends:

Figure1. Flow chart of the study participants. BiB = Born in Bradford.

† = Eligible BiB children with visual acuity measurements for both right and left eyes and additionally a literacy score measured during the same school term.
‡ = All BiB children participating in “Starting Schools Programme” who failed vision screening.
§ = Random sample of BiB children participating in “Starting Schools Programme who passed vision screening.
*Treatment group=children who failed vision screening and were referred for cycloplegic assessment.
**Adherent=prescribed spectacles worn at each visual acuity assessment.
***Non-adherent=children who failed to attend cycloplegic examination and also children who attended but failed to wear prescribed spectacles at each visual acuity assessment.

Figure 2. Projected visual acuity (logMAR) trajectory (with 95% confidence intervals) by group over time (child’s age in months) for the better and worse eye, fully adjusted for all early-life and maternal covariates.

Figure 3. Predicted letter-ID scores over time (child’s age in months) based on the trajectories of the visual acuity (adjusted model) of the better eye. The adjusted model includes all early-life and maternal covariates for the comparison, adherent and non-adherent groups.

For peer review only

For peer review only - http://bmjopen.bmj.com/site/about/guidelines.xhtml

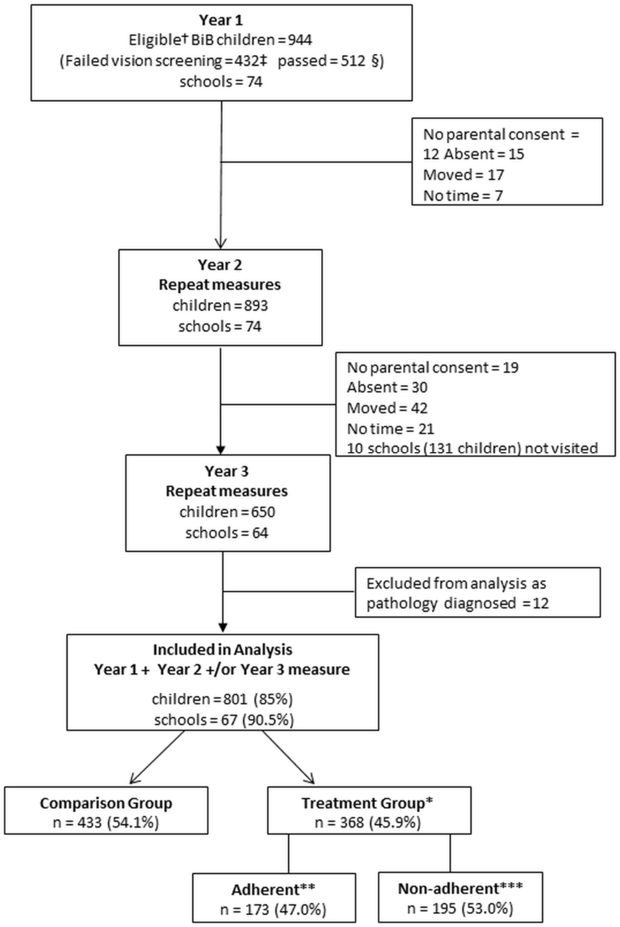

Figure1. Flow chart of the study participants. BiB = Born in Bradford.

† =Total number of eligible BiB children.
‡ =All BiB children who failed vision screening and additionally had a literacy score measured during the same school term.
§=Random sample of BiB children who passed vision screening and additionally had a literacy score measured during the same school term.
*Treatment group=children who failed vision screening and were referred for cycloplegic assessment.
**Adherent=prescribed spectacles worn at each visual acuity assessment.
***Non-adherent=children who failed to attend cycloplegic examination and also children who attended but failed to wear prescribed spectacles at each visual acuity assessment.

190x275mm (300 x 300 DPI)

For peer review only

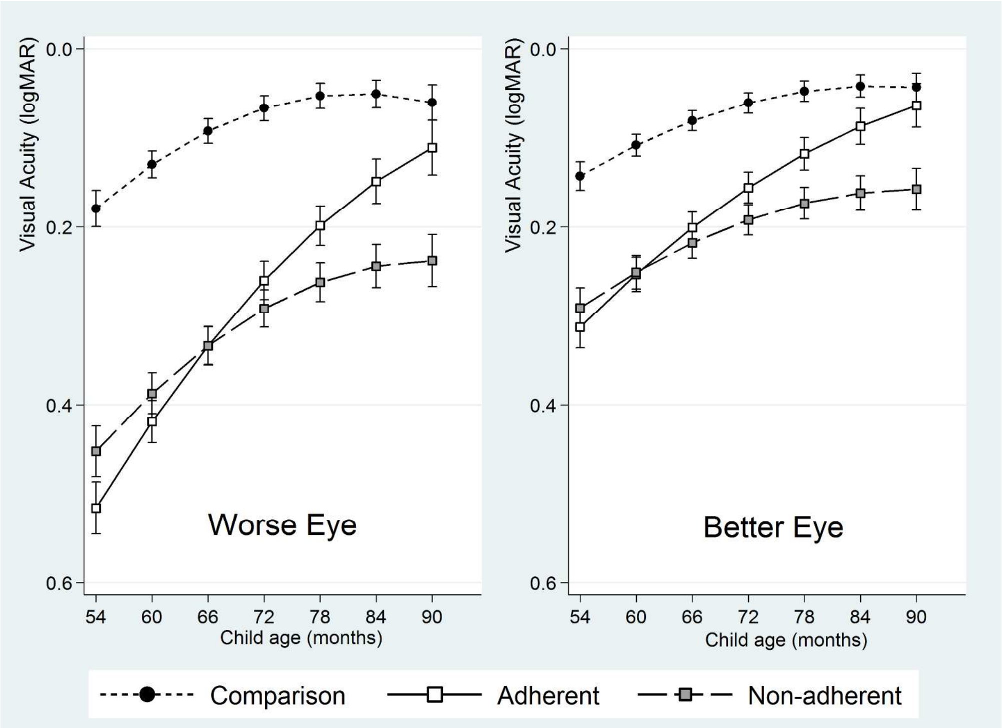

Figure 2. Projected visual acuity (logMAR) trajectory (with 95% confidence intervals) by group over time (child's age in months) for the better and worse eye, fully adjusted for all early-life and maternal covariates.

254x184mm (300 x 300 DPI)

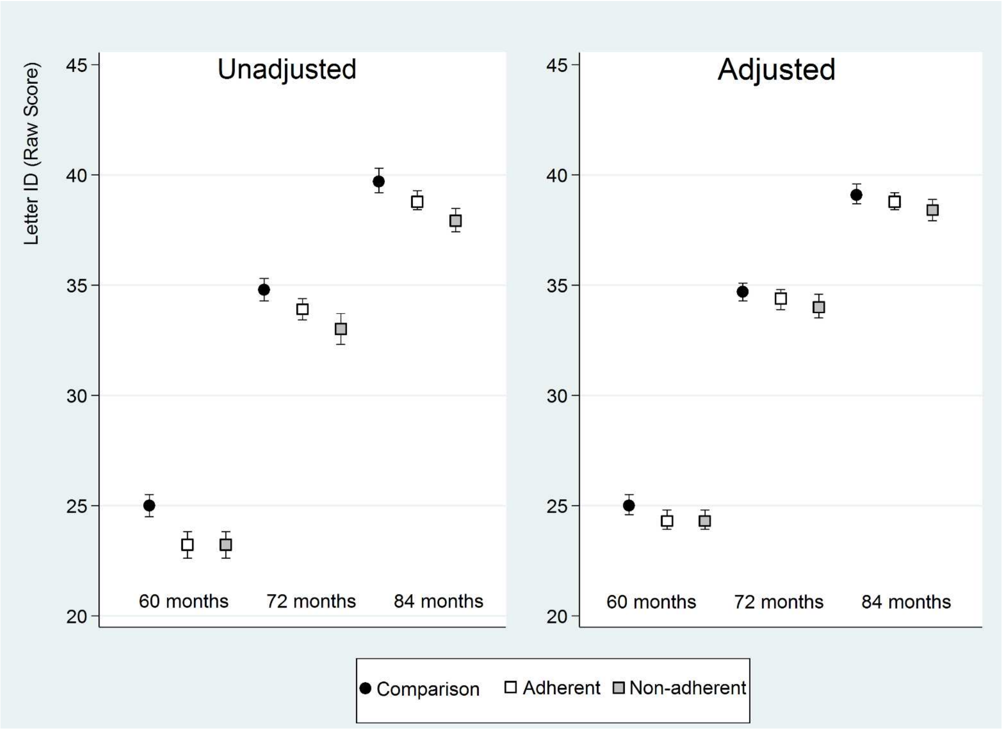

Figure 3. Predicted letter-ID scores over time (child's age in months) based on the trajectories of the visual acuity (adjusted model) of the better eye. The adjusted model includes all early-life and maternal covariates for the comparison, adherent and non-adherent groups.

254x184mm (300 x 300 DPI)

For peer review only - http://bmjopen.bmj.com/site/about/guidelines.xhtml

STROBE (Strengthening The Reporting of OBservational Studies in Epidemiology) Checklist

A checklist of items that should be included in reports of observational studies. You must report the page number in your manuscript where you consider each of the items listed in this checklist. If you have not included this information, either revise your manuscript accordingly before submitting or note N/A.

Note: An Explanation and Elaboration article discusses each checklist item and gives methodological background and published examples of transparent reporting. The STROBE checklist is best used in conjunction with this article (freely available on the Web sites of PLoS Medicine at http://www.plosmedicine.org/, Annals of Internal Medicine at http://www.annals.org/, and Epidemiology at http://www.epidem.com/). Information on the STROBE Initiative is available at www.strobe-statement.org.

Section and Item	Item No.	Recommendation	Reported on Page No.
**Title and Abstract**	1	(*a*) Indicate the study's design with a commonly used term in the title or the abstract	
		(*b*) Provide in the abstract an informative and balanced summary of what was done and what was found	
**Introduction**			
Background/Rationale	2	Explain the scientific background and rationale for the investigation being reported	
Objectives	3	State specific objectives, including any prespecified hypotheses	
**Methods**			
Study Design	4	Present key elements of study design early in the paper	
Setting	5	Describe the setting, locations, and relevant dates, including periods of recruitment, exposure, follow-up, and data collection	
Participants	6	(*a*) *Cohort study*—Give the eligibility criteria, and the sources and methods of selection of participants. Describe methods of follow-up<br>*Case-control study*—Give the eligibility criteria, and the sources and methods of case ascertainment and control selection. Give the rationale for the choice of cases and controls<br>*Cross-sectional study*—Give the eligibility criteria, and the sources and methods of selection of participants	
		(*b*) *Cohort study*—For matched studies, give matching criteria and number of exposed and unexposed<br>*Case-control study*—For matched studies, give matching criteria and the number of controls per case	
Variables	7	Clearly define all outcomes, exposures, predictors, potential confounders, and effect modifiers. Give diagnostic criteria, if applicable	

For peer review only

Section and Item	Item No.	Recommendation	Reported on Page No.
Data Sources/ Measurement	8*	For each variable of interest, give sources of data and details of methods of assessment (measurement). Describe comparability of assessment methods if there is more than one group	
Bias	9	Describe any efforts to address potential sources of bias	
Study Size	10	Explain how the study size was arrived at	
Quantitative Variables	11	Explain how quantitative variables were handled in the analyses. If applicable, describe which groupings were chosen and why	
Statistical Methods	12	(*a*) Describe all statistical methods, including those used to control for confounding	
		(*b*) Describe any methods used to examine subgroups and interactions	
		(*c*) Explain how missing data were addressed	
		(*d*) *Cohort study*—If applicable, explain how loss to follow-up was addressed<br>*Case-control study*—If applicable, explain how matching of cases and controls was addressed<br>*Cross-sectional study*—If applicable, describe analytical methods taking account of sampling strategy	
		(*e*) Describe any sensitivity analyses	
**Results**			
Participants	13*	(a) Report numbers of individuals at each stage of study—eg numbers potentially eligible, examined for eligibility, confirmed eligible, included in the study, completing follow-up, and analysed	
		(b) Give reasons for non-participation at each stage	
		(c) Consider use of a flow diagram	
Descriptive Data	14*	(a) Give characteristics of study participants (eg demographic, clinical, social) and information on exposures and potential confounders	
		(b) Indicate number of participants with missing data for each variable of interest	
		(c) *Cohort study*—Summarise follow-up time (eg, average and total amount)	
Outcome Data	15*	*Cohort study*—Report numbers of outcome events or summary measures over time	
		Case-control study—Report numbers in each exposure category, or summary measures of exposure	
		Cross-sectional study—Report numbers of outcome events or summary measures	

For peer review only

Section and Item	Item No.	Recommendation	Reported on Page No.
Main Results	16	(*a*) Give unadjusted estimates and, if applicable, confounder-adjusted estimates and their precision (eg, 95% confidence interval). Make clear which confounders were adjusted for and why they were included	
		(*b*) Report category boundaries when continuous variables were categorized	
		(*c*) If relevant, consider translating estimates of relative risk into absolute risk for a meaningful time period	
Other Analyses	17	Report other analyses done—eg analyses of subgroups and interactions, and sensitivity analyses	
**Discussion**			
Key Results	18	Summarise key results with reference to study objectives	
Limitations	19	Discuss limitations of the study, taking into account sources of potential bias or imprecision. Discuss both direction and magnitude of any potential bias	
Interpretation	20	Give a cautious overall interpretation of results considering objectives, limitations, multiplicity of analyses, results from similar studies, and other relevant evidence	
Generalisability	21	Discuss the generalisability (external validity) of the study results	
**Other Information**			
Funding	22	Give the source of funding and the role of the funders for the present study and, if applicable, for the original study on which the present article is based	

*Give information separately for cases and controls in case-control studies and, if applicable, for exposed and unexposed groups in cohort and cross-sectional studies.

Once you have completed this checklist, please save a copy and upload it as part of your submission. DO NOT include this checklist as part of the main manuscript document. It must be uploaded as a separate file.

For peer review only

BMJ Open

Effect of adherence to spectacle wear on early developing literacy: A longitudinal study based in a large multi-ethnic city, Bradford, UK.

Journal:	*BMJ Open*
Manuscript ID	bmjopen-2017-021277.R1
Article Type:	Research
Date Submitted by the Author:	09-Mar-2018
Complete List of Authors:	Bruce, Alison; Bradford Institute for Health Research<br>Kelly, Brian; Bradford Institute for Health Research<br>Chambers, Bette; University of York, Institute for Effective Education<br>Barrett, Brendan; University of Bradford, School of Optometry and Vision Science<br>Bloj, Marina; University of Bradford, School of Optometry and Vision Science<br>Bradbury, John; Bradford Teaching Hospitals NHS Foundation Trust<br>Sheldon, Trevor; University of York, health Sciences
**Primary Subject Heading**:	Public health
Secondary Subject Heading:	Ophthalmology, Paediatrics, Public health
Keywords:	visual acuity, vision screening, literacy, spectacles, adherence

SCHOLARONE™ Manuscripts

only

Effect of adherence to spectacle wear on early developing literacy: A longitudinal study based in a large multi-ethnic city, Bradford, UK.

Alison Bruce (corresponding author), Post-doctoral Research Fellow and Head Orthoptist
Bradford Institute for Health Research, Bradford Teaching Hospitals NHS Trust, Duckworth Lane, Bradford, UK BD9 6RJ
01274 383414
alison.bruce@bthft.nhs.uk

Brian Kelly, Statistician
Bradford Institute for Health Research, Bradford Teaching Hospitals NHS Trust, Duckworth Lane, Bradford, UK BD9 6RJ
01274 383466
brian.kelly@bthft.nhs.uk

Bette Chambers, Professor and Director of Institute for Effective Education
University of York, Heslington, York, UK YO10 5DD
bette.chambers@york.ac.uk

Brendan T Barrett, Professor of Visual Development
School of Optometry and Vision Science, University of Bradford, Richmond Rd, Bradford, UK BD7 1DP
01274 235589
B.T.Barrett@bradford.ac.uk

Marina Bloj, Professor of Visual Perception
School of Optometry and Vision Science, University of Bradford, Richmond Rd, Bradford, UK BD7 1DP
01274 236258
M.Bloj@bradford.ac.uk

John Bradbury, Consultant Paediatric Ophthalmologist
Bradford Teaching Hospitals Foundation Trust, Duckworth Lane, Bradford, UK BD9 6RJ
john.bradbury@bthft.nhs.uk

Trevor A Sheldon, Professor of Health Services Research and Policy
Department of Health Sciences, University of York, Heslington, York, UK YO10 5DD
01904 321521
Trevor.Sheldon@york.ac.uk

Word count: 4694

For peer review only - http://bmjopen.bmj.com/site/about/guidelines.xhtml

ABSTRACT

Objectives: To determine the impact of adherence to spectacle wear on visual acuity and developing literacy following vision screening at age 4-5 years.

Design: Longitudinal study nested within the Born in Bradford birth cohort.

Setting and participants: Observation of 944 children; 432 had failed vision screening and were referred (treatment group) and 512 randomly selected (comparison group) who had passed (<0.20 logMAR in both eyes). Spectacle wear was observed in school for two years following screening and classified as adherent, (wearing spectacles at each assessment), or non-adherent.

Main outcome measures: Annual measures of visual acuity (VA) using a crowded logMAR Test. Literacy was measured by Woodcock Reading Mastery Tests-Revised subtest: letter identification.

Results: The VA of all children improved with increasing age, -0.009 log units per month (95% CI: -0.011 to -0.007) (worse eye).The VA of the adherent group improved significantly more than the comparison group, by an additional -0.008 log units per month (95% CI: -0.009 to -0.007) (worse eye) and -0.004 log units per month (95% CI: -0.005 to -0.003) in the better eye.

Literacy was associated with the VA, letter-ID reduced by -0.9 (95% CI:-1.15 to -0.64) for every one line (0.10 logMAR) fall in VA (better eye). This association remained after adjustment for socioeconomic and demographic factors (-0.360, 95% CI:-0.57 to -0.149). The adherent group consistently demonstrated higher letter-ID scores compared to the non-adherent group, with the greatest effect size (0.11) in Year 3.

For peer review only - http://bmjopen.bmj.com/site/about/guidelines.xhtml

Conclusions: Early literacy is associated with the level of VA; children who adhere to spectacle wear improve their VA and also have the potential to improve literacy. Our results suggest failure to adhere to spectacle wear has implications for the child's vision and education.

For peer review only

For peer review only - http://bmjopen.bmj.com/site/about/guidelines.xhtml

Strengths and limitations of this study

- This is the first longitudinal study to compare the effects of adherence and non-adherence to spectacles in children following vision screening at age 4-5 years on both visual acuity and developing literacy.
- Nesting the study within the Born in Bradford birth cohort allows adjustment for confounding factors.
- The study is observational in nature reflecting real life adherence to spectacle wear.
- The study is not a randomised controlled trial therefore allocation to the adherent or non-adherent groups is not exact and may underestimate the effect of non-adherence.

For peer review only

INTRODUCTION

Visual development in humans occurs in early-life[1] with the presence of reduced visual acuity (VA) in young children potentially indicating an associated condition such as significant refractive error, strabismus and/or amblyopia.[2] The UK National Screening Committee (UK NSC) recommends visual screening for all children at age 4-5 years,[3] that is in the first year of school, in order to identify a reduction in VA. For those who fail their test (>0.20 logMAR in one or both eyes)[3] the follow-up clinical pathway includes referral for a cycloplegic refraction and fundus examination to determine the presence and magnitude of any refractive error and to rule out eye disease.[4] In those with reduced VA, treatment generally consists of the wearing of spectacles[5] and may be combined with occlusion therapy[6] (wearing an eye patch or atropine drops). However, adherence to treatment, both spectacle wear[7,8] and occlusion therapy is known to be variable.[9]

Decreased VA, both near and distance and also the presence of refractive error in young children has been reported to be associated with reduced literacy levels.[10-12] However, there is a paucity of evidence on the impact of non-adherence to spectacle wear on VA and early developing literacy in children. Early literacy skills such as letter recognition,[13] word reading and decoding[14] taught in the first years of school are indicators of future reading performance and educational attainment, which in turn affect long-term health and social outcomes.[15,16] The initial school years are a crucial time for the development of these key literacy skills[17] and it is important to understand the impact of non-adherence to spectacle wear on visual outcome and educational attainment.

Low educational attainment is associated with socioeconomic deprivation,[16] which makes the investigation of the relationship between visual acuity and literacy difficult, as in order to account for potential confounding factors, comprehensive epidemiological data are required. Born in Bradford (BiB) is a large birth cohort, which collected maternal and early-life measures from mothers and their children in Bradford and details of recruitment have been previously reported.[18] By linking separately-collected vision and literacy data in children in the BiB cohort, we had the opportunity to explore the association between VA, spectacle

For peer review only - http://bmjopen.bmj.com/site/about/guidelines.xhtml

wear and literacy development whilst taking into account the effects of potential confounders. The aim of this study is to examine the impact of adherence to spectacle wear on early developing VA and literacy skills in children during their first three years of school.

METHODS

This is a prospective, longitudinal study nested within the BiB cohort following children from the point of their initial vision screening at age 4 -5 years. The study took place between 2012 and 2015. Baseline epidemiological data collected from mothers and children of the BiB cohort, literacy measures, vision screening results and repeat measures of vision and literacy were linked in order to investigate the longitudinal impact of adherence to spectacle wear on VA and early literacy.

Population

All children invited to join the study were participating in the BIB,[18] a longitudinal, multi-ethnic birth cohort study aiming to examine the impact of environmental, psychological and genetic factors on maternal and child health and wellbeing. Bradford is an ethnically diverse city (approximately half of the births are to mothers of South Asian origin) with high levels of socio-economic deprivation. The cohort is broadly representative of the city's maternal population.

Patient and Public Involvement

The Born in Bradford (BiB) project team was established with an emphasis on the importance of involving parents and ensuring they are central to the research that is prioritised; what is important to the parents, how people find out the results from the research projects, and what it means for their families. The participants were asked their views on many research topics including literacy levels, vision and the impact of vision on literacy. The participants suggested that these topics are of high importance and should be prioritised. The preliminary findings have been reported to the parents to provide verification

For peer review only - http://bmjopen.bmj.com/site/about/guidelines.xhtml

of the data, ensuring that the findings reflect true patient experiences. Their ideas are essential in developing and revising current information provided to parents and carers. Their involvement has allowed the research to be prioritised around the needs and requirements of patients and carers. Finally in the dissemination of the research results the parents will be central to publicising this study and its findings to local people, schools and the wider community.

Recruitment

As part of the separate BiB “Starting Schools Programme”, children’s literacy levels on school entry (termed ‘Reception Class’ in England, UK and defined as Year 1 of this study) were measured between September 2012 and July 2014. Seventy-four of the one hundred and twenty-three Bradford primary schools (60%) participated in “Starting Schools Programme” and these schools were also invited to join the vision and literacy study. Of the 2930 BiB children (74 schools), 432 (14.7%) had failed vision screening (Figure 1). These children were referred for follow-up cycloplegic investigation and are defined as the treatment group. A further 512 children from the same schools (randomly selected using Excel’s random number generator) who had passed vision screening were also invited to participate and were defined as the comparison group, giving a total of 944 participants in the study. Consent was opt-out and parents received a letter via the schools requesting continued participation prior to each annual assessment. 893 of the 944 (94.6%) consented to participate in Year 2 and 650/944 (68.9%) participated in Year 3 (Figure 1).

Baseline Vision Assessments – Year 1

The vision screening programme for 4-5 year old children in Bradford is conducted in the first year of school by orthoptists with 97% of eligible children being screened.[19] The screening includes standard protocols for measurement of monocular distance VA.[20,21] VA was measured at a distance of three metres using the LogMAR Crowded Test (Keeler, Windsor, UK) which has four letters per line, with each letter having a score of 0.025; the total score

For peer review only - http://bmjopen.bmj.com/site/about/guidelines.xhtml

for each line thus represents 0.10 log unit (Supplementary Information 1). A matching card was used and knowledge of letters was not therefore necessary to perform the test. VA was measured to threshold (i.e. best achievable VA with no defined endpoint). In addition cover test, ocular motility and non-cycloplegic auto refraction using Welch Allyn SureSight (Welch Allyn medical products,Skaneateles. NY) were performed. The data formed the baseline vision data (Year 1). No child in the study was wearing spectacles at the baseline assessment.

Children failing to achieve the VA pass criterion (>0.20 logMAR in one or both eyes) set by the UK National Screening Committee[3] or who had a strabismus detected on cover testing were referred for follow-up. The standard clinical pathway[4] following vision screening entailed referral to either to a community optometrist or the hospital eye service where a cycloplegic refraction (1% cyclopentolate hydrochloride) and fundus examination were undertaken, either by a paediatric ophthalmologist or an optometrist. Spectacles were prescribed based on the result of the cycloplegic refraction and clinical judgement; children were generally prescribed spectacles, including low degrees of hypermetropia, if they had a reduced VA. A follow-up appointment was then arranged with the orthoptist approximately 8 weeks after the cycloplegic examination to repeat the VA measurement, with the child wearing spectacles if they had been prescribed. Children assessed by a community optometrist of their choice had the results of their examination returned to the hospital eye service and also had a follow-up appointment arranged with an orthoptist.

All VA testing, both at the point of vision screening and at follow-up, was performed using the same method of measurement. The results of the follow-up assessment including cycloplegic refraction, VA with the prescribed glasses, cover testing and fundus and media examination were extracted from the medical notes. The ophthalmic staff did not have knowledge of the baseline literacy assessment.

For peer review only - http://bmjopen.bmj.com/site/about/guidelines.xhtml

For peer review only

Baseline Literacy Assessments – Year 1

As part of the BiB "Starting Schools Programme", literacy was measured on school entry (Year 1) by trained research assistants within the same academic term as the vision screening. The research assistants were unaware of the VA results. An age-appropriate literacy measure, the Woodcock Reading Mastery Tests-Revised (WRMT-R) subtest: Letter Identification (ID), a validated reading skill test, was used to assess early literacy.[22] Letter identification measures the child's ability to identify single letters, an essential skill mastered prior to reading and one of the best predictors of future reading achievement.[15] The letter-ID test is a test of knowledge of letters (the complete alphabet is used) and the child must verbally identify the name of each letter. This literacy measure specifically uses varied font type; the size of the letters approximate to 1.1 log unit (20/250) at 33cm, therefore the performance on this test is not affected by the level of VA. Letter-ID was collected in both raw and age standardised format. In addition receptive vocabulary, an indicator of language ability, was measured using the British Picture Vocabulary Scale (BPVS),[23] providing a representation of early language ability and cognition.

Follow up Assessments - Years 2 and 3

Vision and literacy measures were repeated within the same school term approximately 12 months (Year 2) and 24 months (Year 3) after the baseline measurements. Both the vision and the literacy assessments were administered on the same day by the same personnel who were unaware of the previous year's vision or literacy results. VA and literacy was measured as detailed above. VA found to be ≥0.10 logMAR was repeated with a pinhole and near VA was measured using the Bailey-Lovie near-vision chart.[24] (Supplementary Information 1). Cover test, ocular motility, non-cycloplegic auto refraction Welch Allyn SureSight (Welch Allyn medical products, Skaneateles. NY) and whether the child was wearing spectacles were recorded. In order to present the real-life impact of adherence to spectacle wear, all VA measures reported are presenting visual acuities i.e. measured with

For peer review only - http://bmjopen.bmj.com/site/about/guidelines.xhtml

spectacles if worn at the time of the assessment in school. Parents and children were not given prior warning of these assessments. This study was approved by National Research Ethics Committee Yorkshire & the Humber- South Yorkshire (Ref 13/YH/0379).

Statistical Analysis

Children with baseline data for both vision and literacy in Year 1 and who had at least one follow-up measure in either Year 2 or Year 3 were included in the final analysis (Figure 1). The statistical model selected for the analyses, using projections over time, takes into account missing data and requires a minimum of measures at two time points. Using this type of statistical analysis allows inclusion of a greater number of participants giving maximum power to the analyses.[25] The characteristics of children participating in the study were compared initially using Chi-squared or two sided t-tests as appropriate. Children in the treatment group were retrospectively divided into two sub-groups, adherent and non-adherent. Adherence was defined as wearing prescribed spectacles at the time of assessment; otherwise children were defined as non-adherent. Children who were assessed twice but only wore the spectacles on one occasion were classed as non-adherent. A sensitivity analysis was conducted to assess the extent to which the results varied by changing the definition of adherence.

Analysis of Visual Acuity

To investigate the effect of spectacle wear over time on VA, multilevel longitudinal models[25] were firstly constructed with VA as the outcome measure for the child's better and worse eye. The models measure change within the individual and change between individuals over time and allow for individual differences in the rate of change over time.[25] A quadratic term was included to model the non-linear trajectory of change. The model also includes an interaction term to compare the relationship between age and group, to test whether differences by group are the same at different ages. Unadjusted analysis was initially undertaken with subsequent adjustment for demographic and socioeconomic factors

For peer review only - http://bmjopen.bmj.com/site/about/guidelines.xhtml

reported in the literature to be associated with reduced VA: early-life factors[26] (gender, gestational age, birth weight, route of birth) and maternal factors[27] (ethnicity, mother's age at delivery, mother's level of educational attainment and being in receipt of means-tested benefits). Predicted outcomes were plotted to visualise group differences and change in the outcomes for each group over time.

Analysis of Literacy

In order to estimate the association between the letter-ID and VA the same multilevel and longitudinal modelling approach was adopted, but with the final letter-ID score as the outcome measure. The raw letter-ID scores were used in the analysis in order to explore change over time. After estimating differences between the groups and accounting for the initial letter-ID at baseline (Year 1), further adjustment was undertaken for the factors reported in the literature to be associated with educational attainment, the early-life factors[28] and maternal factors as previously stated.[29] Spherical equivalent refraction (SER) (sphere plus half cylinder) of the better eye was included as was BPVS score in order to account for language ability. The results of these models are presented along with predicted outcomes for each of the groups. Effect sizes are generally reported when appraising educational interventions. To demonstrate group differences at each time point effect sizes were calculated for the letter-ID scores using Cohen's *d*.[30]

Visual Acuity Time Point Three.

Children were unable to accurately perform the near VA (logMAR) test until time point three; we are therefore unable to provide a longitudinal analysis. At time point three we have measures of both near VA and distance VA and present the correlation between the near and distance VA at this time point only. Additionally we analysed association between near VA and literacy to examine if the results differed from the association between distance VA and literacy at time point three only.

All analyses were carried out using Stata V.13 (StataCorp, College Station, Texas, USA).

For peer review only - http://bmjopen.bmj.com/site/about/guidelines.xhtml

For peer review only

RESULTS

Twelve children in the treatment group were excluded from the analysis as they had ocular conditions other than refractive error (e.g. nystagmus) confirmed in their medical notes, leaving 368 children in the treatment group and 433 in the comparison group. Data from 801 (85%) children from 67 schools were included in the final analysis (Figure 1). 230/368 (62.5%) of children in the treatment group had attended for the initial cycloplegic examination and been prescribed spectacles, 3/368 (0.8%) attended but no cycloplegic refraction information was available, 23/368 (6.3%) had been prescribed spectacles but had not returned for follow-up VA assessment, and 112/368 (30.4%) had failed to attend any appointment following vision screening. Of the 253 children in the treatment group with cycloplegic refraction results, 157/253 (62.1%) had astigmatism (>1.00DC) either alone or in combination with hypermetropia or myopia. 35/253 (13.8%) had hypermetropia (>+3.0DS) alone, 11 (4.3%) had myopia (≤-0.50DS) alone and 50 (19.8%) children had low hypermetropia (>+1.0DS to +3.0DS). 55 of 253 (21.7%) additionally had anisometropia (≥1.0D difference). For those children with a cycloplegic refraction result (Table 1) the SER ranged from -7.875 to +7.50D in the better eye and -8.25 to +7.50D in the worse eye. Fourteen of the 368 (3.8%) children had a constant or intermittent strabismus, five of whom had been prescribed occlusion therapy for amblyopia. Those children were not excluded from the analysis as they met the initial VA referral criteria and had been prescribed spectacles.

Baseline (Year 1) characteristics of the children in the comparison and treatment groups are shown in Table 1. A small mean difference (-0.021 logMAR, 95% CI -0.022 to -0.020) in VA between the eyes of the comparison group was found, equating to one letter difference. This is not clinically significant but is statistically significant therefore VA's are presented for the better and worse eye separately. Higher levels of VA were found in both eyes of the comparison group compared to the treatment group (Chi-squared p<0.001) (Table 1). The only demographic factor found to differ between the comparison and the treatment group

For peer review only - http://bmjopen.bmj.com/site/about/guidelines.xhtml

was the average mother's age which was around 10 months more in the treatment group (Chi-squared p<0.001).

Table 1. Characteristics of Born in Bradford children and mothers included in the analyses. Values are numbers (%) or mean (SD).

	Comparison group n=433	Treatment group n=368	P value†
**Children**			
Age (months) Year 1	60 (4.2)	60 (4.5)	0.119
Gender			
Male	229 (51.1)	183 (49.7)	
Female	219 (48.9)	185 (50.3)	0.693
Ethnicity			
White	125 (28.0)	91 (24.9)	
Pakistani	262 (58.7)	232 (63.4)	
Other	59 (13.3)	43 (11.7)	0.403
Route of birth			
Vaginal	342 (77.0)	291 (79.7)	
Caesarean	102 (23.0)	74 (20.3)	0.355
Gestational age at birth (weeks)	277 (12.0)	276 (13.0)	0.158
Birth weight (g)	3184 (550.0)	3128 (573.0)	0.155
VA better eye	0.113 (0.049)	0.271 (0.138)	<0.001
VA worse eye	0.135 (0.046)	0.428 (0.189)	<0.001
SER better eye ‡	-	1.19 (0.95)	-
SER worse eye ‡	-	1.98 (1.27)	-
**Mother**			
Age (years)	27.3 (5.4)	28.1 (5.7)	<0.001
Mother's education			
<A-level	227 (64.5)	190 (69.3)	
A-level or above	125 (35.5)	84 (31.7)	0.201
In receipt of means tested benefits (yes)	163 (45.0)	144 (50.1)	0.139

†Difference between Comparison and treatment groups (chi-squared or t-test as appropriate).
VA = visual acuity. VA's are measured in logMAR; therefore higher values represent poorer VA.
SER= spherical equivalent refraction. ‡Cycloplegic results were available for the treatment group only.

Table 2 presents the baseline (Year 1) characteristics of those children in the treatment group retrospectively categorised as adherent (173/368, 47.0%) and non-adherent (195/368, 53.0%) (Figure1). In the non-adherent group, no child wore spectacles at their Year 2 assessment and 39/195 (20%) wore them in Year 3 only. At baseline, the group subsequently classed as adherent had a lower level of VA compared to the non-adherent group in both the better and worse eye (Table 2). The only other factor that differed between the adherent and the non-adherent groups was the mother's level of education with 50/173

For peer review only - http://bmjopen.bmj.com/site/about/guidelines.xhtml

(39.1%) of adherent children having mothers educated to A-level or above compared to only 34/195 (23.3%) of the non-adherent group (Chi-squared p=0.005). Language ability (BPVS) did not differ between the adherent and non-adherent children (p=0.553), suggesting that there were no differences in cognitive ability.

Table 2. Baseline characteristics of participants in the treatment group retrospectively classed as adherent and non-adherent. Values are numbers (%) or mean (SD).

	Adherent n=173 (47.0%)	Non-adherent n=195 (53.0%)	P value†
**Children**			
Age (months) Year 1	59.4 (4.5)	59.6 (4.5)	0.850
Gender			
Male	81 (46.8)	102 (52.3)	
Female	92 (53.2)	93 (47.7)	0.293
Ethnicity			
White	48 (27.9)	43 (22.2)	
Pakistani	103 (59.9)	129 (66.5)	
Other	21 (12.2)	22 (11.3)	0.387
Route of birth			
Vaginal	137 (79.6)	154 (79.8)	
Caesarean	35 (20.4)	39 (20.2)	0.973
Gestational age at birth (weeks)	276 (13.0)	275 (14.0)	0.383
Birth weight (g)	3121 (569.0)	3134 (579.0)	0.833
VA better eye‡	0.292 (0.150)	0.256 (0.129)	0.008
VA worse eye‡	0.465 (0.197)	0.399 (0.175)	0.001
SER better eye	1.18 (0.86)	1.20 (1.02)	0.960
SER worse eye	2.02 (1.20)	1.96 (1.33)	0.657
Language ability scores§	97.8 (15.6)	96.8 (16.4)	0.553
**Mother**			
Age (years)	28.1 (5.8)	28.0 (5.7)	0.845
Mother's education			
<A-level	78 (60.9)	112 (76.7)	
A-level or above	50 (39.1)	34 (23.3)	0.005
In receipt of means tested benefits (yes)	61 (45.5)	83 (55.7)	0.087

†Difference between Adherent and non-adherent treatment groups (chi-squared or t-test as appropriate).
VA = visual acuity. VA's are measured in logMAR; therefore higher values represent poorer VA.
SER= spherical equivalent refraction. §Age-adjusted language ability measure for British Picture Vocabulary Score (BPVS).
‡No child was wearing spectacles at the baseline assessment.

Visual Acuity

At baseline, both the adherent (mean diff: 0.337 logMAR; 95% CI: 0.304 to 0.370) and non-adherent groups (mean diff: 0.273 logMAR; 95% CI: 0.241 to 0.305) had lower levels of VA in the worse eye compared to the comparison group. Table 3 and Figure 2 present the VA trajectories over the course of the study. These show that after adjusting for previously

For peer review only - http://bmjopen.bmj.com/site/about/guidelines.xhtml

described early-life and maternal variables, the VA of both eyes for all three groups; the comparison, the adherent and the non-adherent groups improve over time.

The VA of all children improved with increasing age, -0.009 log units per month (95% CI: -0.011 to -0.007) (worse eye) (Table 3).

Over and above this improvement the adherent group (worse eye) improved by a further -0.008 log units per month (95% CI: -0.009 to -0.007). The adherent children therefore improved overall by -0.017 (95% CI -0.020 to – 0.015) log units per month (95% CI: -0.009 to-0.007) (approximately two letters every 3 months) and also demonstrated a small amount of improvement in the better eye above that expected from age (Table 3).

The non-adherent group (worse eye) improved by -0.003 log units per month (95% CI: -0.004 to -0.001) above that expected from age. The non-adherent children therefore improved overall by -0.012 log units per month (95% CI: -0.014 to -0.010). No additional improvement above that expected from age was demonstrated in the better eye (Table 3).

Sensitivity analysis redefining the classification of adherence did not materially affect the results.

For peer review only

For peer review only - http://bmjopen.bmj.com/site/about/guidelines.xhtml

Table 3. Change in visual acuity for the better and worse eye over time by group; comparison, adherent and non-adherent.

	**Unadjusted (worse eye) (95% CI)**	**Adjusted‡ (worse eye) (95% CI)**	**Unadjusted (better eye) (95% CI)**	**Adjusted‡ (better eye) (95% CI)**
Constant	0.177 (0.159 to 0.194)*	0.386 (0.124 to 0.648)*	0.240 (0.026 to 0.454)*	0.240 (0.026 to 0.454)*
Age (months)	-0.009 (-0.011 to -0.008)***	-0.009 (-0.011 to -0.007)***	-0.006 (-0.008 to -0.005)***	-0.006 (-0.008 to -0.005)***
Age (months) squared	0.00016 (0.00012 to 0.00020)***	0.00016 (0.00012 to 0.00021)***	0.00010 (0.00007 to 0.00013)***	0.00010 (0.00006 to 0.00014)***
Group (reference: comparison)				
Adherent	0.337 (0.309 to 0.366)***	0.337 (0.304 to 0.370)***	0.184 (0.162 to 0.106)***	0.170 (0.144 to 0.196)***
Non-adherent	0.277 (0.250 to 0.305)***	0.273 (0.241 to 0.305)***	0.150 (0.128 to 0.172)***	0.148 (0.123 to 0.174)***
Age ^{X} Group interaction				
Age ^{X} adherent	-0.008 (-0.009 to -0.007)***	-0.008 (-0.009 to -0.007)***	-0.004 (-0.005 to -0.004)***	-0.004 (-0.005 to -0.003)***
Age ^{X} non-adherent	-0.003 (-0.003 to -0.001)***	-0.003 (-0.004 to -0.001)***	-0.001 (-0.002 to 0.000)	-0.002 (-0.004 to 0.000)

‡Model adjusted for gender, ethnicity, gestation period, birthweight, birth route, maternal education status, maternal age and means-tested benefit status.
^{X} Interaction between group and age to determine if the effect of being in a particular group changes with age. The total effect for any one group is the coefficient for age **plus** the additional effect of age for that group.
*p < 0.05, **p < 0.01, ***p< 0.001

For peer review only - http://bmjopen.bmj.com/site/about/guidelines.xhtml

Table 4. Associations between Letter-ID score, visual acuity (better eye), maternal and early-life factors.

FACTOR	UNADJUSTED MODEL (95% CI)	p value	FULLY ADJUSTED MODEL (95% CI)	p value
Constant	18.82 (17.91 to 19.73)	<0.001	-20.6 (-28.2 to -13.0)	<0.001
Age	1.30 (1.21 to 1.38)	<0.001	1.28 (1.19 to 1.37)	<0.001
Age squared	-0.02 (-0.02 to -0.02)	<0.001	-0.020 (-0.022 to -0.017)	<0.001
Visual Acuity: change in Letter-ID per 0.1log unit (one line)	-0.90 (-1.15 to -0.64)	<0.001	-0.327 (-0.540 to -0.115)	0.003
Letter ID baseline (Year 1)			0.348 (0.326 to 0.371)	<0.001
BPVS			0.019 (-0.001 to 0.039)	0.064
Ethnicity				
Pakistani heritage			0.668 (-0.016 to 1.353)	0.056
Other			1.174 (1.159 to 2.189)	0.023
Gender				
Female			0.471 (-0.093 to 1.035)	0.102
Birth weight (per 100g)			0.074 (0.008 to 0.141)	0.029
Gestational age (weeks)			-0.053 (-0.257 to 0.151)	0.611
Receiving Benefits			-0.086 (-0.661 to 0.4990)	0.770
Mothers Level of Education	(higher than A-level)		0.765 (0.156 to 1.374)	0.014
Mothers age at birth (years)			-0.048 (-0.100 to 0.005)	0.075

BPVS = British Picture Vocabulary Scale (baseline standardised score).

Literacy

The unadjusted model shows the final letter-ID score reduces by -0.9 units (95% CI:-1.15 to -0.64) for every one line (0.10 logMAR) fall in VA of the better eye (Table 4). This association persists but is weaker after fully adjusting for the socioeconomic and demographic factors, the letter-ID score declines by -0.327 units (95% CI:-0.540 to -0.115) for every one line fall in VA. Separate adjusted analysis of the VA level of the worse eye shows similar results but with weaker association, letter-ID score declines by -0.260 units (95% CI:-0.414 to -0.105) for every one line fall in VA.

Children of mothers educated to A-level or above had increased letter-ID scores (0.765 units; 95% CI: 0.156 to 1.374) compared to those with lower qualifications. Ethnicity other than white British or Pakistani heritage was associated with better letter-ID score, which might reflect the higher number of mothers educated to above A-level in this group. Greater birth weight was also associated with increased letter-ID score (Table 4). Adjustment for SER made no difference and was not associated with letter-ID (p=0.306). It was therefore

For peer review only - http://bmjopen.bmj.com/site/about/guidelines.xhtml

not included in the models. Similarly subsequent analysis replacing SER with refractive error categories did not show an association with letter-ID (Supplementary Information 2).

A predictive model of the letter-ID score over time for children in each group (Figure 3) was constructed using both the unadjusted and adjusted data from the VA trajectories (Table 3) and incorporated into the model reporting letter-ID (Table 4). The unadjusted trajectory shows both adherent and non-adherent groups at baseline have lower letter-ID scores than the comparison group. The predicted trajectory of improvement in the adherent group is greater than the non-adherent group with the later letter-ID scores of the adherent group converging on those of the comparison group by Year 3. The non-adherent group although improving over time does not catch up with the adherent or the comparison groups. After adjusting for socio-economic and demographic variables the trend is similar but with a smaller difference between the groups.

Table 5 presents the effect size of wearing spectacles on the letter-ID scores between the groups annually over the three years of the study. Comparing the letter-ID scores between the adherent and the non-adherent group a gradual increase in the effect size over time is demonstrated with the greatest effect size (0.11) between the adherent and non-adherent groups shown in Year 3.

Visual Acuity at Time Point Three

The results demonstrate a statistically significant correlation between near and distance visual acuity at time point three (Right Eye r =0.663 and Left Eye r = 0.642) (Supplementary Information 3). In addition the association between the near VA and literacy score and distance VA and literacy score are approximately the same (Supplementary Information 4).

For peer review only - http://bmjopen.bmj.com/site/about/guidelines.xhtml

Table 5. Annual Literacy Scores by Group.

Year	Group	Letter-ID (raw score)	Comparison Groups	Effect Size (Cohen's *d*)*
1	Comparison	25	Comparison v Adherent	0.06
	Adherent	24.3	Comparison v Non-adherent	0.06
	Non-adherent	24.3	Adherent v Non-Adherent	0.00†
2	Comparison	34.7	Comparison v Adherent	0.05
	Adherent	34.4	Comparison v Non-adherent	0.13
	Non-adherent	34.0	Adherent v Non-Adherent	0.07
3	Comparison	39.1	Comparison v Adherent	0.08
	Adherent	38.8	Comparison v Non-adherent	0.18
	Non-adherent	38.4	Adherent v Non-Adherent	0.11

*Based on group difference divided by the pooled standard deviation (SD) of Letter-ID score: SD 10.9 at Year 1, 5.6 at Year 2 and 3.8 at Year 3.
† In Year 1 there is no difference as spectacle wear has not commenced.

For peer review only

DISCUSSION

This is the first longitudinal study to assess the effect of adherence/non-adherence to spectacle wear on VA and literacy in children following vision screening. Our results indicate that early developing literacy is affected by the level of VA even after adjusting for socio-economic and demographic factors associated with educational attainment. The letter-ID score declines by approximately 1.5% for every one line of reduction in VA. In this and similar populations [14,31,] where children have been reported to have reduced VA levels (>0.30logMAR in better eye), there is likely to be an impact on developing literacy skills. The effect size (0.11) of being adherent to spectacle wear compared to non-adherence in Year 3 of our study is the same as that reported in a Chinese study providing free spectacles to children[32] and is comparable with reported educational interventions.[33] Thus children who fail vision screening and adhere to spectacle wear have the potential to improve their VA, further influencing early literacy development.

Adherence to spectacle wear is highly influenced by socio-economic and demographic factors, particularly maternal education, a factor that is also known to be associated with educational attainment.[34] Children with reduced VA and who are in less educated families

For peer review only - http://bmjopen.bmj.com/site/about/guidelines.xhtml

are less likely to adhere to treatment, which will further impact on their educational attainment and future life chances. We were however, able to adjust for the many associated maternal and early-years factors, the value of embedding this study within a birth cohort. A study examining academic performance in US schools reports that failing vision screening was predictive of being in the lowest quartile of academic performance.[35] Conversely, a longitudinal study of children aged 9 – 10 years in Singapore, Dirani et al[36] found VA did not play a significant role in predicting academic performance. The children were however older, mainly myopic and only a small number of participants had decreased VA which may account for the difference in their findings relative to ours.

The VA of children in all groups (adherent, non-adherent and comparison group) continued to improve throughout this study. The improvement in VA found in the comparison group is similar to that reported for normal visual development, with optimum VA achieved around 6 years of age.[37,38] The improvement in VA of the worse eye found in adherent children over the time of the study was significantly greater than that expected solely from visual development[39] or indeed from retest variability[40] and was almost double that of the comparison group. Little additional improvement above that expected from visual development was demonstrated in the worse eye of the non-adherent children, an indication that the improvement in the adherent children is not due to regression to the mean. The longitudinal observation of the children demonstrates improvement not only in VA but also in literacy, with the non-adherent group demonstrating persistently lower literacy scores throughout the study, although the effect is attenuated after adjusting for other factors.

Annual improvement in academic achievement is well recognised and is particularly notable in the early years of schooling with the initial improvement thought to be associated with the effect of entering school, combined with rapid early child development followed by a plateau in academic growth as children progress through school grades.[20]

Early literacy development is complex and associated with socio-economic and demographic factors, in particular maternal education. However, even after taking these factors into account VA continues to be associated with literacy; the poorer the level of VA, the greater

For peer review only - http://bmjopen.bmj.com/site/about/guidelines.xhtml

the reduction in the literacy score. In a Singaporean study,[39] a strong association between paternal level of education and academic school performance was reported. As one might expect, higher levels of maternal education have a positive impact on literacy. [41 ,42] In addition, mothers with higher educational attainment are more likely to effectively access health services, and are more likely to adhere to prescribed treatment.[43]

Our study shows an association between VA and literacy score but no association between SER and literacy. Neither did further analysis by refractive error types indicate an association with literacy, this is most likely related to a lack of power due to the small numbers when refractive error is categorised in our study. Our findings differ from previous studies reporting an association between refractive error and literacy.[11 ,12]

Hypermetropia has been reported to be associated with poor literacy. A large cross-sectional American study (VIP-HIP) of pre-school children aged 4-5 years found that children with uncorrected hypermetropia in conjunction with reduced binocular near VA (worse than 20/40) have poorer literacy than those with hypermetropia and a good level of binocular near visual acuity.[12] The VIP-HIP study report that the level of binocular near VA was predictive of literacy scores; with hypermetropic children with binocular near VA better than 20/40, demonstrating literacy scores similar to those children who were emmetropic. Although the VIP-HIP study does not report distance VA levels of the children it does state that the analysis of the distance VA resulted in similar findings, an indication that distance VA levels may also influence early literacy scores.

Astigmatism has also been reported to be associated with reduced literacy. In native American children bilateral uncorrected astigmatism (≥ 1.00 DC) has been reported to reduce reading fluency, and children with moderate astigmatism are reported to have lower VA and fluency than those with no or low astigmatism.[11] The findings reported from both the above studies may indicate that moderate to high degrees of uncorrected hypermetropia or astigmatism which reduce VA is associated with a reduction in literacy scores. Classroom based tasks where fixation frequently changes are reported to require high levels of distance VA (0.33logMAR) and slightly lesser levels of near VA (0.72logMAR)[44] this is most probably

For peer review only - http://bmjopen.bmj.com/site/about/guidelines.xhtml

due to print size for early readers being enlarged. We would suggest therefore that where VA is reduced beyond that required in the learning environment it will impact on a child's developing literacy and hence the association we report between distance VA and literacy. The longitudinal design of this study provides an insight into development of VA and literacy in the early years of schooling, and the use of linked data from the mothers and children participating in the BiB cohort study permitted the many potential confounding factors associated with educational attainment to be accounted for. We include children with a wide range of refractive error and VA's allowing a robust analysis of the influence of both factors on developing literacy. The study does however have some weaknesses. It is not a randomised controlled trial and non-adherence was defined retrospectively by the failure of the child to wear their prescribed glasses at one assessment; it is possible that this was a unique event and is not representative of the child's true adherence to spectacle wear over the course of the study. If this is indeed the case, then the random misclassification is likely to under-estimate the difference found between the adherent and non-adherent groups.[45] In addition the sensitivity analysis redefining non-adherence does not demonstrate any material difference in the results.

A cycloplegic examination was not undertaken for all children and there will be some children with reduced vision who were not identified at screening (false negatives). No child who had a cycloplegic refraction was found to be a false positive but a proportion of the children who failed to attend for the cycloplegic examination may be false positives. This misclassification will similarly be random, underestimating the size of estimates of effect and suggests our estimates may be conservative.[45]

Visual acuity is the sole measure of visual function reported from the study and it is possible other measures of visual function are also associated with academic performance; further research would be required to explore these associations.

During visual maturation, the presence of neurodevelopmental disorders such as refractive error, and strabismus may contribute to a reduction in VA and early intervention is required. This study demonstrates that wearing spectacles is an effective intervention to improve VA,

For peer review only - http://bmjopen.bmj.com/site/about/guidelines.xhtml

and that this will impact positively on developing literacy. The children who do not adhere to spectacle wear are likely to be those in families who are poorer and less educated. Further research is required to better understand the reasons for non-adherence and evaluate interventions to promote adherence to spectacle wear. This has the potential not only to improve vision but also support future life chances in children who may already face educational disadvantage.

For peer review only

For peer review only - http://bmjopen.bmj.com/site/about/guidelines.xhtml

REFERENCES

1. Daw NW. Critical periods and amblyopia. *Arch Ophthalmol* 1998;116(4):502-5.

2. Robaei D, Rose K, Ojaimi E*, et al.* Visual Acuity and the Causes of Visual Loss in a Population-Based Sample of 6-Year-Old Australian Children. *Ophthalmology* 2005;112(7):1275-82.

3. National Screening Committee. The UK NSC policy on Vision defects screening in children 2013. Available: http://www.screening.nhs.uk/vision-child (Accessed 19/12/2017).

4. Public Health England. Child vision screening resources consultation. Available: https://www.gov.uk/government/consultations/child-vision-screening-resources (Accessed 04/08/2017). 2017.

5. Stewart CE, Moseley MJ, Fielder AR*, et al.* Refractive adaptation in amblyopia: quantification of effect and implications for practice. *Br J Ophthalmol* 2004;88(12):1552-6.

6. Pediatric Eye Disease Investigator G, Repka MX, Kraker RT*, et al.* A randomized trial of atropine vs patching for treatment of moderate amblyopia: follow-up at age 10 years. *Arch Ophthalmol* 2008;126(8):1039-44.

7. Aldebasi YH. A descriptive study on compliance of spectacle-wear in children of primary schools at Qassim Province, Saudi Arabia. *Int J Health Sci (Qassim)* 2013;7(3):291-99.

8. Sharma A, Congdon N, Patel M*, et al.* School-based approaches to the correction of refractive error in children. *Surv Ophthalmol* 2012;57(3):272-83.

9. Maconachie GDE, Gottlob I. The challenges of amblyopia treatment. *Biomed J* 2015;38(6):510-16.

10. Bruce A, Fairley L, Chambers B*, et al.* Impact of visual acuity on developing literacy at age 4-5 years: a cohort-nested cross-sectional study. *BMJ Open* 2016;6(2):e010434.

11. Harvey EM, Miller JM, Twelker JD*, et al.* Reading Fluency in School-Aged Children with Bilateral Astigmatism. *Optom Vis Sci* 2016;93(2):118-25.

12. VIP-HIP Study Group, Kulp MT, Ciner E*, et al.* Uncorrected Hyperopia and Preschool Early Literacy: Results of the Vision in Preschoolers-Hyperopia in Preschoolers (VIP-HIP) Study. *Ophthalmol* 2016;123(4):681-9.

For peer review only - http://bmjopen.bmj.com/site/about/guidelines.xhtml

13. Foulin JN. Why is letter-name knowledge such a good predictor of learning to read? *Read Writ* 2005;18(2):129-55.

14. Marchman VA, Fernald A. Speed of word recognition and vocabulary knowledge in infancy predict cognitive and language outcomes in later childhood. *Dev Sci* 2008;11(3):9-16.

15. Dearden L, Sibieta L, Sylva K. The socio-economic gradient in early child outcomes: evidence from the Millennium Cohort Study. *Longit Life Course Stud* 2011;2(1):19 - 40.

16. Marmot M. Fair Society, Healthy Lives.The Marmot review Executive Summary. London: The Marmot Review 2010.

17. Bloom HS, Hill CJ, Black AR*, et al.* Performance Trajectories and Performance Gaps as Achievement Effect-Size Benchmarks for Educational Interventions. *J Res on Educ Eff* 2008;1(4):289-328.

18. Wright J, Small N, Raynor P*, et al.* Cohort Profile: The Born in Bradford multi-ethnic family cohort study. *Int J Epidemiol* 2013;42(4):978-91.

19. Bruce A, Outhwaite L. Uptake, referral and attendance: results from an inner city school based vision screening programme. *Br Ir Orthopt J* 2013;10:41 - 45.

20. McGraw PV, Winn B, Gray LS*, et al.* Improving the reliability of visual acuity measures in young children. *Ophthalmic Physiol Opt* 2000;20(3):173-84.

21. Simmers AJ, Gray LS, Spowart K. Screening for amblyopia: a comparison of paediatric letter tests. *Br J Ophthalmol* 1997;81(6):465-69.

22. Woodcock RW. Woodcock Reading Mastery Tests (Revised) Circle Pines, MN: American Guidance Service, 1987.

23. Dunn LM, Dunn LM, Whetton C*, et al.* British Picture Vocabulary Scale. 2nd edition Windsor, Berks: NFER-Nelson, 1997.

24. Bailey IL, Lovie JE. The design and use of a new near-vision chart. *Am J Optom Physiol Opt* 1980;57(6):378-87.

25. Goldstein H. Models for Repeated Measures Data. Multilevel Statistical Models: John Wiley & Sons, Ltd, 2010:147-60.

For peer review only - http://bmjopen.bmj.com/site/about/guidelines.xhtml

26. Williams C, Northstone K, Howard M*, et al.* Prevalence and risk factors for common vision problems in children: data from the ALSPAC study. *Br J Ophthalmol* 2008;92(7):959-64.

27. Tarczy-Hornoch K, Varma R, Cotter SA*, et al.* Joint Writing Committee for the Multi-Ethnic Pediatric Eye Disease Study the Baltimore Pediatric Eye Disease Study Groups. Risk Factors for Decreased Visual Acuity in Preschool Children: The Multi-Ethnic Pediatric Eye Disease and Baltimore Pediatric Eye Disease Studies. *Ophthalmology* 2011;118(11):2262-73.

28. Burroughs-Lange SG, Douetil J. Literacy progress of young children from poor urban settings: A Reading Recovery comparison study. *Literacy Teaching and Learning* 2007;12(1):19-46.

29. Schoon I, Parsons S, Sacker A*, et al.* Socioeconomic adversity, educational resilience and subsequent levels of adult adaptation. *J Adolesc Res* 2004;19:383-404.

30. Cohen J. *Statistical power analysis for the behavioral sciences (2nd ed). Mahwah, NJ*, 1988.

31. Murthy GV, Gupta SK, Ellwein LB*, et al.* Refractive error in children in an urban population in New Delhi. *Invest Ophthalmol Vis Sci* 2002;43.

32. Ma X, Zhou Z, Yi H*, et al.* Effect of providing free glasses on children's educational outcomes in China: cluster randomized controlled trial. *BMJ* 2014;349.

33. McEwan PJ. Improving Learning in Primary Schools of Developing Countries:A Meta-Analysis of Randomized Experiments. *Rev Educ Res* 2015;85(3):353-94.

34. Neuman S. Lessons from my mother: Reflections on the National Early Literacy Report. *Educ Res* 2010;39(4):301 -04.

35. Krumholtz I. Results from a pediatric vision screening and its ability to predict academic performance. *Optometry (St Louis, Mo)* 2000;71(7):426-30.

36. Dirani M, Zhang X, Goh LK*, et al.* The Role of Vision in Academic School Performance. *Ophthalmic Epidemiol* 2010;17(1):18-24.

For peer review only - http://bmjopen.bmj.com/site/about/guidelines.xhtml

37. Leone JF, Mitchell P, Kifley A*, et al.* Normative visual acuity in infants and preschool-aged children in Sydney. *Acta Ophthalmol* 2014;92(7):e521-e29.

38. Pan Y, Tarczy-Hornoch K, Susan A C*, et al.* Visual Acuity Norms in Preschool Children: The Multi-Ethnic Pediatric Eye Disease Study. *Optom Vis Sci* 2009;86(6):607-12.

39. Sonksen PM, Wade AM, Proffitt R*, et al.* The Sonksen logMAR test of visual acuity: II. Age norms from 2 years 9 months to 8 years. *J AAPOS* 2008;12(1):18-22.

40. Stewart C. Comparison of Snellen and log-based acuity scores for school-aged children. *Br Orthopt J* 2000;57:32-38.

41. Magnuson K. Maternal education and children's academic achievement during middle childhood. *Dev Psychol* 2007;43(6):1497-512.

42. Taylor J, Ennis CR, Hart SA*, et al.* Home environmental and behavioral risk indices for reading achievement. *Learn Individ Differ* 2017;57:9-21.

43. Leurer MD. Perceived Barriers to Program Participation Experienced by Disadvantaged Families. *Int J Health Promot Educ* 2011;49(2):53-59.

44. Narayanasamy S, Vincent SJ, Sampson GP*, et al.* Visual demands in modern Australian primary school classrooms. *Clin Exp Optom* 2016;99(3):233-40.

45. Flegal KM, Brownie C, Haas JD. The effects of exposure misclassification on estimates of relative risk. *Am J Epidemiol* 1986;123(4):736-51.

For peer review only

For peer review only - http://bmjopen.bmj.com/site/about/guidelines.xhtml

Funding: AB is funded by a National Institute for Health Research Post-Doctoral Fellowship Award (PDF-2013-06-050). The Born in Bradford study presents independent research commissioned by the National Institute for Health Research Collaboration for Applied Health Research and Care (NIHR CLAHRC) and the Programme Grants for Applied Research funding scheme (RP-PG-0407-10044). The views expressed are those of the author(s) and not necessarily those of the NHS, the NIHR or the Department of Health.

Competing interest statement: "All authors have completed the Unified Competing Interest form and declare: no support from any organisation for the submitted work; no financial relationships with any organisations that might have an interest in the submitted work in the previous three years, no other relationships or activities that could appear to have influenced the submitted work.

Acknowledgments: We thank all the families and schools who took part in this study, the orthoptists from Bradford Teaching Hospitals Foundation Trust who conducted the vision screening programme, the researchers from the Starting Schools programme who collected the literacy measures, Patrick Friis, Alexandra Morris and Hannah Farrugia who collected follow-up measures and the Data Support Team from Bradford Institute for Health Research who created and maintain the data linkage system.

Contributors: AB initiated the project, designed data collection, monitored data collection for the whole study, wrote the statistical analysis plan, cleaned and analysed the data, and drafted and revised the paper. She is guarantor. BK wrote the statistical analysis plan, cleaned the data and revised the draft paper. BC initiated the project and revised the draft paper. BTB contributed to the design of the study and revised the draft paper. MB contributed to the design of the study and revised the draft paper. JB contributed to the design of the study and revised the draft paper. TAS initiated the project, wrote the statistical analysis plan and revised the draft paper.

For peer review only - http://bmjopen.bmj.com/site/about/guidelines.xhtml

“The Corresponding Author has the right to grant on behalf of all authors and does grant on behalf of all authors, a worldwide licence to the Publishers and its licensees in perpetuity, in all forms, formats and media (whether known now or created in the future), to i) publish, reproduce, distribute, display and store the Contribution, ii) translate the Contribution into other languages, create adaptations, reprints, include within collections and create summaries, extracts and/or, abstracts of the Contribution, iii) create any other derivative work(s) based on the Contribution, iv) to exploit all subsidiary rights in the Contribution, v) the inclusion of electronic links from the Contribution to third party material where-ever it may be located; and, vi) licence any third party to do any or all of the above.”

"The Corresponding Author has the right to grant on behalf of all authors and does grant on behalf of all authors, an exclusive licence on a worldwide basis to the BMJ Publishing Group Ltd to permit this article (if accepted) to be published in BMJ editions and any other BMJPGL products and sublicences such use and exploit all subsidiary rights, as set out in our licence."

Transparency declaration: Dr Alison Bruce, lead author (the manuscript’s guarantor) affirms that the manuscript is an honest, accurate, and transparent account of the study being reported.

Data sharing statement: No additional data is available.

For peer review only

For peer review only - http://bmjopen.bmj.com/site/about/guidelines.xhtml

Figure Legends:

Figure1. Flow chart of the study participants. BiB = Born in Bradford.

† = Eligible BiB children with visual acuity measurements for both right and left eyes and additionally a literacy score measured during the same school term.
‡ = All BiB children participating in “Starting Schools Programme” who failed vision screening.
§ = Random sample of BiB children participating in “Starting Schools Programme who passed vision screening.
*Treatment group=children who failed vision screening and were referred for cycloplegic assessment.
**Adherent=prescribed spectacles worn at each visual acuity assessment.
***Non-adherent=children who failed to attend cycloplegic examination and also children who attended but failed to wear prescribed spectacles at each visual acuity assessment.

Figure 2. Projected visual acuity (logMAR) trajectory (with 95% confidence intervals) by group over time (child’s age in months) for the better and worse eye, fully adjusted for all early-life and maternal covariates.

Figure 3. Predicted letter-ID scores over time (child’s age in months) based on the trajectories of the visual acuity (adjusted model) of the better eye. The adjusted model includes all early-life and maternal covariates for the comparison, adherent and non-adherent groups.

For peer review only

For peer review only - http://bmjopen.bmj.com/site/about/guidelines.xhtml

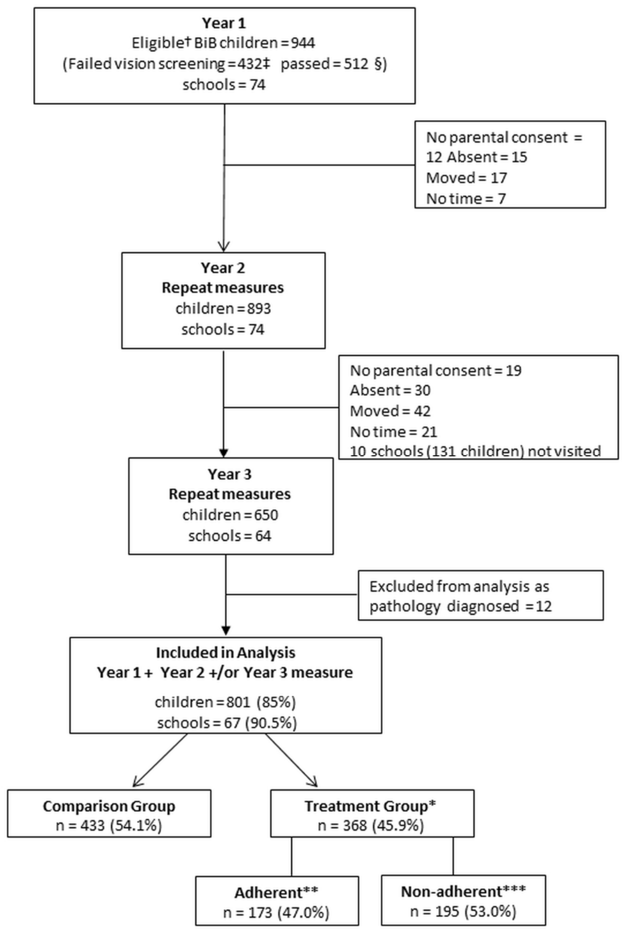

Figure1. Flow chart of the study participants. † BiB = Born in Bradford.
† = Eligible BiB children with visual acuity measurements for both right and left eyes and additionally a literacy score measured during the same school term.
‡ = All BiB children participating in "Starting Schools Programme" who failed vision screening.
§ = Random sample of BiB children participating in "Starting Schools Programme who passed vision screening. *Treatment group=children who failed vision screening and were referred for cycloplegic assessment.
**Adherent=prescribed spectacles worn at each visual acuity assessment.
***Non-adherent=children who failed to attend cycloplegic examination and also children who attended but failed to wear prescribed spectacles at each visual acuity assessment.

190x275mm (300 x 300 DPI)

For peer review only

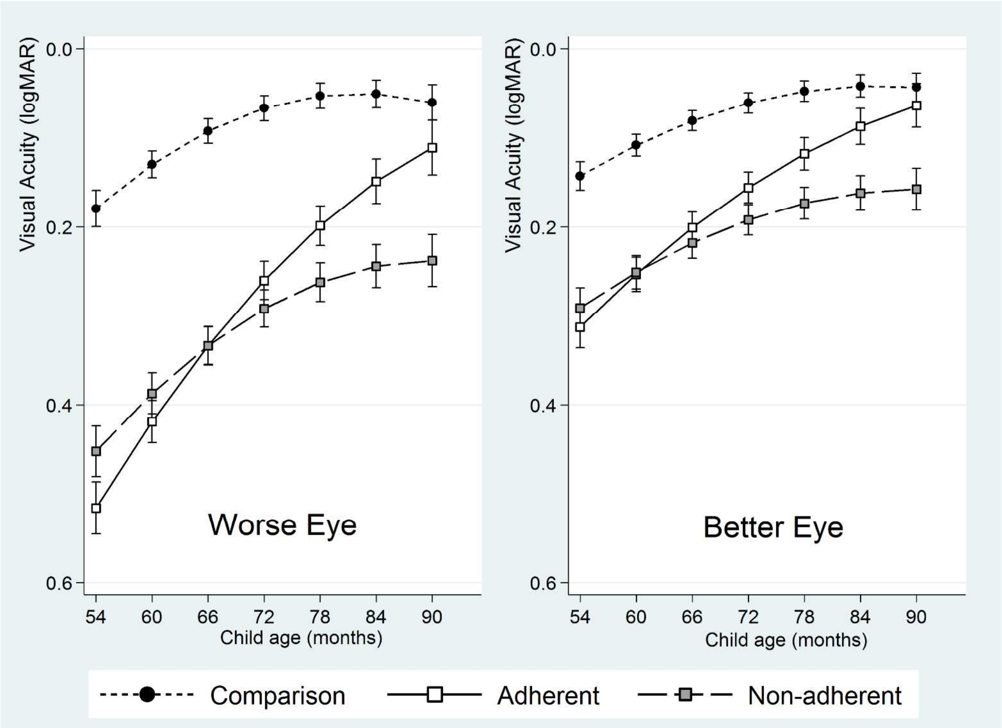

Figure 2. Projected visual acuity (logMAR) trajectory (with 95% confidence intervals) by group over time (child's age in months) for the better and worse eye, fully adjusted for all early-life and maternal covariates.

254x184mm (300 x 300 DPI)

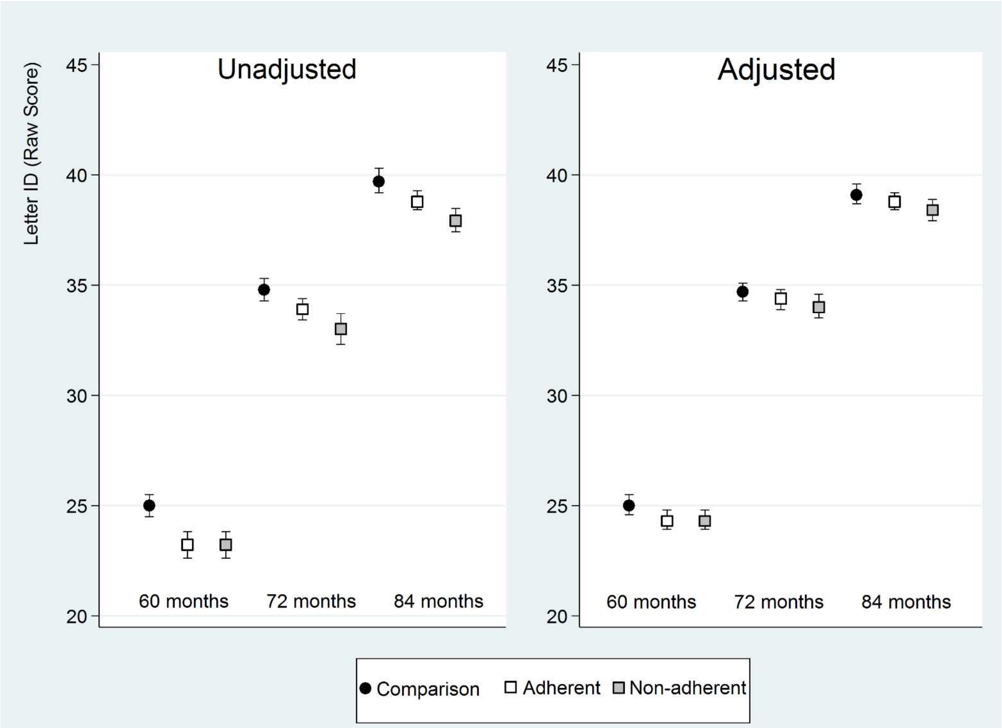

Figure 3. Predicted letter-ID scores over time (child's age in months) based on the trajectories of the visual acuity (adjusted model) of the better eye. The adjusted model includes all early-life and maternal covariates for the comparison, adherent and non-adherent groups.

254x184mm (300 x 300 DPI)

Supplementary Information 1

Keeler Crowded LogMAR Test

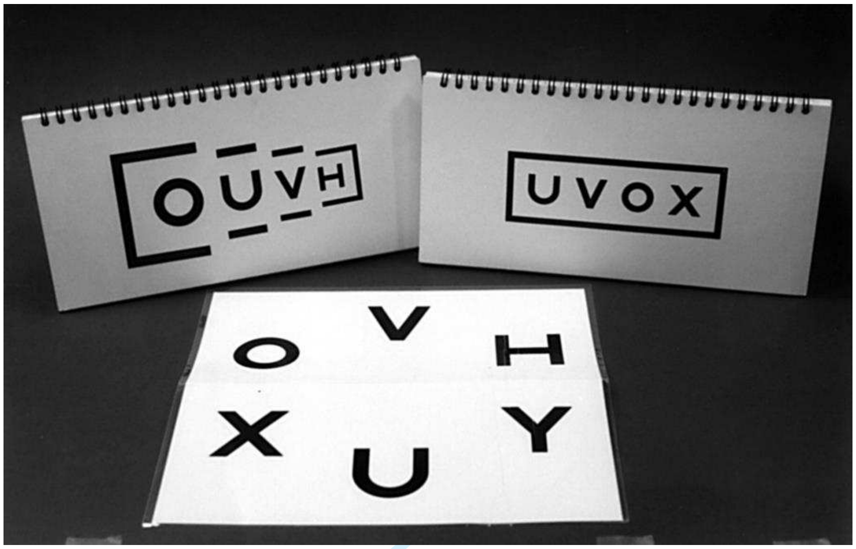

LogMAR Near Vision Test

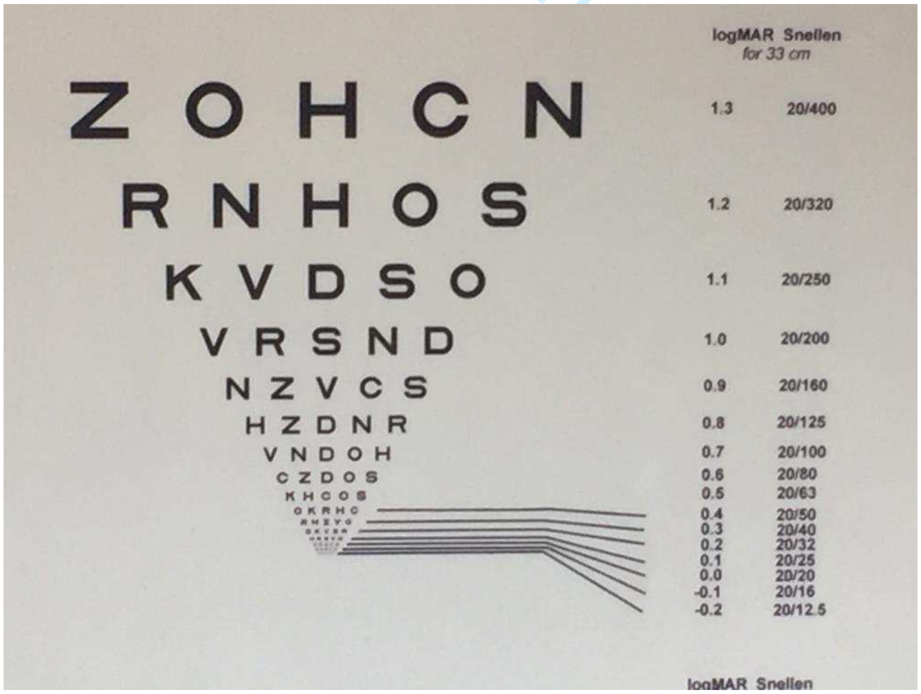

Supplementary Information 2

Associations between Letter-ID score and refractive error types.

FACTOR	FULLY ADJUSTED MODEL (95% CI)	p value
Constant	-21.4 (-29.0 to -13.8)	<0.001
Age	1.32 (1.23 to 1.41)	<0.001
Age squared	-0.021 (-0.023 to -0.018)	<0.001
**Astigmatism**	**-0.329 (-0.933 to 0.275)**	**0.286**
**Hypermetropia**	**-1.071 (-2.586 to 0.444)**	**0.166**
**Myopia**	**1.386 (-2.953 to 5.275)**	**0.531**
**Low hypermetropia**	**0.255 (-0.835 to 1.344)**	**0.647**
Letter ID baseline (Year 1)	0.346 (0.323 to 0.369)	<0.001
BPVS	0.024 (0.004 to 0.044)	0.019
Ethnicity		
Pakistani heritage	0.569 (-0.128 to 1.267)	0.11
Other	1.057 (0.037 to 2.078)	0.042
Gender		
Female	0.667 (0.102 to 1.232)	0.021
Birth weight (per 100g)	0.074 (0.007 to 0.14)	0.029
Gestational age (weeks)	-0.04 (-0.244 to 0.163)	0.698
Receiving Benefits	-0.011 (-0.588 to 0.565)	0.969
Mothers Level of Education		
(higher than A-level)	0.717 (0.11 to 1.325)	0.021
Mothers age at birth (years)	-0.054 (-0.107 to -0.002)	0.042

For peer review only

Supplementary Information 3

Correlation between near and distance visual acuity (Right Eye) at Time Point Three.

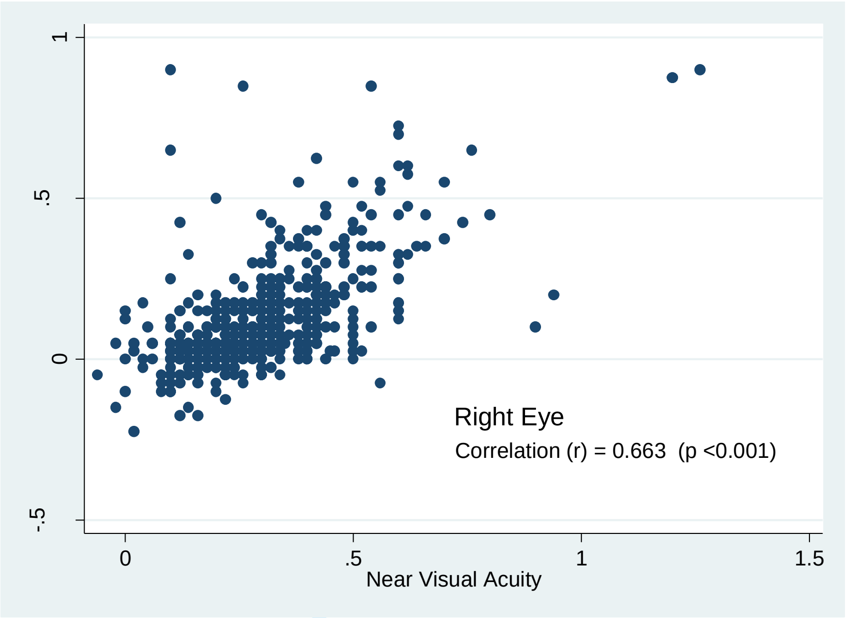

Correlation between near and distance visual acuity (Left Eye) at Time Point Three.

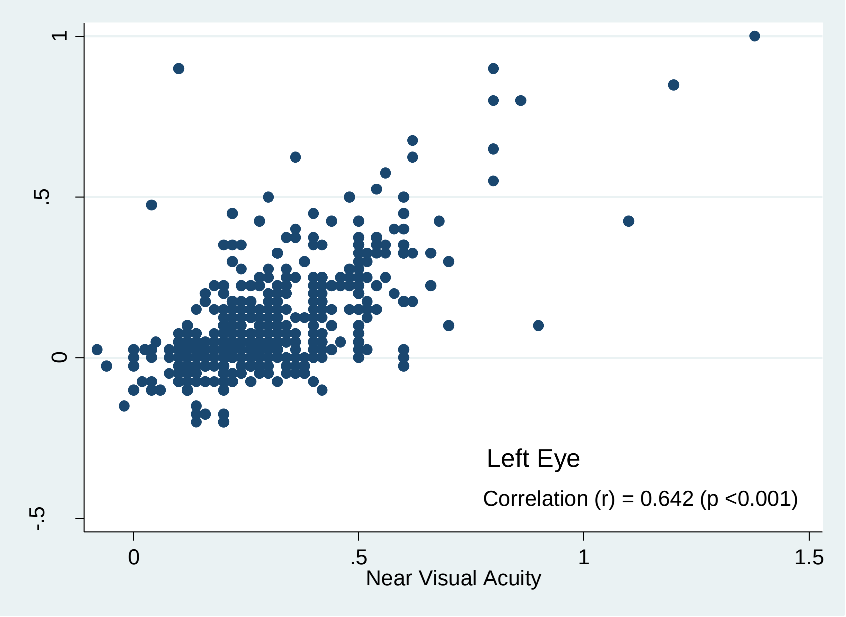

Supplementary Information 4.

Association between visual acuity (distance) and literacy, and between visual acuity (near) and literacy.

	Correlation with Letter ID standardised score at T3	
	**r**	**p-value**
Visual Acuity (far) - Best eye	-0.145	< 0.001
Visual Acuity (far) - Worst eye	-0.183	< 0.001
Visual Acuity (near) – Best eye	-0.115	0.006
Visual Acuity (near) - Worst eye	-0.140	< 0.001

For peer review only

STROBE (Strengthening The Reporting of OBservational Studies in Epidemiology) Checklist

A checklist of items that should be included in reports of observational studies. You must report the page number in your manuscript where you consider each of the items listed in this checklist. If you have not included this information, either revise your manuscript accordingly before submitting or note N/A.

Note: An Explanation and Elaboration article discusses each checklist item and gives methodological background and published examples of transparent reporting. The STROBE checklist is best used in conjunction with this article (freely available on the Web sites of PLoS Medicine at http://www.plosmedicine.org/, Annals of Internal Medicine at http://www.annals.org/, and Epidemiology at http://www.epidem.com/). Information on the STROBE Initiative is available at www.strobe-statement.org.

Section and Item	Item No.	Recommendation	Reported on Page No.
**Title and Abstract**	1	(*a*) Indicate the study's design with a commonly used term in the title or the abstract	
		(*b*) Provide in the abstract an informative and balanced summary of what was done and what was found	
**Introduction**			
Background/Rationale	2	Explain the scientific background and rationale for the investigation being reported	
Objectives	3	State specific objectives, including any prespecified hypotheses	
**Methods**			
Study Design	4	Present key elements of study design early in the paper	
Setting	5	Describe the setting, locations, and relevant dates, including periods of recruitment, exposure, follow-up, and data collection	
Participants	6	(*a*) *Cohort study*—Give the eligibility criteria, and the sources and methods of selection of participants. Describe methods of follow-up<br>*Case-control study*—Give the eligibility criteria, and the sources and methods of case ascertainment and control selection. Give the rationale for the choice of cases and controls<br>*Cross-sectional study*—Give the eligibility criteria, and the sources and methods of selection of participants	
		(*b*) *Cohort study*—For matched studies, give matching criteria and number of exposed and unexposed<br>*Case-control study*—For matched studies, give matching criteria and the number of controls per case	
Variables	7	Clearly define all outcomes, exposures, predictors, potential confounders, and effect modifiers. Give diagnostic criteria, if applicable	

For peer review only

Section and Item	Item No.	Recommendation	Reported on Page No.
Data Sources/ Measurement	8*	For each variable of interest, give sources of data and details of methods of assessment (measurement). Describe comparability of assessment methods if there is more than one group	
Bias	9	Describe any efforts to address potential sources of bias	
Study Size	10	Explain how the study size was arrived at	
Quantitative Variables	11	Explain how quantitative variables were handled in the analyses. If applicable, describe which groupings were chosen and why	
Statistical Methods	12	(*a*) Describe all statistical methods, including those used to control for confounding	
		(*b*) Describe any methods used to examine subgroups and interactions	
		(*c*) Explain how missing data were addressed	
		(*d*) *Cohort study*—If applicable, explain how loss to follow-up was addressed<br>*Case-control study*—If applicable, explain how matching of cases and controls was addressed<br>*Cross-sectional study*—If applicable, describe analytical methods taking account of sampling strategy	
		(*e*) Describe any sensitivity analyses	
**Results**			
Participants	13*	(a) Report numbers of individuals at each stage of study—eg numbers potentially eligible, examined for eligibility, confirmed eligible, included in the study, completing follow-up, and analysed	
		(b) Give reasons for non-participation at each stage	
		(c) Consider use of a flow diagram	
Descriptive Data	14*	(a) Give characteristics of study participants (eg demographic, clinical, social) and information on exposures and potential confounders	
		(b) Indicate number of participants with missing data for each variable of interest	
		(c) *Cohort study*—Summarise follow-up time (eg, average and total amount)	
Outcome Data	15*	*Cohort study*—Report numbers of outcome events or summary measures over time	
		Case-control study—Report numbers in each exposure category, or summary measures of exposure	
		Cross-sectional study—Report numbers of outcome events or summary measures	

For peer review only

Section and Item	Item No.	Recommendation	Reported on Page No.
Main Results	16	(*a*) Give unadjusted estimates and, if applicable, confounder-adjusted estimates and their precision (eg, 95% confidence interval). Make clear which confounders were adjusted for and why they were included	
		(*b*) Report category boundaries when continuous variables were categorized	
		(*c*) If relevant, consider translating estimates of relative risk into absolute risk for a meaningful time period	
Other Analyses	17	Report other analyses done—eg analyses of subgroups and interactions, and sensitivity analyses	
**Discussion**			
Key Results	18	Summarise key results with reference to study objectives	
Limitations	19	Discuss limitations of the study, taking into account sources of potential bias or imprecision. Discuss both direction and magnitude of any potential bias	
Interpretation	20	Give a cautious overall interpretation of results considering objectives, limitations, multiplicity of analyses, results from similar studies, and other relevant evidence	
Generalisability	21	Discuss the generalisability (external validity) of the study results	
**Other Information**			
Funding	22	Give the source of funding and the role of the funders for the present study and, if applicable, for the original study on which the present article is based	

*Give information separately for cases and controls in case-control studies and, if applicable, for exposed and unexposed groups in cohort and cross-sectional studies.

Once you have completed this checklist, please save a copy and upload it as part of your submission. DO NOT include this checklist as part of the main manuscript document. It must be uploaded as a separate file.

For peer review only

BMJ Open

Effect of adherence to spectacle wear on early developing literacy: A longitudinal study based in a large multi-ethnic city, Bradford, UK.

Journal:	*BMJ Open*
Manuscript ID	bmjopen-2017-021277.R2
Article Type:	Research
Date Submitted by the Author:	24-Apr-2018
Complete List of Authors:	Bruce, Alison; Bradford Institute for Health Research<br>Kelly, Brian; Bradford Institute for Health Research<br>Chambers, Bette; University of York, Institute for Effective Education<br>Barrett, Brendan; University of Bradford, School of Optometry and Vision Science<br>Bloj, Marina; University of Bradford, School of Optometry and Vision Science<br>Bradbury, John; Bradford Teaching Hospitals NHS Foundation Trust<br>Sheldon, Trevor; University of York, health Sciences
**Primary Subject Heading**:	Public health
Secondary Subject Heading:	Ophthalmology, Paediatrics, Public health
Keywords:	visual acuity, vision screening, literacy, spectacles, adherence

SCHOLARONE™
Manuscripts

For peer review only

Effect of adherence to spectacle wear on early developing literacy: A longitudinal study based in a large multi-ethnic city, Bradford, UK.

Alison Bruce (corresponding author), Post-doctoral Research Fellow and Head Orthoptist
Bradford Institute for Health Research, Bradford Teaching Hospitals NHS Trust, Duckworth Lane, Bradford, UK BD9 6RJ
01274 383414
alison.bruce@bthft.nhs.uk

Brian Kelly, Statistician
Bradford Institute for Health Research, Bradford Teaching Hospitals NHS Trust, Duckworth Lane, Bradford, UK BD9 6RJ
01274 383466
brian.kelly@bthft.nhs.uk

Bette Chambers, Professor and Director of Institute for Effective Education
University of York, Heslington, York, UK YO10 5DD
bette.chambers@york.ac.uk

Brendan T Barrett, Professor of Visual Development
School of Optometry and Vision Science, University of Bradford, Richmond Rd, Bradford, UK BD7 1DP
01274 235589
B.T.Barrett@bradford.ac.uk

Marina Bloj, Professor of Visual Perception
School of Optometry and Vision Science, University of Bradford, Richmond Rd, Bradford, UK BD7 1DP
01274 236258
M.Bloj@bradford.ac.uk

John Bradbury, Consultant Paediatric Ophthalmologist
Bradford Teaching Hospitals Foundation Trust, Duckworth Lane, Bradford, UK BD9 6RJ
john.bradbury@bthft.nhs.uk

Trevor A Sheldon, Professor of Health Services Research and Policy
Department of Health Sciences, University of York, Heslington, York, UK YO10 5DD
01904 321521
Trevor.Sheldon@york.ac.uk

Word count: 5237

For peer review only

ABSTRACT

Objectives: To determine the impact of adherence to spectacle wear on visual acuity and developing literacy following vision screening at age 4-5 years.

Design: Longitudinal study nested within the Born in Bradford birth cohort.

Setting and participants: Observation of 944 children; 432 had failed vision screening and were referred (treatment group) and 512 randomly selected (comparison group) who had passed (<0.20 logMAR in both eyes). Spectacle wear was observed in school for two years following screening and classified as adherent, (wearing spectacles at each assessment), or non-adherent.

Main outcome measures: Annual measures of visual acuity (VA) using a crowded logMAR Test. Literacy was measured by Woodcock Reading Mastery Tests-Revised subtest: letter identification.

Results: The VA of all children improved with increasing age, -0.009 log units per month (95% CI: -0.011 to -0.007) (worse eye).The VA of the adherent group improved significantly more than the comparison group, by an additional -0.008 log units per month (95% CI: -0.009 to -0.007) (worse eye) and -0.004 log units per month (95% CI: -0.005 to -0.003) in the better eye.

Literacy was associated with the VA, letter-ID reduced by -0.9 (95% CI:-1.15 to -0.64) for every one line (0.10 logMAR) fall in VA (better eye). This association remained after adjustment for socioeconomic and demographic factors (-0.33, 95% CI:-0.54 to -0.12). The adherent group consistently demonstrated higher letter-ID scores compared to the non-adherent group, with the greatest effect size (0.11) in Year 3.

For peer review only

Conclusions: Early literacy is associated with the level of VA; children who adhere to spectacle wear improve their VA and also have the potential to improve literacy. Our results suggest failure to adhere to spectacle wear has implications for the child's vision and education.

For peer review only

For peer review only - http://bmjopen.bmj.com/site/about/guidelines.xhtml

Strengths and limitations of this study

- This is the first longitudinal study to compare the effects of adherence and non-adherence to spectacles in children following vision screening at age 4-5 years on both visual acuity and developing literacy.

- Nesting the study within the Born in Bradford birth cohort allows adjustment for confounding factors.

- The study is observational in nature reflecting real life adherence to spectacle wear.

- The study is not a randomised controlled trial therefore allocation to the adherent or non-adherent groups is not exact and may underestimate the effect of non-adherence.

For peer review only

For peer review only - http://bmjopen.bmj.com/site/about/guidelines.xhtml

INTRODUCTION

Visual development in humans occurs in early-life[1] with the presence of reduced visual acuity (VA) in young children potentially indicating an associated condition such as significant refractive error, strabismus and/or amblyopia.[2] The UK National Screening Committee (UK NSC) recommends visual screening for all children at age 4-5 years,[3] that is in the first year of school, in order to identify a potential reduction in VA. For those who fail the screening test (>0.20 logMAR in one or both eyes)[3] the follow-up clinical pathway includes referral for a cycloplegic refraction and fundus examination to confirm the VA finding and to determine the presence and magnitude of any refractive error and to rule out eye disease.[4] In those with reduced VA, treatment generally consists of the wearing of spectacles[5] and may be combined with occlusion therapy[6] (wearing an eye patch or atropine drops). However, adherence to treatment, both spectacle wear[7,8] and occlusion therapy is known to be variable.[9]

Decreased VA, both near and distance and also the presence of refractive error in young children has been reported to be associated with reduced literacy levels.[10-12] However, there is a paucity of evidence on the impact of non-adherence to spectacle wear on VA and early developing literacy in children. Early literacy skills such as letter recognition,[13] word reading and decoding[14] taught in the first years of school are indicators of future reading performance and educational attainment, which in turn affect long-term health and social outcomes.[15,16] The initial school years are a crucial time for the development of these key literacy skills[17] and it is important to understand the impact of non-adherence to spectacle wear on visual outcome and educational attainment.

Low educational attainment is associated with socioeconomic deprivation,[16] which makes the investigation of the relationship between visual acuity and literacy difficult, as in order to account for potential confounding factors, comprehensive epidemiological data are required. Born in Bradford (BiB) is a large birth cohort, which collected maternal and early-life measures from mothers and their children in Bradford and details of recruitment have been previously reported.[18] By linking separately-collected vision and literacy data in children in

For peer review only - http://bmjopen.bmj.com/site/about/guidelines.xhtml

the BiB cohort, we had the opportunity to explore the association between VA, spectacle wear and literacy development whilst taking into account the effects of potential confounders. The aim of this study is to examine the impact of adherence to spectacle wear on VA and early developing literacy skills in children during their first three years of school.

METHODS

This is a prospective, longitudinal study nested within the BiB cohort following children from the point of their initial vision screening at age 4 -5 years. The study took place between 2012 and 2015. Baseline epidemiological data collected from mothers and children of the BiB cohort, literacy measures, vision screening results and repeat measures of vision and literacy were linked in order to evaluate the longitudinal impact of adherence to spectacle wear on VA and early literacy.

Population

All children invited to join the study were participating in the BIB,[18] a longitudinal, multi-ethnic birth cohort study aiming to examine the impact of environmental, psychological and genetic factors on maternal and child health and wellbeing. Bradford is an ethnically diverse city (approximately half of the births are to mothers of South Asian origin) with high levels of socio-economic deprivation. The cohort is broadly representative of the city's maternal population of child bearing age.

Patient and Public Involvement

The Born in Bradford (BiB) project emphasises the importance of involving parents and ensuring they are central to the research that is prioritised; what is important to the parents, how people find out the results from the research projects, and what it means for their families. The participants were asked their views on many research topics including literacy levels, vision and the impact of vision on literacy. The participants suggested that these topics are of high importance and should be prioritised. The preliminary findings have been

For peer review only - http://bmjopen.bmj.com/site/about/guidelines.xhtml

reported to the parents to provide verification of the data, ensuring that the findings reflect true patient experiences. Their ideas are essential in developing and revising current information provided to parents and carers. Their involvement has allowed the research to be prioritised around the needs and requirements of patients and carers. Finally in the dissemination of the research results the parents will be central to publicising this study and its findings to local people, schools and the wider community.

Recruitment

As part of a BiB study children's literacy levels on school entry (termed 'Reception Class' in England, UK and defined as Year 1 of this study) were measured between September 2012 and July 2014 in Bradford schools. 2930 BiB children from seventy-four of the one hundred and twenty-three primary schools (60%) participated. 432 of the 2930 (14.7%) failed their vision screening (Figure 1) and were referred for follow-up cycloplegic investigation, these children are defined as the treatment group. A further 512 BiB children from the same schools (randomly selected using Excel's random number generator) who had passed vision screening were also invited to participate and were defined as the comparison group, giving a total of 944 participants in the study. Consent was opt-out and parents received a letter via the schools requesting continued participation prior to each annual assessment. 893 of the 944 (94.6%) consented to participate in Year 2 and 650/944 (68.9%) participated in Year 3 (Figure 1).

Baseline Vision Assessments – Year 1

The vision screening programme for 4-5 year old children in Bradford is conducted in the first year of school by orthoptists with 97% of eligible children being screened.[19] The screening includes standard protocols for measurement of monocular distance VA.[20,21] VA was measured at a distance of three metres using the LogMAR Crowded Test (Keeler, Windsor, UK) which has four letters per line, with each letter having a score of 0.025; the total score for each line thus represents 0.10 log unit (Supplementary Information 1). A matching card was used and knowledge of letters was not therefore necessary to perform the test. VA was

For peer review only - http://bmjopen.bmj.com/site/about/guidelines.xhtml

For peer review only

measured to threshold (i.e. best achievable VA with no defined endpoint). In addition cover test at 6m and 1/3m was performed. The data formed the baseline vision data (Year 1). No child in the study was wearing spectacles at the baseline assessment.

Children failing to achieve the VA pass criterion (>0.20 logMAR in one or both eyes) set by the UK National Screening Committee[3] or who had a strabismus detected on cover testing were referred for follow-up. The standard clinical pathway[4] following vision screening entailed referral to either to a community optometrist or the hospital eye service where a cycloplegic refraction (1% cyclopentolate hydrochloride) and fundus examination were undertaken, either by a paediatric ophthalmologist or an optometrist. Spectacles were prescribed based on the result of the cycloplegic refraction and clinical judgement; children were generally prescribed spectacles, including low degrees of hypermetropia (>+1.00DS to +3.00DS), if they had a reduced VA. A follow-up appointment was then arranged with the orthoptist approximately 8 weeks after the cycloplegic examination to repeat the VA measurement, with the child wearing spectacles if they had been prescribed. Children assessed by a community optometrist of their choice had the results of their examination returned to the hospital eye service and also had a follow-up appointment arranged with an orthoptist.

All VA testing, both at the point of vision screening and at follow-up, was performed using the same method of measurement. The results of the follow-up assessment including cycloplegic refraction, VA with the prescribed glasses, cover testing and fundus and media examination were extracted from the medical notes. The ophthalmic staff did not have knowledge of the baseline literacy assessment.

Baseline Literacy Assessments – Year 1

Literacy was measured on school entry (Year 1) by trained research assistants within the same academic term as the vision screening. The research assistants were unaware of the

For peer review only - http://bmjopen.bmj.com/site/about/guidelines.xhtml

VA results. An age-appropriate literacy measure, the Woodcock Reading Mastery Tests-Revised (WRMT-R) subtest: Letter Identification (ID), a validated reading skill test, was used to assess early literacy.[22] Letter identification measures the child's ability to identify single letters, an essential skill mastered prior to reading and one of the best predictors of future reading achievement.[15] The letter-ID test is a test of knowledge of letters (the complete alphabet is used) and the child must verbally identify the name of each letter. This literacy measure specifically uses varied font type; the size of the letters approximate to 1.1 log unit (20/250) at 33cm, therefore the performance on this test is not affected by the level of VA. Letter-ID was collected in both raw and age standardised format. In addition receptive vocabularywas measured using the British Picture Vocabulary Scale (BPVS)[23] an indicator of cognitive ability, providing a representation of IQ in young children. This measure is included to adjust for potential confounding due to levels of general cognitive ability.

Follow up Assessments - Years 2 and 3

Vision and literacy measures were repeated within the same school term approximately 12 months (Year 2) and 24 months (Year 3) after the baseline measurements. Both the vision and the literacy assessments were administered on the same day by the same personnel who were unaware of previous vision or literacy results. VA and literacy was measured as detailed above. VA found to be ≥0.10 logMAR was repeated with a pinhole and near VA was measured using the Bailey-Lovie near-vision chart.[24] (Supplementary Information 1)and whether the child was wearing spectacles was recorded. In order to present the real-life impact of adherence to spectacle wear, all VA measures reported are presenting visual acuities i.e. measured with spectacles if worn at the time of the assessment in school. Parents and children were not given prior warning of these assessments. This study was approved by National Research Ethics Committee Yorkshire & the Humber- South Yorkshire (Ref 13/YH/0379).

For peer review only - http://bmjopen.bmj.com/site/about/guidelines.xhtml

Statistical Analysis

Children with baseline data for both vision and literacy in Year 1 and who had at least one follow-up measure in either Year 2 or Year 3 were included in the final analysis (Figure 1). The statistical model selected for the analyses, using projections over time, takes into account missing data and requires a minimum of measures at two time points. Using this type of statistical analysis allows inclusion of a greater number of participants giving maximum power to the analyses.[25] The characteristics of children participating in the study were compared initially using Chi-squared or two sided t-tests as appropriate. Children in the treatment group were retrospectively divided into two sub-groups, adherent and non-adherent. Adherence was defined as wearing prescribed spectacles at the time of assessment; otherwise children were defined as non-adherent. Children who were assessed twice but only wore the spectacles on one occasion were classed as non-adherent. A sensitivity analysis was conducted to assess the extent to which the results varied by changing the definition of adherence.

Analysis of Visual Acuity

To investigate the effect of spectacle wear over time on VA, multilevel longitudinal models[25] were firstly constructed with VA as the outcome measure for the child's better and worse eye. The models measure change within the individual and change between individuals over time and allow for individual differences in the rate of change over time.[25] A quadratic term was included to model the non-linear trajectory of change. The model also includes an interaction term to compare the relationship between age and group, to test whether differences by group are the same at different ages. Unadjusted analysis was initially undertaken with subsequent adjustment for demographic and socioeconomic factors reported in the literature to be associated with reduced VA: early-life factors[26] (gender, gestational age, birth weight, route of birth) and maternal factors[27] (ethnicity, mother's age at delivery, mother's level of educational attainment and being in receipt of means-tested

For peer review only - http://bmjopen.bmj.com/site/about/guidelines.xhtml

benefits). Predicted outcomes were plotted to visualise group differences and change in the outcomes for each group over time.

Analysis of Literacy

In order to estimate the association between the letter-ID and VA the same multilevel and longitudinal modelling approach was adopted, but with the final letter-ID score as the outcome measure. The raw letter-ID scores were used in the analysis in order to explore change over time. After estimating differences between the groups and accounting for the initial letter-ID at baseline (Year 1), further adjustment was undertaken for the factors reported in the literature to be associated with educational attainment,[28 ,29] the early-life factors and maternal factors as stated above. Spherical equivalent refraction (SER) (sphere plus half cylinder) of the better eye was included as was BPVS score in order to account for cognitive ability. The results of these models are presented along with predicted outcomes for each of the groups. Effect sizes are generally reported when appraising educational interventions. To demonstrate group differences at each time point effect sizes were calculated for the letter-ID scores using Cohen's *d*.[30]

Visual Acuity – Year 3.

Children were unable to accurately perform the near VA (logMAR) test until Year 3; we are therefore unable to provide a longitudinal analysis. In Year 3 we have measures of both near VA and distance VA and present the correlation between the near and distance VA at this time point only. Additionally we analysed association between near VA and literacy to examine if the results differed from the association between distance VA and literacyin Year 3 only.

All analyses were carried out using Stata V.13 (StataCorp, College Station, Texas, USA).

For peer review only - http://bmjopen.bmj.com/site/about/guidelines.xhtml

RESULTS

Data from 801 (85%) children from 67 schools were included in the final analysis (Figure 1). Twelve children in the treatment group were excluded from the analysis as they had ocular conditions other than refractive error (e.g. nystagmus) confirmed in their medical notes, leaving 368 children in the treatment group and 433 in the comparison group. 230/368 (62.5%) of children in the treatment group had attended for the initial cycloplegic examination and been prescribed spectacles, 3/368 (0.8%) attended but no cycloplegic refraction information was available, 23/368 (6.3%) had been prescribed spectacles but had not returned for follow-up VA assessment, and 112/368 (30.4%) had failed to attend any appointment following vision screening. Of the 253 children in the treatment group with cycloplegic refraction results, 157/253 (62.1%) had astigmatism (>1.00DC) either alone (n=19) or in combination with hypermetropia (>+3.0DS) (n=56), low hypermetropia (>+1.0DS to +3.0DS) (n=16) or myopia (≤-0.50DS) (n=66). 35/253 (13.8%) had hypermetropia alone, 11 (4.3%) had myopia alone and 50 (19.8%) children had low hypermetropia . 55 of 253 (21.7%) additionally had anisometropia (≥1.0D difference). For those children with a cycloplegic refraction result (Table 1) the SER ranged from -7.875 to +7.50D in the better eye and -8.25 to +7.50D in the worse eye. Fourteen of the 368 (3.8%) children had a constant or intermittent strabismus, five of whom had been prescribed occlusion therapy for amblyopia at follow-up after vision screening. Those children were not excluded from the analysis as they met the initial VA referral criteria and had been prescribed spectacles. Baseline (Year 1) characteristics of the children in the comparison and treatment groups are shown in Table 1. A small mean difference (-0.021 logMAR, 95% CI -0.022 to -0.020) in VA between the eyes of the comparison group was found, equating to one letter difference. This is not clinically significant but is statistically significant therefore VAs are presented for the better and worse eye separately. Higher levels of VA were found in both eyes of the comparison group compared to the treatment group (Chi-squared p<0.001) (Table 1). The only demographic factor found to differ between the comparison and the treatment group

For peer review only - http://bmjopen.bmj.com/site/about/guidelines.xhtml

was the average mother's age which was around 10 months more in the treatment group (Chi-squared p<0.001).

Table 1. Characteristics of Born in Bradford children and mothers included in the analyses. Values are numbers (%) or mean (SD).

	Comparison group n=433	Treatment group n=368	P value†
**Children**			
Age (months) Year 1	60 (4.2)	60 (4.5)	0.119
Gender			
Male	229 (51.1)	183 (49.7)	
Female	219 (48.9)	185 (50.3)	0.693
Ethnicity			
White	125 (28.0)	91 (24.9)	
Pakistani	262 (58.7)	232 (63.4)	
Other	59 (13.3)	43 (11.7)	0.403
Route of birth			
Vaginal	342 (77.0)	291 (79.7)	
Caesarean	102 (23.0)	74 (20.3)	0.355
Gestational age at birth (weeks)	277 (12.0)	276 (13.0)	0.158
Birth weight (g)	3184 (550.0)	3128 (573.0)	0.155
VA better eye	0.113 (0.049)	0.271 (0.138)	<0.001
VA worse eye	0.135 (0.046)	0.428 (0.189)	<0.001
SER better eye ‡	-	1.19 (0.95)	-
SER worse eye ‡	-	1.98 (1.27)	-
**Mother**			
Age (years)	27.3 (5.4)	28.1 (5.7)	<0.001
Mother's education			
<A-level	227 (64.5)	190 (69.3)	
A-level or above	125 (35.5)	84 (31.7)	0.201
In receipt of means tested benefits (yes)	163 (45.0)	144 (50.1)	0.139

†Difference between Comparison and treatment groups (chi-squared or t-test as appropriate).
VA = visual acuity. VA's are measured in logMAR; therefore higher values represent poorer VA.
SER= spherical equivalent refraction. ‡Cycloplegic results were available for the treatment group only.

Table 2 presents the baseline (Year 1) characteristics of those children in the treatment group retrospectively categorised as adherent (173/368, 47.0%) and non-adherent (195/368, 53.0%) (Figure1). In the non-adherent group, no child wore spectacles at their Year 2 assessment and 39/195 (20%) wore them in Year 3 only. At baseline, the group subsequently classed as adherent had a lower level of VA compared to the non-adherent group in both the better and worse eye (Table 2). The only other factor that differed between the adherent and the non-adherent groups was the mother's level of education with 50/173

For peer review only - http://bmjopen.bmj.com/site/about/guidelines.xhtml

(39.1%) of adherent children having mothers educated to A-level or above compared to only 34/195 (23.3%) of the non-adherent group (Chi-squared p=0.005). BPVS did not differ between the adherent and non-adherent children (p=0.553) suggesting no difference in cognitive ability.

Table 2. Baseline characteristics of participants in the treatment group retrospectively classed as adherent and non-adherent. Values are numbers (%) or mean (SD).

	Adherent n=173 (47.0%)	Non-adherent n=195 (53.0%)	P value†
**Children**			
Age (months) Year 1	59.4 (4.5)	59.6 (4.5)	0.850
Gender			
Male	81 (46.8)	102 (52.3)	
Female	92 (53.2)	93 (47.7)	0.293
Ethnicity			
White	48 (27.9)	43 (22.2)	
Pakistani	103 (59.9)	129 (66.5)	
Other	21 (12.2)	22 (11.3)	0.387
Route of birth			
Vaginal	137 (79.6)	154 (79.8)	
Caesarean	35 (20.4)	39 (20.2)	0.973
Gestational age at birth (weeks)	276 (13.0)	275 (14.0)	0.383
Birth weight (g)	3121 (569.0)	3134 (579.0)	0.833
VA better eye‡	0.292 (0.150)	0.256 (0.129)	0.008
VA worse eye‡	0.465 (0.197)	0.399 (0.175)	0.001
SER better eye	1.18 (0.86)	1.20 (1.02)	0.960
SER worse eye	2.02 (1.20)	1.96 (1.33)	0.657
Language ability scores§	97.8 (15.6)	96.8 (16.4)	0.553
**Mother**			
Age (years)	28.1 (5.8)	28.0 (5.7)	0.845
Mother's education			
<A-level	78 (60.9)	112 (76.7)	
A-level or above	50 (39.1)	34 (23.3)	0.005
In receipt of means tested benefits (yes)	61 (45.5)	83 (55.7)	0.087

†Difference between Adherent and non-adherent treatment groups (chi-squared or t-test as appropriate).
VA = visual acuity. VA's are measured in logMAR; therefore higher values represent poorer VA.
SER= spherical equivalent refraction. §Age-adjusted language ability measure for British Picture Vocabulary Score (BPVS).
‡No child was wearing spectacles at the baseline assessment.

Visual Acuity

At baseline compared to the comparison group both the adherent (mean difference: 0.337 logMAR; 95% CI: 0.304 to 0.370) and non-adherent groups (mean difference: 0.273 logMAR; 95% CI: 0.241 to 0.305) had lower levels of VA in the worse eye. Table 3 and Figure 2 present the VA trajectories over the course of the study. These show that after

For peer review only - http://bmjopen.bmj.com/site/about/guidelines.xhtml
For peer review only

adjusting for previously described early-life and maternal variables, the VA of both eyes for all three groups; the comparison, the adherent and the non-adherent groups improve over time.

The VA of all children improved with increasing age, -0.009 log units per month (95% CI: -0.011 to -0.007) (worse eye) and -0.006 log units per month (-0.008 to -0.005) (better eye) (Table 3).

Over and above this improvement the adherent group (worse eye) improved by a further -0.008 log units per month (95% CI: -0.009 to -0.007). The adherent children therefore improved overall by -0.017 (95% CI -0.020 to – 0.015) log units per month (95% CI: -0.009 to-0.007) (approximately two letters every 3 months) and also demonstrated a small amount of improvement in the better eye above that expected from age (Table 3).

The non-adherent group (worse eye) improved by -0.003 log units per month (95% CI: -0.004 to -0.001) above that expected from age. The non-adherent children therefore improved overall by -0.012 log units per month (95% CI: -0.014 to -0.010). No additional improvement above that expected from age was demonstrated in the better eye (Table 3).

Sensitivity analysis redefining the classification of adherence did not materially affect the results.

For peer review only - http://bmjopen.bmj.com/site/about/guidelines.xhtml

Table 3. Change in visual acuity for the better and worse eye over time by group; comparison, adherent and non-adherent.

	**Unadjusted (worse eye) (95% CI)**	**Adjusted‡ (worse eye) (95% CI)**	**Unadjusted (better eye) (95% CI)**	**Adjusted‡ (better eye) (95% CI)**
Constant	0.177 (0.159 to 0.194)*	0.386 (0.124 to 0.648)*	0.240 (0.026 to 0.454)*	0.240 (0.026 to 0.454)*
Age (months)	-0.009 (-0.011 to -0.008)***	-0.009 (-0.011 to -0.007)***	-0.006 (-0.008 to -0.005)***	-0.006 (-0.008 to -0.005)***
Age (months) squared	0.00016 (0.00012 to 0.00020)***	0.00016 (0.00012 to 0.00021)***	0.00010 (0.00007 to 0.00013)***	0.00010 (0.00006 to 0.00014)***
Group (reference: comparison)				
Adherent	0.337 (0.309 to 0.366)***	0.337 (0.304 to 0.370)***	0.184 (0.162 to 0.106)***	0.170 (0.144 to 0.196)***
Non-adherent	0.277 (0.250 to 0.305)***	0.273 (0.241 to 0.305)***	0.150 (0.128 to 0.172)***	0.148 (0.123 to 0.174)***
Age ^{X} Group interaction				
Age ^{X} adherent	-0.008 (-0.009 to -0.007)***	-0.008 (-0.009 to -0.007)***	-0.004 (-0.005 to -0.004)***	-0.004 (-0.005 to -0.003)***
Age ^{X} non-adherent	-0.003 (-0.003 to -0.001)***	-0.003 (-0.004 to -0.001)***	-0.001 (-0.002 to 0.000)	-0.002 (-0.004 to 0.000)

‡Model adjusted for gender, ethnicity, gestation period, birthweight, birth route, maternal education status, maternal age and means-tested benefit status.
^{X} Interaction between group and age to determine if the effect of being in a particular group changes with age. The total effect for any one group is the coefficient for age **plus** the additional effect of age for that group.
*p < 0.05, **p < 0.01, ***p< 0.001

For peer review only - http://bmjopen.bmj.com/site/about/guidelines.xhtml

Table 4. Associations between Letter-ID score, visual acuity (better eye), maternal and early-life factors.

FACTOR	UNADJUSTED MODEL (95% CI)	p value	FULLY ADJUSTED MODEL (95% CI)	p value
Constant	18.82 (17.91 to 19.73)	<0.001	-20.6 (-28.2 to -13.0)	<0.001
Age	1.30 (1.21 to 1.38)	<0.001	1.28 (1.19 to 1.37)	<0.001
Age squared	-0.02 (-0.02 to -0.02)	<0.001	-0.020 (-0.022 to -0.017)	<0.001
Visual Acuity: change in Letter-ID per 0.1log unit (one line)	-0.90 (-1.15 to -0.64)	<0.001	-0.327 (-0.540 to -0.115)	0.003
Letter ID baseline (Year 1)			0.348 (0.326 to 0.371)	<0.001
BPVS			0.019 (-0.001 to 0.039)	0.064
Ethnicity				
Pakistani heritage			0.668 (-0.016 to 1.353)	0.056
Other			1.174 (1.159 to 2.189)	0.023
Gender				
Female			0.471 (-0.093 to 1.035)	0.102
Birth weight (per 100g)			0.074 (0.008 to 0.141)	0.029
Gestational age (weeks)			-0.053 (-0.257 to 0.151)	0.611
Receiving Benefits			-0.086 (-0.661 to 0.4990)	0.770
Mothers Level of Education	(higher than A-level)		0.765 (0.156 to 1.374)	0.014
Mothers age at birth (years)			-0.048 (-0.100 to 0.005)	0.075

BPVS = British Picture Vocabulary Scale (baseline standardised score).

For peer review only

Literacy

The unadjusted model shows the final letter-ID score reduces by -0.9 units (95% CI:-1.15 to -0.64) for every one line (0.10 logMAR) fall in VA of the better eye (Table 4). This association persists but is weaker after fully adjusting for the socioeconomic and demographic factors, the letter-ID score declines by -0.327 units (95% CI:-0.540 to -0.115) for every one line fall in VA. Separate adjusted analysis of the VA level of the worse eye shows similar results but with weaker association, letter-ID score declines by -0.260 units (95% CI:-0.414 to -0.105) for every one line fall in VA.

Children of mothers educated to A-level or above had increased letter-ID scores (0.765 units; 95% CI: 0.156 to 1.374) compared to those with lower qualifications. Ethnicity other than white British or Pakistani heritage was associated with better letter-ID score, which might reflect the higher number of mothers educated to above A-level in this group. Greater birth weight was also associated with increased letter-ID score (Table 4). Adjustment for SER made no difference and was not associated with letter-ID (p=0.306). It was therefore

For peer review only - http://bmjopen.bmj.com/site/about/guidelines.xhtml

not included in the models. Similarly subsequent analysis replacing SER with refractive error categories did not show an association with letter-ID (Supplementary Information 2).

A predictive model of the letter-ID score over time for children in each group (Figure 3) was constructed using both the unadjusted and adjusted data from the VA trajectories (Table 3) and incorporated into the model reporting letter-ID (Table 4). The unadjusted trajectory shows both adherent and non-adherent groups at baseline have lower letter-ID scores than the comparison group. The predicted trajectory of improvement in the adherent group is greater than the non-adherent group with the later letter-ID scores of the adherent group converging on those of the comparison group by Year 3. The non-adherent group although improving over time does not catch up with the adherent or the comparison groups. After adjusting for socio-economic and demographic variables the trend is similar but with a smaller difference between the groups.

Table 5 presents the effect size of wearing spectacles on the letter-ID scores between the groups annually over the three years of the study. Comparing the letter-ID scores between the adherent and the non-adherent group a gradual increase in the effect size over time is demonstrated with the greatest effect size (0.11) between the adherent and non-adherent groups shown in Year 3.

Visual Acuity – Year 3

The results demonstrate a statistically significant correlation between near and distance visual acuity in Year 3 (Right Eye r =0.663 and Left Eye r = 0.642) (Supplementary Information 3). In addition the association between the near VA and literacy score and distance VA and literacy score are approximately the same (Supplementary Information 4).

For peer review only - http://bmjopen.bmj.com/site/about/guidelines.xhtml

Table 5. Annual Literacy Scores by Group.

Year	Group	Letter-ID (raw score)	Comparison Groups	Effect Size (Cohen's *d*)*
1	Comparison	25	Comparison v Adherent	0.06
	Adherent	24.3	Comparison v Non-adherent	0.06
	Non-adherent	24.3	Adherent v Non-Adherent	0.00†
2	Comparison	34.7	Comparison v Adherent	0.05
	Adherent	34.4	Comparison v Non-adherent	0.13
	Non-adherent	34.0	Adherent v Non-Adherent	0.07
3	Comparison	39.1	Comparison v Adherent	0.08
	Adherent	38.8	Comparison v Non-adherent	0.18
	Non-adherent	38.4	Adherent v Non-Adherent	0.11

*Based on group difference divided by the pooled standard deviation (SD) of Letter-ID score: SD 10.9 at Year 1, 5.6 at Year 2 and 3.8 at Year 3.
† In Year 1 there is no difference as spectacle wear has not commenced.

DISCUSSION

This is the first longitudinal study to assess the effect of adherence/non-adherence to spectacle wear on VA and literacy in children following vision screening. The VA of children who adhered to spectacle wear was found to improve at a far greater rate compared to those who were non-adherent, with the VA of adherent children reaching similar levels to the VA of the comparison children by the end of the study. Our results further indicate that early developing literacy is affected by the level of VA even after adjusting for socio-economic and demographic factors associated with educational attainment. The letter-ID score declines by approximately 1.5% for every one line of reduction in VA. In this and similar populations [14,31], where children have been reported to have reduced VA levels (>0.30logMAR in better eye), there is likely to be an impact on developing literacy skills. The effect size (0.11) of being adherent to spectacle wear compared to non-adherence in Year 3 of our study is the same as that reported in a Chinese study providing free spectacles to children[32] and is comparable with reported educational interventions.[33] Thus children who fail vision screening and adhere to spectacle wear have the potential to improve their VA, further influencing early literacy development.

For peer review only - http://bmjopen.bmj.com/site/about/guidelines.xhtml

Adherence to spectacle wear is highly influenced by socio-economic and demographic factors, particularly maternal education, a factor that is also known to be associated with educational attainment.[34] Children with reduced VA and who are in less educated families are less likely to adhere to treatment, which will further impact on their educational attainment and future life chances. We were however, able to adjust for the many associated maternal and early-years factors, the value of embedding this study within a birth cohort. A study examining academic performance in US schools reports that failing vision screening was predictive of being in the lowest quartile of academic performance.[35] Conversely, a longitudinal study of children aged 9 – 10 years in Singapore, Dirani et al[36] found VA did not play a significant role in predicting academic performance. The children were however older, mainly myopic and only a small number of participants had decreased VA which may account for the difference in their findings relative to ours.

The VA of children in all groups (adherent, non-adherent and comparison group) continued to improve throughout this study. The improvement in VA found in the comparison group is similar to that reported for normal visual development, with optimum VA achieved around 6 years of age.[37,38] The improvement in VA of the worse eye found in adherent children over the time of the study was significantly greater than that expected solely from visual development[39] or indeed from retest variability[40] and was almost double that of the comparison group. Little additional improvement above that expected from visual development was demonstrated in the worse eye of the non-adherent children, an indication that the improvement in the adherent children is not due to regression to the mean. The longitudinal observation of the children demonstrates improvement not only in VA but also in literacy, with the non-adherent group demonstrating persistently lower literacy scores throughout the study, although the effect is attenuated after adjusting for other factors.

Annual improvement in academic achievement is well recognised and is particularly notable in the early years of schooling with the initial improvement thought to be associated with the effect of entering school, combined with rapid early child development followed by a plateau in academic growth as children progress through school grades.[20]

For peer review only - http://bmjopen.bmj.com/site/about/guidelines.xhtml

Early literacy development is complex and associated with socio-economic and demographic factors, in particular maternal education. However, even after taking these factors into account VA continues to be associated with literacy; the poorer the level of VA, the greater the reduction in the literacy score. In a Singaporean study,[39] a strong association between paternal level of education and academic school performance was reported. As one might expect, higher levels of maternal education have a positive impact on literacy. [41,42] In addition, mothers with higher educational attainment are more likely to effectively access health services, and are more likely to adhere to prescribed treatment.[43]

Our study shows an association between VA and literacy score but no association between SER and literacy. Neither did further analysis by refractive error types indicate an association with literacy, this is most likely related to a lack of power due to the small numbers when refractive error is categorised in our study. Our findings differ from previous studies reporting an association between refractive error and literacy.[11,12]

Hypermetropia has been reported to be associated with poor literacy. A large cross-sectional American study (VIP-HIP) of pre-school children aged 4-5 years found that children with uncorrected hypermetropia in conjunction with reduced binocular near VA (worse than 20/40) have poorer literacy than those with hypermetropia and a good level of binocular near visual acuity.[12] The VIP-HIP study report that the level of binocular near VA was predictive of literacy scores; with hypermetropic children with binocular near VA better than 20/40, demonstrating literacy scores similar to those children who were emmetropic. Although the VIP-HIP study does not report distance VA levels of the children it does state that the analysis of the distance VA resulted in similar findings, an indication that distance VA levels may also influence early literacy scores.

Astigmatism has also been reported to be associated with reduced literacy. In native American children bilateral uncorrected astigmatism (≥ 1.00 DC) has been reported to reduce reading fluency, and children with moderate astigmatism are reported to have lower VA and fluency than those with no or low astigmatism.[11] The findings reported from both the

For peer review only - http://bmjopen.bmj.com/site/about/guidelines.xhtml

above studies may indicate that moderate to high degrees of uncorrected hypermetropia or astigmatism which reduce VA is associated with a reduction in literacy scores.

Classroom based tasks where fixation frequently changes are reported to require high levels of distance VA (0.33logMAR) and slightly lesser levels of near VA (0.72logMAR)[44] this is most probably due to print size for early readers being enlarged. We would suggest therefore that where VA is reduced beyond that required in the learning environment it will impact on a child's developing literacy and hence the association we report between distance VA and literacy.

The longitudinal design of this study provides an insight into development of VA and literacy in the early years of schooling, and the use of linked data from the mothers and children participating in the BiB cohort study permitted the many potential confounding factors associated with educational attainment to be accounted for. We include children with a wide range of refractive error and VAs allowing a robust analysis of the influence of both factors on developing literacy. The study does however have some weaknesses. It is not a randomised controlled trial and non-adherence was defined retrospectively by the failure of the child to wear their prescribed glasses at one assessment; it is possible that this was a unique event and is not representative of the child's true adherence to spectacle wear over the course of the study. If this is indeed the case, then the random misclassification is likely to under-estimate the difference found between the adherent and non-adherent groups.[45] In addition the sensitivity analysis redefining non-adherence does not demonstrate any material difference in the results.

A cycloplegic examination was not undertaken for all children and there will be some children with reduced vision who were not identified at screening (false negatives). No child who had a cycloplegic refraction was found to be a false positive but a proportion of the children who failed to attend for the cycloplegic examination may be false positives. This misclassification will similarly be random, underestimating the size of estimates of effect and suggests our estimates may be conservative.[45]

For peer review only - http://bmjopen.bmj.com/site/about/guidelines.xhtml

For peer review only

Visual acuity is the sole measure of visual function reported from the study and it is possible other measures of visual function are also associated with academic performance; further research would be required to explore these associations. The VA assessment and the literacy test are both letter based and children who struggle with letter identification may also demonstrate a poor ability with the VA test. However, all children used a matching technique, a skill that is present in children as young as three years[46] and no child who failed the screening was classed as false positive.

During visual maturation, the presence of neurodevelopmental disorders such as refractive error, and strabismus may contribute to a reduction in VA and early intervention is required. This study demonstrates that wearing spectacles is an effective intervention to improve VA, and that this will impact positively on developing literacy. The children who do not adhere to spectacle wear are likely to be those in families who are less well educated. Further research is required to better understand the reasons for non-adherence and evaluate interventions to promote adherence to spectacle wear. This has the potential not only to improve vision but also support future life chances in children who may already face educational disadvantage.

For peer review only

For peer review only - http://bmjopen.bmj.com/site/about/guidelines.xhtml

REFERENCES

1. Daw NW. Critical periods and amblyopia. *Arch Ophthalmol* 1998;116(4):502-5.

2. Robaei D, Rose K, Ojaimi E*, et al.* Visual Acuity and the Causes of Visual Loss in a Population-Based Sample of 6-Year-Old Australian Children. *Ophthalmology* 2005;112(7):1275-82.

3. National Screening Committee. The UK NSC policy on Vision defects screening in children 2013. Available: http://www.screening.nhs.uk/vision-child (Accessed 19/12/2017).

4. Public Health England. Child vision screening resources consultation. Available: https://www.gov.uk/government/consultations/child-vision-screening-resources (Accessed 04/08/2017). 2017.

5. Stewart CE, Moseley MJ, Fielder AR*, et al.* Refractive adaptation in amblyopia: quantification of effect and implications for practice. *Br J Ophthalmol* 2004;88(12):1552-6.

6. Pediatric Eye Disease Investigator G, Repka MX, Kraker RT*, et al.* A randomized trial of atropine vs patching for treatment of moderate amblyopia: follow-up at age 10 years. *Arch Ophthalmol* 2008;126(8):1039-44.

7. Aldebasi YH. A descriptive study on compliance of spectacle-wear in children of primary schools at Qassim Province, Saudi Arabia. *Int J Health Sci (Qassim)* 2013;7(3):291-99.

8. Sharma A, Congdon N, Patel M*, et al.* School-based approaches to the correction of refractive error in children. *Surv Ophthalmol* 2012;57(3):272-83.

9. Maconachie GDE, Gottlob I. The challenges of amblyopia treatment. *Biomed J* 2015;38(6):510-16.

10. Bruce A, Fairley L, Chambers B*, et al.* Impact of visual acuity on developing literacy at age 4-5 years: a cohort-nested cross-sectional study. *BMJ Open* 2016;6(2):e010434.

11. Harvey EM, Miller JM, Twelker JD*, et al.* Reading Fluency in School-Aged Children with Bilateral Astigmatism. *Optom Vis Sci* 2016;93(2):118-25.

12. VIP-HIP Study Group, Kulp MT, Ciner E*, et al.* Uncorrected Hyperopia and Preschool Early Literacy: Results of the Vision in Preschoolers-Hyperopia in Preschoolers (VIP-HIP) Study. *Ophthalmol* 2016;123(4):681-9.

For peer review only - http://bmjopen.bmj.com/site/about/guidelines.xhtml

13. Foulin JN. Why is letter-name knowledge such a good predictor of learning to read? *Read Writ* 2005;18(2):129-55.

14. Marchman VA, Fernald A. Speed of word recognition and vocabulary knowledge in infancy predict cognitive and language outcomes in later childhood. *Dev Sci* 2008;11(3):9-16.

15. Dearden L, Sibieta L, Sylva K. The socio-economic gradient in early child outcomes: evidence from the Millennium Cohort Study. *Longit Life Course Stud* 2011;2(1):19 - 40.

16. Marmot M. Fair Society, Healthy Lives.The Marmot review Executive Summary. London: The Marmot Review 2010.

17. Bloom HS, Hill CJ, Black AR*, et al.* Performance Trajectories and Performance Gaps as Achievement Effect-Size Benchmarks for Educational Interventions. *J Res on Educ Eff* 2008;1(4):289-328.

18. Wright J, Small N, Raynor P*, et al.* Cohort Profile: The Born in Bradford multi-ethnic family cohort study. *Int J Epidemiol* 2013;42(4):978-91.

19. Bruce A, Outhwaite L. Uptake, referral and attendance: results from an inner city school based vision screening programme. *Br Ir Orthopt J* 2013;10:41 - 45.

20. McGraw PV, Winn B, Gray LS*, et al.* Improving the reliability of visual acuity measures in young children. *Ophthalmic Physiol Opt* 2000;20(3):173-84.

21. Simmers AJ, Gray LS, Spowart K. Screening for amblyopia: a comparison of paediatric letter tests. *Br J Ophthalmol* 1997;81(6):465-69.

22. Woodcock RW. Woodcock Reading Mastery Tests (Revised) Circle Pines, MN: American Guidance Service, 1987.

23. Dunn LM, Dunn LM, Whetton C*, et al.* British Picture Vocabulary Scale. 2nd edition Windsor, Berks: NFER-Nelson, 1997.

24. Bailey IL, Lovie JE. The design and use of a new near-vision chart. *Am J Optom Physiol Opt* 1980;57(6):378-87.

25. Goldstein H. Models for Repeated Measures Data. Multilevel Statistical Models: John Wiley & Sons, Ltd, 2010:147-60.

For peer review only - http://bmjopen.bmj.com/site/about/guidelines.xhtml

26. Williams C, Northstone K, Howard M*, et al.* Prevalence and risk factors for common vision problems in children: data from the ALSPAC study. *Br J Ophthalmol* 2008;92(7):959-64.

27. Tarczy-Hornoch K, Varma R, Cotter SA*, et al.* Joint Writing Committee for the Multi-Ethnic Pediatric Eye Disease Study the Baltimore Pediatric Eye Disease Study Groups. Risk Factors for Decreased Visual Acuity in Preschool Children: The Multi-Ethnic Pediatric Eye Disease and Baltimore Pediatric Eye Disease Studies. *Ophthalmology* 2011;118(11):2262-73.

28. Burroughs-Lange SG, Douetil J. Literacy progress of young children from poor urban settings: A Reading Recovery comparison study. *Literacy Teaching and Learning* 2007;12(1):19-46.

29. Schoon I, Parsons S, Sacker A*, et al.* Socioeconomic adversity, educational resilience and subsequent levels of adult adaptation. *J Adolesc Res* 2004;19:383-404.

30. Cohen J. *Statistical power analysis for the behavioral sciences (2nd ed). Mahwah, NJ*, 1988.

31. Murthy GV, Gupta SK, Ellwein LB*, et al.* Refractive error in children in an urban population in New Delhi. *Invest Ophthalmol Vis Sci* 2002;43.

32. Ma X, Zhou Z, Yi H*, et al.* Effect of providing free glasses on children's educational outcomes in China: cluster randomized controlled trial. *BMJ* 2014;349.

33. McEwan PJ. Improving Learning in Primary Schools of Developing Countries:A Meta-Analysis of Randomized Experiments. *Rev Educ Res* 2015;85(3):353-94.

34. Neuman S. Lessons from my mother: Reflections on the National Early Literacy Report. *Educ Res* 2010;39(4):301 -04.

35. Krumholtz I. Results from a pediatric vision screening and its ability to predict academic performance. *Optometry (St Louis, Mo)* 2000;71(7):426-30.

36. Dirani M, Zhang X, Goh LK*, et al.* The Role of Vision in Academic School Performance. *Ophthalmic Epidemiol* 2010;17(1):18-24.

For peer review only - http://bmjopen.bmj.com/site/about/guidelines.xhtml

37. Leone JF, Mitchell P, Kifley A, *et al.* Normative visual acuity in infants and preschool-aged children in Sydney. *Acta Ophthalmol* 2014;92(7):e521-e29.

38. Pan Y, Tarczy-Hornoch K, Susan A C, *et al.* Visual Acuity Norms in Preschool Children: The Multi-Ethnic Pediatric Eye Disease Study. *Optom Vis Sci* 2009;86(6):607-12.

39. Sonksen PM, Wade AM, Proffitt R, *et al.* The Sonksen logMAR test of visual acuity: II. Age norms from 2 years 9 months to 8 years. *J AAPOS* 2008;12(1):18-22.

40. Stewart C. Comparison of Snellen and log-based acuity scores for school-aged children. *Br Orthopt J* 2000;57:32-38.

41. Magnuson K. Maternal education and children's academic achievement during middle childhood. *Dev Psychol* 2007;43(6):1497-512.

42. Taylor J, Ennis CR, Hart SA, *et al.* Home environmental and behavioral risk indices for reading achievement. *Learn Individ Differ* 2017;57:9-21.

43. Leurer MD. Perceived Barriers to Program Participation Experienced by Disadvantaged Families. *Int J Health Promot Educ* 2011;49(2):53-59.

44. Narayanasamy S, Vincent SJ, Sampson GP, *et al.* Visual demands in modern Australian primary school classrooms. *Clin Exp Optom* 2016;99(3):233-40.

45. Flegal KM, Brownie C, Haas JD. The effects of exposure misclassification on estimates of relative risk. *American journal of epidemiology* 1986;123(4):736-51.

46. Deák GO, Ray SD, Pick AD. Matching and naming objects by shape or function: Age and context effects in preschool children. *Dev Psychol* 2002;38(4):503-18.

For peer review only - http://bmjopen.bmj.com/site/about/guidelines.xhtml

Funding: AB is funded by a National Institute for Health Research Post-Doctoral Fellowship Award (PDF-2013-06-050). The Born in Bradford study presents independent research commissioned by the National Institute for Health Research Collaboration for Applied Health Research and Care (NIHR CLAHRC) and the Programme Grants for Applied Research funding scheme (RP-PG-0407-10044). The views expressed are those of the author(s) and not necessarily those of the NHS, the NIHR or the Department of Health.

Competing interest statement: "All authors have completed the Unified Competing Interest form and declare: no support from any organisation for the submitted work; no financial relationships with any organisations that might have an interest in the submitted work in the previous three years, no other relationships or activities that could appear to have influenced the submitted work.

Acknowledgments: We thank all the families and schools who took part in this study, the orthoptists from Bradford Teaching Hospitals Foundation Trust who conducted the vision screening programme, the researchers from the Starting Schools programme who collected the literacy measures, Patrick Friis, Alexandra Morris and Hannah Farrugia who collected follow-up measures and the Data Support Team from Bradford Institute for Health Research who created and maintain the data linkage system.

Contributors: AB initiated the project, designed data collection, monitored data collection for the whole study, wrote the statistical analysis plan, cleaned and analysed the data, and drafted and revised the paper. She is guarantor. BK wrote the statistical analysis plan, cleaned the data and revised the draft paper. BC initiated the project and revised the draft paper. BTB contributed to the design of the study and revised the draft paper. MB contributed to the design of the study and revised the draft paper. JB contributed to the design of the study and revised the draft paper. TAS initiated the project, wrote the statistical analysis plan and revised the draft paper.

For peer review only - http://bmjopen.bmj.com/site/about/guidelines.xhtml

“The Corresponding Author has the right to grant on behalf of all authors and does grant on behalf of all authors, a worldwide licence to the Publishers and its licensees in perpetuity, in all forms, formats and media (whether known now or created in the future), to i) publish, reproduce, distribute, display and store the Contribution, ii) translate the Contribution into other languages, create adaptations, reprints, include within collections and create summaries, extracts and/or, abstracts of the Contribution, iii) create any other derivative work(s) based on the Contribution, iv) to exploit all subsidiary rights in the Contribution, v) the inclusion of electronic links from the Contribution to third party material where-ever it may be located; and, vi) licence any third party to do any or all of the above.”

"The Corresponding Author has the right to grant on behalf of all authors and does grant on behalf of all authors, an exclusive licence on a worldwide basis to the BMJ Publishing Group Ltd to permit this article (if accepted) to be published in BMJ editions and any other BMJPGL products and sublicences such use and exploit all subsidiary rights, as set out in our licence."

Transparency declaration: Dr Alison Bruce, lead author (the manuscript’s guarantor) affirms that the manuscript is an honest, accurate, and transparent account of the study being reported.

Data sharing statement: No additional data is available.

For peer review only

For peer review only - http://bmjopen.bmj.com/site/about/guidelines.xhtml

Figure Legends:

Figure1. Flow chart of the study participants. BiB = Born in Bradford.

† =Total number of eligible BiB children.
‡ =All BiB children who failed vision screening and additionally had a literacy score measured during the same school term.
§=Random sample of BiB children who passed vision screening and additionally had a literacy score measured during the same school term.
*Treatment group=children who failed vision screening and were referred for cycloplegic assessment.
**Adherent=prescribed spectacles worn at each visual acuity assessment.
***Non-adherent=children who failed to attend cycloplegic examination and also children who attended but failed to wear prescribed spectacles at each visual acuity assessment.

Figure 2. Projected visual acuity (logMAR) trajectory (with 95% confidence intervals) by group over time (child's age in months) for the better and worse eye, fully adjusted for all early-life and maternal covariates.

Figure 3. Predicted letter-ID scores over time (child's age in months) based on the trajectories of the visual acuity (adjusted model) of the better eye. The adjusted model includes all early-life and maternal covariates for the comparison, adherent and non-adherent groups.

For peer review only

For peer review only - http://bmjopen.bmj.com/site/about/guidelines.xhtml

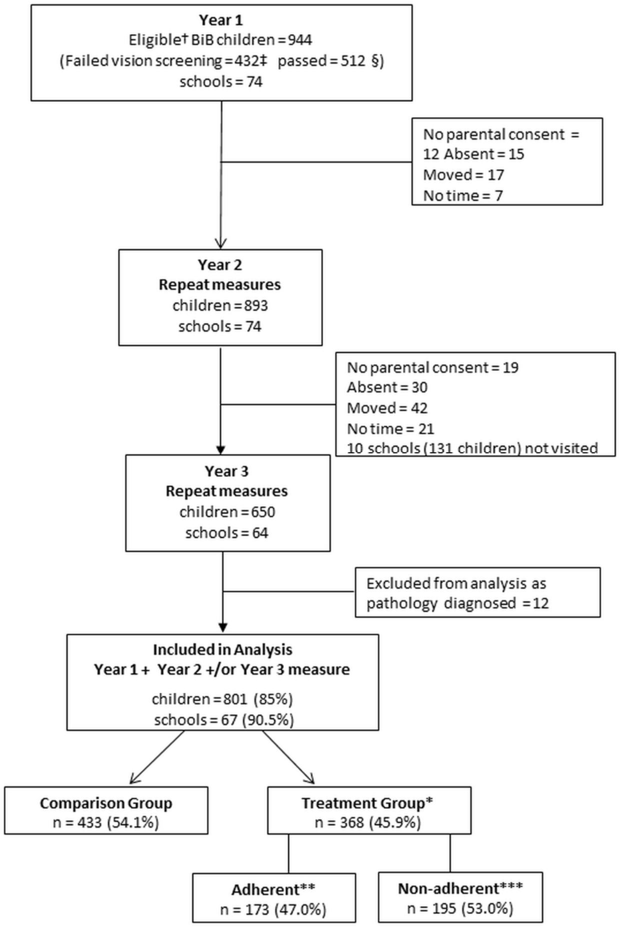

Figure1. Flow chart of the study participants. BiB = Born in Bradford.

† =Total number of eligible BiB children.
‡ =All BiB children who failed vision screening and additionally had a literacy score measured during the same school term.
§=Random sample of BiB children who passed vision screening and additionally had a literacy score measured during the same school term.
*Treatment group=children who failed vision screening and were referred for cycloplegic assessment.
**Adherent=prescribed spectacles worn at each visual acuity assessment.
***Non-adherent=children who failed to attend cycloplegic examination and also children who attended but failed to wear prescribed spectacles at each visual acuity assessment.

190x275mm (300 x 300 DPI)

For peer review only

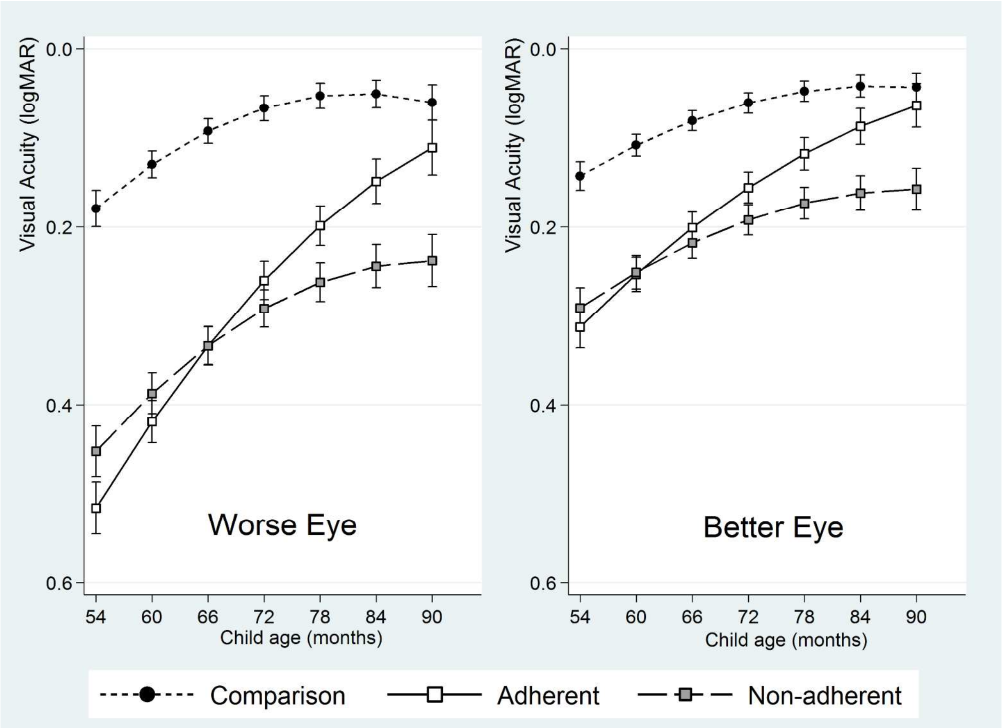

Figure 2. Projected visual acuity (logMAR) trajectory (with 95% confidence intervals) by group over time (child's age in months) for the better and worse eye, fully adjusted for all early-life and maternal covariates.

254x184mm (300 x 300 DPI)

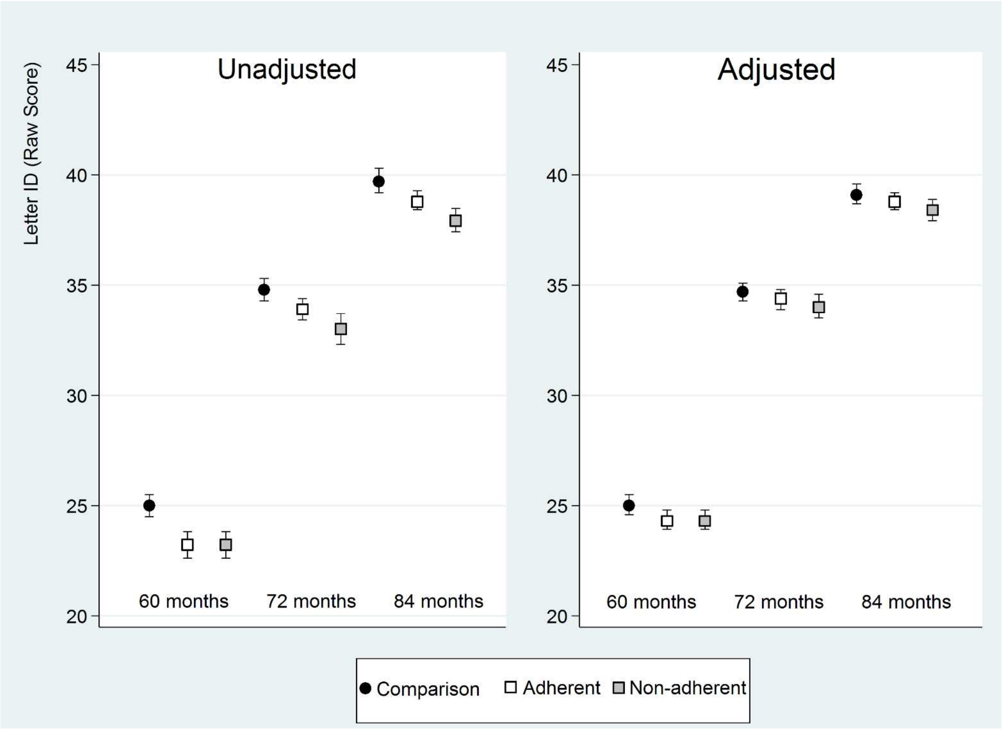

Figure 3. Predicted letter-ID scores over time (child's age in months) based on the trajectories of the visual acuity (adjusted model) of the better eye. The adjusted model includes all early-life and maternal covariates for the comparison, adherent and non-adherent groups.

254x184mm (300 x 300 DPI)

Supplementary Information 1

Keeler Crowded LogMAR Test

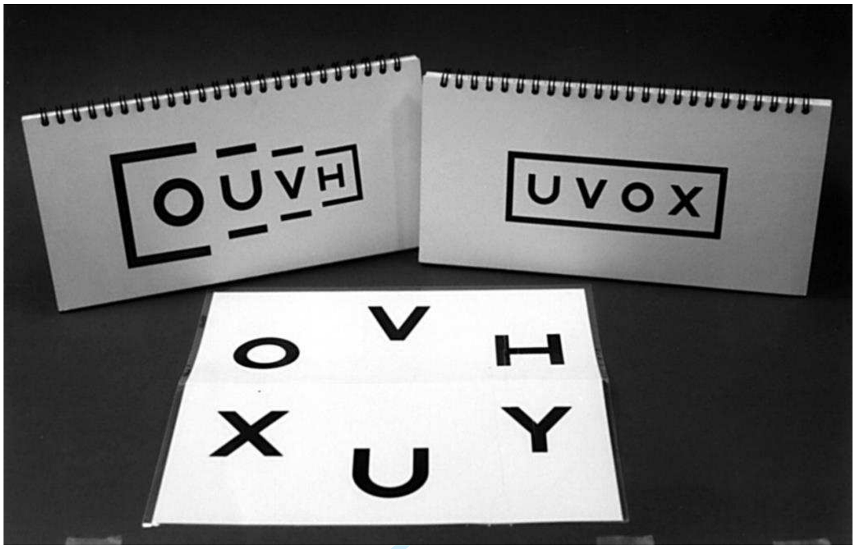

LogMAR Near Vision Test

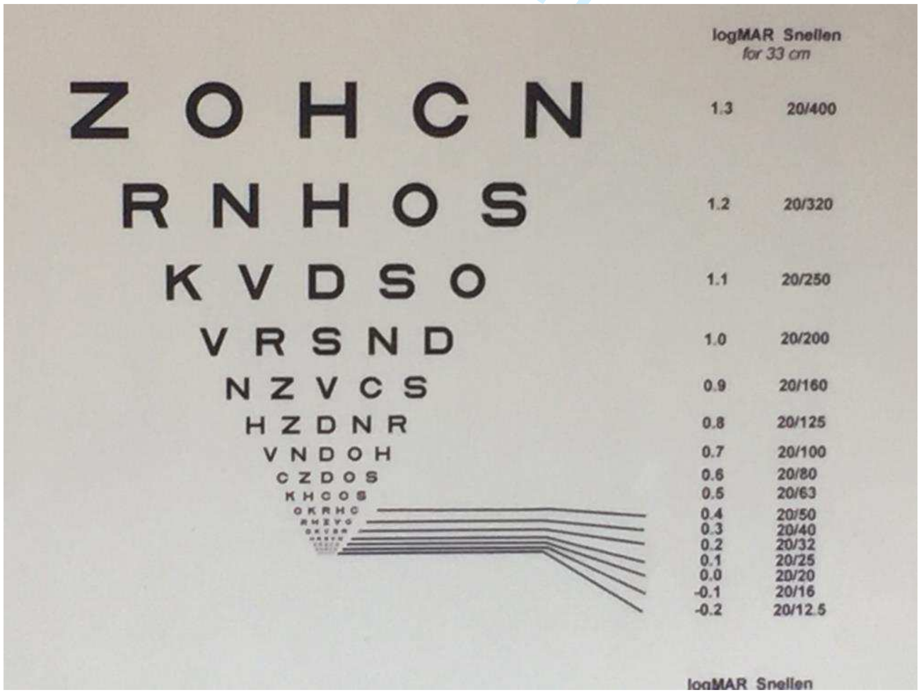

Supplementary Information 2

Associations between Letter-ID score and refractive error types.

FACTOR	FULLY ADJUSTED MODEL (95% CI)	p value
Constant	-21.4 (-29.0 to -13.8)	<0.001
Age	1.32 (1.23 to 1.41)	<0.001
Age squared	-0.021 (-0.023 to -0.018)	<0.001
**Astigmatism**	**-0.329 (-0.933 to 0.275)**	**0.286**
**Hypermetropia**	**-1.071 (-2.586 to 0.444)**	**0.166**
**Myopia**	**1.386 (-2.953 to 5.275)**	**0.531**
**Low hypermetropia**	**0.255 (-0.835 to 1.344)**	**0.647**
Letter ID baseline (Year 1)	0.346 (0.323 to 0.369)	<0.001
BPVS	0.024 (0.004 to 0.044)	0.019
Ethnicity		
Pakistani heritage	0.569 (-0.128 to 1.267)	0.11
Other	1.057 (0.037 to 2.078)	0.042
Gender		
Female	0.667 (0.102 to 1.232)	0.021
Birth weight (per 100g)	0.074 (0.007 to 0.14)	0.029
Gestational age (weeks)	-0.04 (-0.244 to 0.163)	0.698
Receiving Benefits	-0.011 (-0.588 to 0.565)	0.969
Mothers Level of Education		
(higher than A-level)	0.717 (0.11 to 1.325)	0.021
Mothers age at birth (years)	-0.054 (-0.107 to -0.002)	0.042

For peer review only

Supplementary Information 3

Correlation between near and distance visual acuity (Right Eye) at Time Point Three.

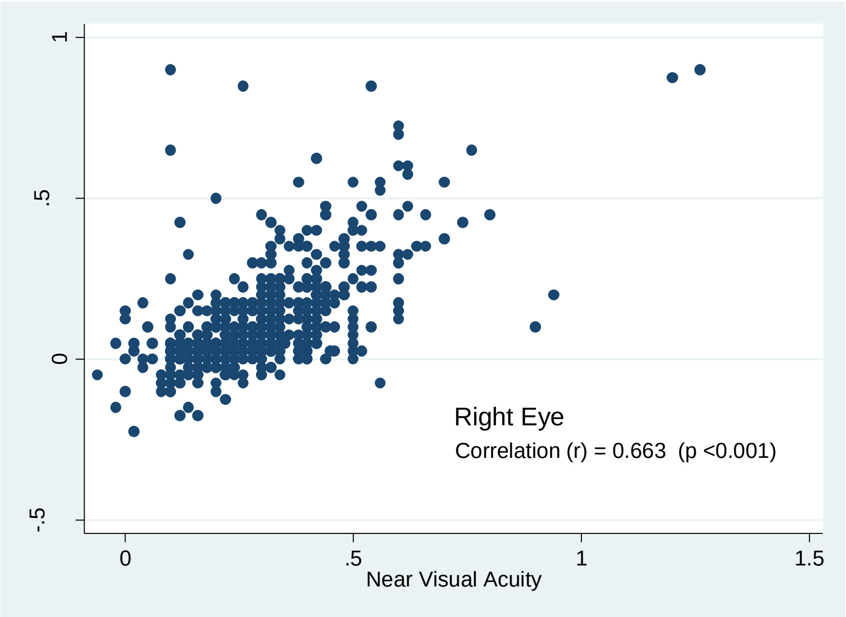

Correlation between near and distance visual acuity (Left Eye) at Time Point Three.

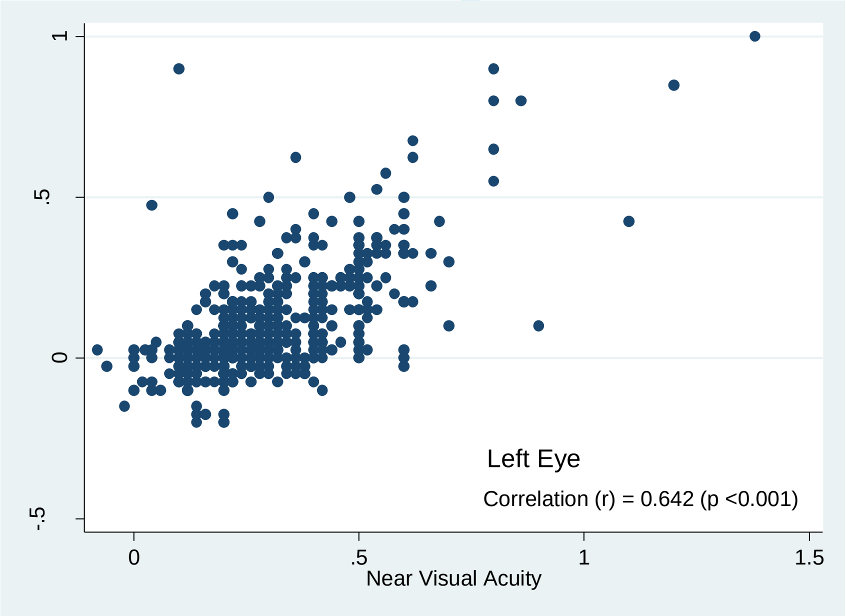

Supplementary Information 4.

Association between visual acuity (distance) and literacy, and between visual acuity (near) and literacy.

	Correlation with Letter ID standardised score at T3	
	**r**	**p-value**
Visual Acuity (far) - Best eye	-0.145	< 0.001
Visual Acuity (far) - Worst eye	-0.183	< 0.001
Visual Acuity (near) – Best eye	-0.115	0.006
Visual Acuity (near) - Worst eye	-0.140	< 0.001

For peer review only

STROBE (Strengthening The Reporting of OBservational Studies in Epidemiology) Checklist

A checklist of items that should be included in reports of observational studies. You must report the page number in your manuscript where you consider each of the items listed in this checklist. If you have not included this information, either revise your manuscript accordingly before submitting or note N/A.

Note: An Explanation and Elaboration article discusses each checklist item and gives methodological background and published examples of transparent reporting. The STROBE checklist is best used in conjunction with this article (freely available on the Web sites of PLoS Medicine at http://www.plosmedicine.org/, Annals of Internal Medicine at http://www.annals.org/, and Epidemiology at http://www.epidem.com/). Information on the STROBE Initiative is available at www.strobe-statement.org.

Section and Item	Item No.	Recommendation	Reported on Page No.
**Title and Abstract**	1	(*a*) Indicate the study's design with a commonly used term in the title or the abstract	
		(*b*) Provide in the abstract an informative and balanced summary of what was done and what was found	
**Introduction**			
Background/Rationale	2	Explain the scientific background and rationale for the investigation being reported	
Objectives	3	State specific objectives, including any prespecified hypotheses	
**Methods**			
Study Design	4	Present key elements of study design early in the paper	
Setting	5	Describe the setting, locations, and relevant dates, including periods of recruitment, exposure, follow-up, and data collection	
Participants	6	(*a*) *Cohort study*—Give the eligibility criteria, and the sources and methods of selection of participants. Describe methods of follow-up<br>*Case-control study*—Give the eligibility criteria, and the sources and methods of case ascertainment and control selection. Give the rationale for the choice of cases and controls<br>*Cross-sectional study*—Give the eligibility criteria, and the sources and methods of selection of participants	
		(*b*) *Cohort study*—For matched studies, give matching criteria and number of exposed and unexposed<br>*Case-control study*—For matched studies, give matching criteria and the number of controls per case	
Variables	7	Clearly define all outcomes, exposures, predictors, potential confounders, and effect modifiers. Give diagnostic criteria, if applicable	

For peer review only

Section and Item	Item No.	Recommendation	Reported on Page No.
Data Sources/ Measurement	8*	For each variable of interest, give sources of data and details of methods of assessment (measurement). Describe comparability of assessment methods if there is more than one group	
Bias	9	Describe any efforts to address potential sources of bias	
Study Size	10	Explain how the study size was arrived at	
Quantitative Variables	11	Explain how quantitative variables were handled in the analyses. If applicable, describe which groupings were chosen and why	
Statistical Methods	12	(*a*) Describe all statistical methods, including those used to control for confounding	
		(*b*) Describe any methods used to examine subgroups and interactions	
		(*c*) Explain how missing data were addressed	
		(*d*) *Cohort study*—If applicable, explain how loss to follow-up was addressed<br>*Case-control study*—If applicable, explain how matching of cases and controls was addressed<br>*Cross-sectional study*—If applicable, describe analytical methods taking account of sampling strategy	
		(*e*) Describe any sensitivity analyses	
**Results**			
Participants	13*	(a) Report numbers of individuals at each stage of study—eg numbers potentially eligible, examined for eligibility, confirmed eligible, included in the study, completing follow-up, and analysed	
		(b) Give reasons for non-participation at each stage	
		(c) Consider use of a flow diagram	
Descriptive Data	14*	(a) Give characteristics of study participants (eg demographic, clinical, social) and information on exposures and potential confounders	
		(b) Indicate number of participants with missing data for each variable of interest	
		(c) *Cohort study*—Summarise follow-up time (eg, average and total amount)	
Outcome Data	15*	*Cohort study*—Report numbers of outcome events or summary measures over time	
		Case-control study—Report numbers in each exposure category, or summary measures of exposure	
		Cross-sectional study—Report numbers of outcome events or summary measures	

For peer review only

Section and Item	Item No.	Recommendation	Reported on Page No.
Main Results	16	(*a*) Give unadjusted estimates and, if applicable, confounder-adjusted estimates and their precision (eg, 95% confidence interval). Make clear which confounders were adjusted for and why they were included	
		(*b*) Report category boundaries when continuous variables were categorized	
		(*c*) If relevant, consider translating estimates of relative risk into absolute risk for a meaningful time period	
Other Analyses	17	Report other analyses done—eg analyses of subgroups and interactions, and sensitivity analyses	
**Discussion**			
Key Results	18	Summarise key results with reference to study objectives	
Limitations	19	Discuss limitations of the study, taking into account sources of potential bias or imprecision. Discuss both direction and magnitude of any potential bias	
Interpretation	20	Give a cautious overall interpretation of results considering objectives, limitations, multiplicity of analyses, results from similar studies, and other relevant evidence	
Generalisability	21	Discuss the generalisability (external validity) of the study results	
**Other Information**			
Funding	22	Give the source of funding and the role of the funders for the present study and, if applicable, for the original study on which the present article is based	

*Give information separately for cases and controls in case-control studies and, if applicable, for exposed and unexposed groups in cohort and cross-sectional studies.

Once you have completed this checklist, please save a copy and upload it as part of your submission. DO NOT include this checklist as part of the main manuscript document. It must be uploaded as a separate file.

For peer review only
